# Towards next generation enzyme mimics through local electric field control in organic cage catalysts

**DOI:** 10.1039/d6sc04940k

**Published:** 2026-10-02

**Authors:** Marco Vitek, Igor Rončević, Keith G. Andrews

**Affiliations:** a Institute of Organic Chemistry and Biochemistry of the Czech Academy of Sciences Flemingovo nám. 542/2 160 00 Prague 6 Czechia; b Department of Chemistry, University of Manchester Oxford Road Manchester M13 9PL UK igor.roncevic@manchester.ac.uk; c Department of Chemistry, Durham University Lower Mount Joy, South Rd Durham DH1 3LE UK keith.g.andrews@durham.ac.uk

## Abstract

Electric fields (EFs) can, in principle, accelerate chemical reactions by preferentially stabilising charge distribution in transition states. In practice, strategies for aligning tuneable EFs across reaction sites in artificial molecular systems remain scarce. Here, using modelling, we establish the potential of dipolar supramolecular cavities as an approach to quantify and optimise solution-phase EF catalysis. By positioning pairs of charged substituents on a cage catalyst exterior, we align tuneable, oriented, local fields with reaction dipoles without introducing through-bond effects. We introduce a finite capacitor model to evaluate EFs in dipolar cavities and show cavities of 1 nm can generate EF strengths comparable to enzymes (∼0.2 V Å^−1^). We validate this model computationally by quantifying EF-dependent catalysis in synthetically accessible analogues of an existing acyl-transfer cage catalyst, obtaining a large 13 kcal mol^−1^ per V Å^−1^ dependency. Roughly half of the generated field interaction energy is translated into catalysis, yielding a projected rate acceleration of two orders of magnitude. Optimal EF catalysis required constraining and aligning both the reactant complex and transition state in the EF, suggesting that enzymes may employ strong binding to maximise EF catalysis by preserving electrostatically strained substrate configurations in local fields.

## Abstract 2

The addition of charged substituents across an organic cage catalyst creates local oriented electric fields, comparable in strength to enzyme active sites, that reduce an acyl transfer reaction barrier by ∼3 kcal mol^−1^, according to DFT/CC modelling.

## Graphical abstract

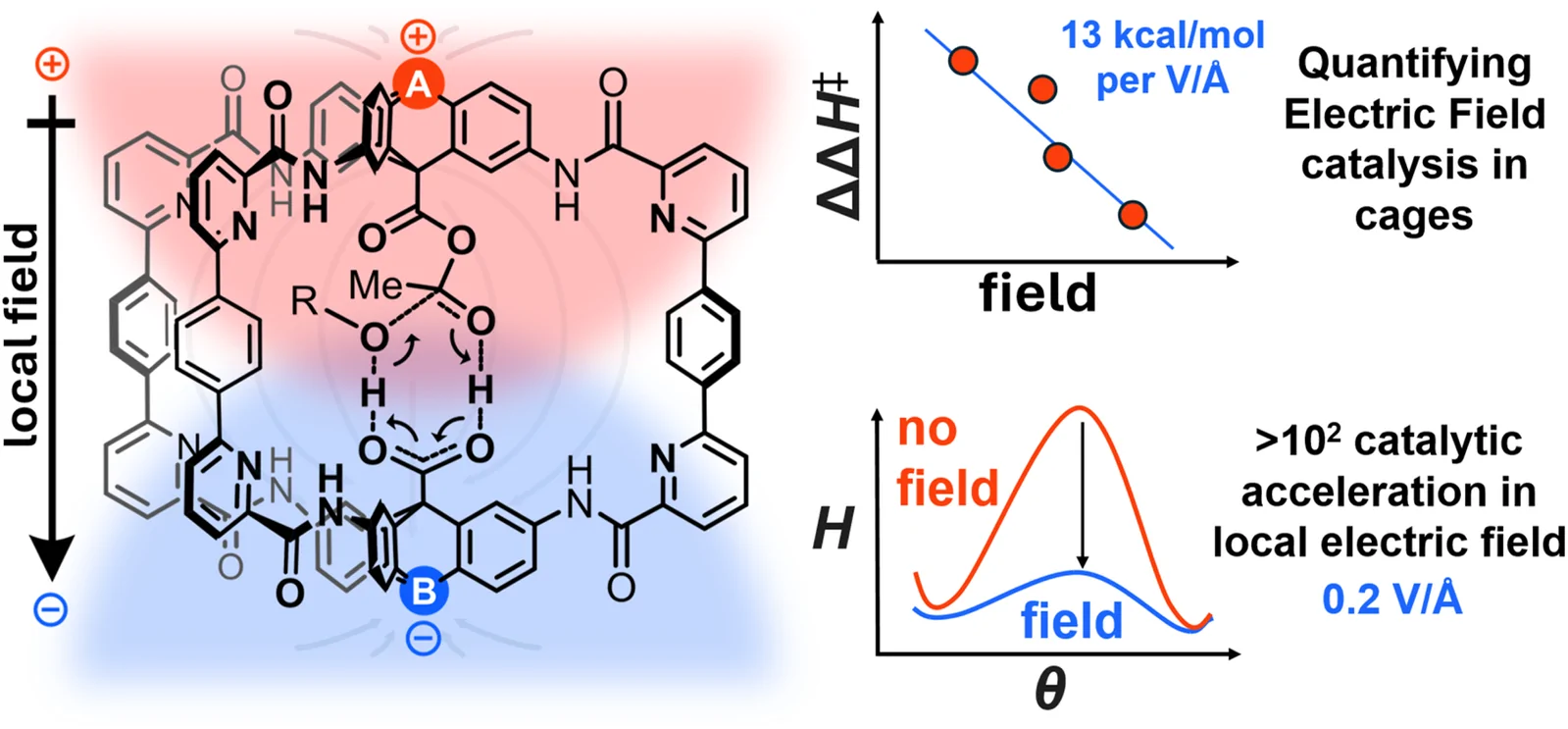

## Introduction

Oriented electric fields (EFs) in enzyme active sites are increasingly recognised to enhance catalytic efficiency, primarily by stabilising the transition state (TS) dipole relative to the reactant-complex (RC) dipole (Fig. 1a–c).^1–7^ Although this “EF catalysis” is theoretically well-grounded using external fields^8–12^ and in enzymes,^4,13,14^ experimental exploitation in artificial systems with local fields remains challenging due to the difficulty in aligning the field with respect to a reaction. Further, most synthetic catalysts lack the architectural precision to robustly control locally-generated through-space EFs at the reaction site in solution, and rely on through-bond activation.^15^ Therefore, molecular strategies to optimise catalysis using designed EFs remain underexplored,^4,16–28^ in part because their potential remains unquantified.^29,30^ Here, we describe a supramolecular approach to control and quantify local oriented EFs in chemical catalysis using charges arranged across the cavity of an organic cage enzyme mimic.^31,32^

**Fig. 1:**
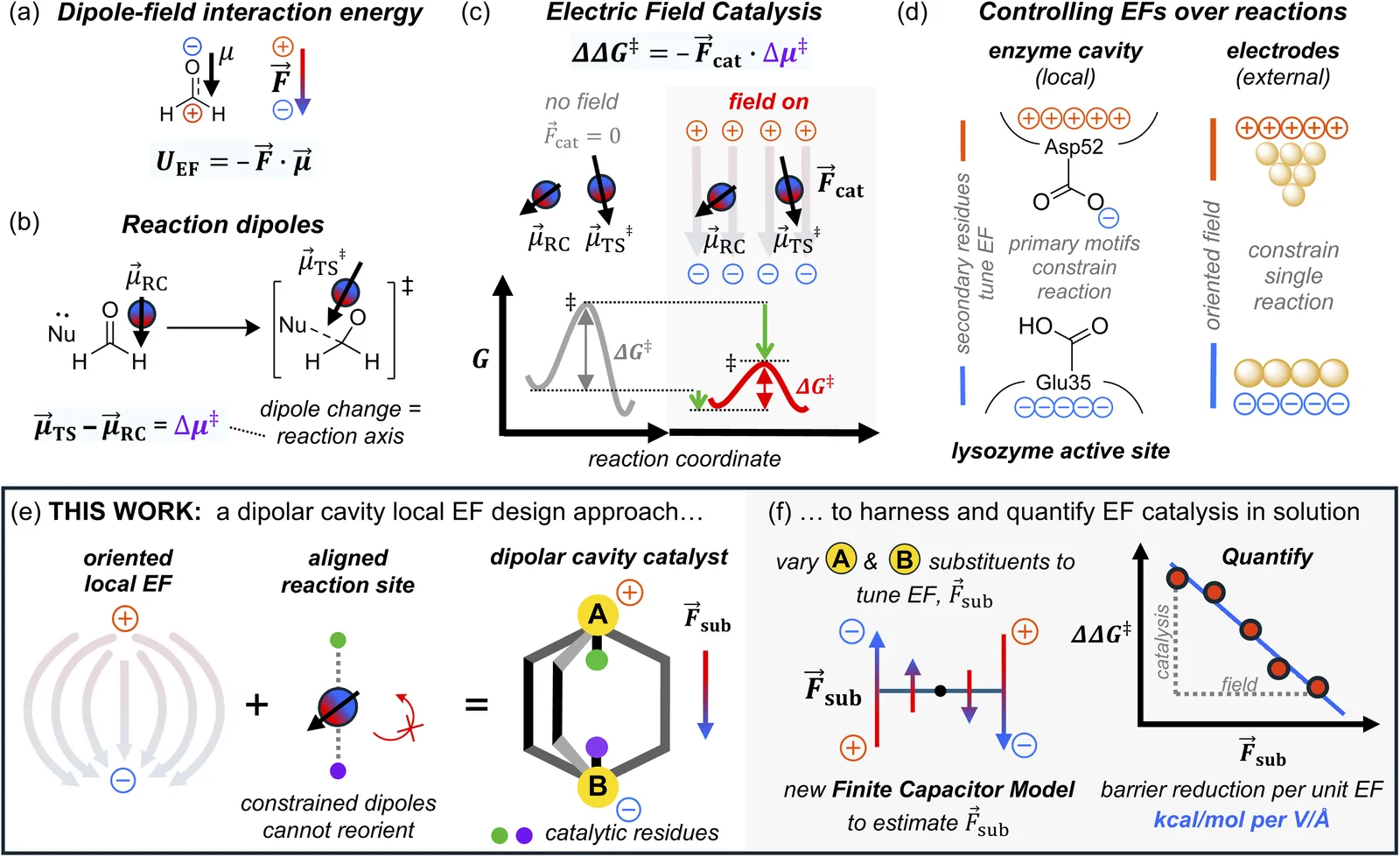
(a) Molecular dipoles *µ* interact with EFs *F* with interaction energy *U*_EF_ dependent on field strength, dipole strength, and alignment. All vectors follow the physics conventions: field vectors point from positive to negative and dipole moments from negative to positive. (b) The change in dipole between reactant complex (RC) and transition state (TS) is called the reaction axis. (c) General approach to electric field catalysis. Preferential stabilisation of the transition state dipole *µ*_TS_ over the reactant-complex dipole *µ*_RC_ by an oriented electric field *F*_cat_ leads to reaction acceleration by reduction of the barrier, Δ*G*^‡^. (d) The local EF generated in the lysozyme enzyme and an electrode approach to control an external EF across a reaction site. Controlling local EFs in synthetic catalysts remains challenging. (e) The general design concept to control EFs over reactions in solution explored in this manuscript: a dipolar cavity with charged substituents A and B orients an EF across a fixed internal reaction site. (f) Variation of the local EF *F*_sub_ by varying charged groups A and B allows the pure effect of the field on the catalytic barrier to be measured.

Enzymes constrain substrates with primary motifs, and tune strong EFs with secondary residues,^23^ as in the case of lysozyme (Fig. 1d).^33,34^ In this way, enzymes can favour stabilisation of the TS dipole despite the need for reactant complex stabilisation,^6,35^ with estimates of 72% of the catalytic barrier correlated with the EF in some cases.^6,36–38^ In synthetic systems, unconstrained reactants will reorganise in EFs, forfeiting control of orientation,^10^ although general electrostatic effects^20,39–41^ are still widely employed in catalysis.^42–50^ Therefore, approaches to immobilise substrates to align them in EFs tuned by secondary sources have been investigated, with the goal of demonstrating EF-dependent catalysis.^4,16–30^ Coote and others have immobilised reagents between electrodes that generate an oriented field to control catalysis in single molecule experiments (Fig. 1d).^51–56^ Parallel plate capacitor systems employ frozen liquids to prevent solution-phase molecular reorganisation,^57–59^ and interfaces can orient fields and molecules.^60,61^ Despite the effective use of electrostatics in cavity catalysis,^62–75^ general strategies to align local EFs over constrained reactants to quantify and enhance EF-catalysis in synthetic systems remains undeveloped.^76^

Here, we unite advances in EF theory^3,4,16–28^ and supramolecular catalysis to computationally examine a general approach to control local EF catalysis in solution. Our design involves organising pairs of charged substituents A and B (secondary residues, yellow) across a cavity relative to catalytic groups (primary motifs, green and purple, Fig. 1e). Since the catalytic groups can constrain the reaction complex and transition state, and the field can be independently tuned, the design permits exploration of the effect of oriented EFs on catalysis. For instance, modulating the EF by tuning substituents A and B would allow the through-space effect on the catalytic barrier to be unambiguously quantified (Fig. 1f), clarifying the results from enzymes,^6,13–15,36–38^ and revealing design rules for synthetic EF catalysts.

To validate our conception, we explore analogues of a recently reported organic cage catalyst (Fig. 2a).^31,32,77–79^ Using theory and computation, we demonstrate that introduction of charges at the triptycene bridgehead positions A and B (Fig. 2b) modulates catalytic barriers for an internal acyl transfer reaction (Fig. 2c and d) by as much as ±3 kcal mol^−1^ (2 orders of magnitude enhancement over the reported catalyst, Fig. 3). We then use field-dipole models to show that the barrier changes are linearly proportional to EF strength in the cavity and dependent on orientation (Fig. 4). To estimate the local fields in the cages, we introduce a finite capacitor model, a tool requiring only charge and distance as input parameters, valid for any system utilising our conceptual design (Fig. 5). We then discuss the chemical consequences of the EFs in the studied cages and show that through-space effects dominate classical through-bond intuitions (Fig. 6). Finally, we discuss opportunities and challenges associated with applying tuneable EFs in solution, and in translating these designs into real experimental systems, and conclude that explicitly designed local EFs in experimentally feasible cavities are poised to transform opportunities in catalysis.

**Fig. 2:**
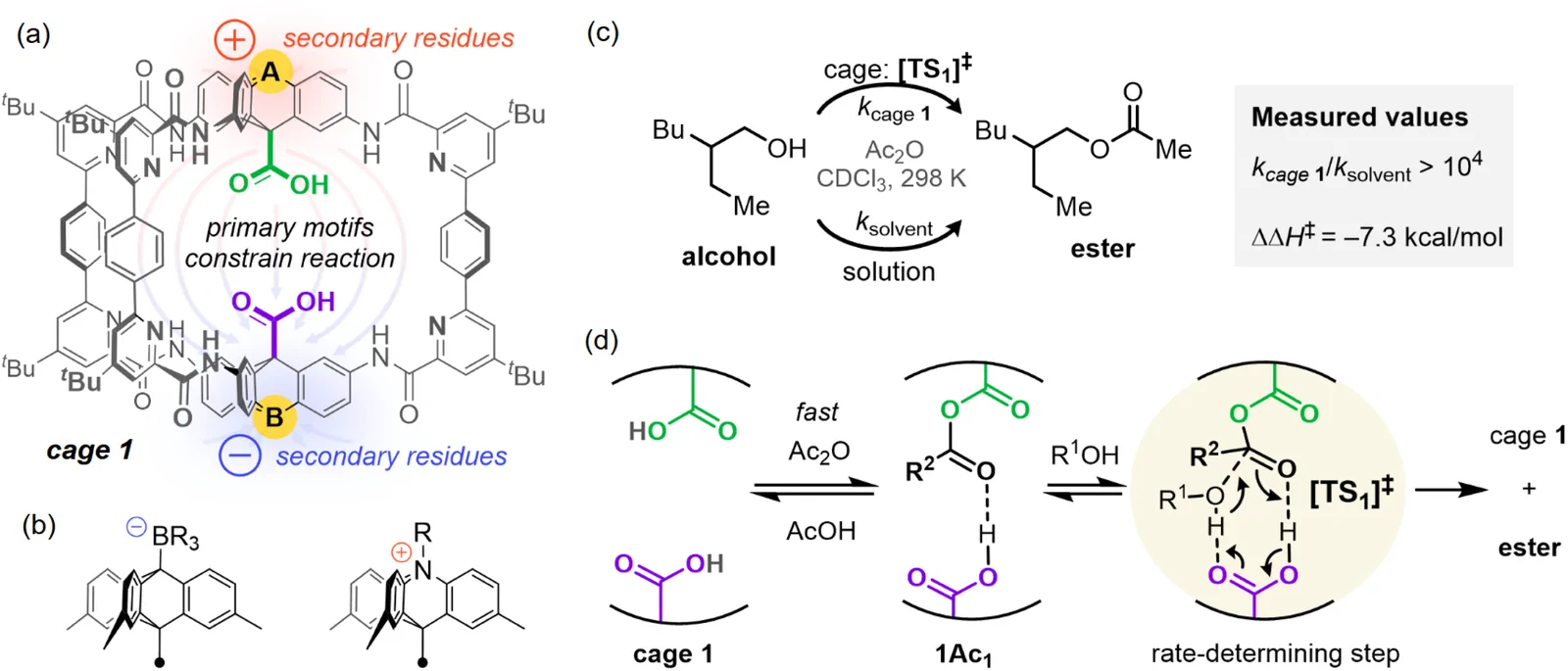
(a) Andrews' neutral enzyme mimic cage 1 (A = B = CH) is used to validate the dipolar cavity concept for EF catalysis quantification.^31^ (b) Examples of substituted triptycenes with charged groups A and B to generate the local EFs. (c) The previously experimentally-benchmarked esterification (acyl transfer) reaction examined to test the EF concept here. The reaction is accelerated by cage 1 (298 K, CDCl_3_) by 10^4^ compared to the bulk solvent “background” reaction (ratio of second order rate constants, *k*_cage_/*k*_solvent_).^31^ (d) The mechanism of the acyl transfer catalysis in cage 1 begins with rapid equilibration of cage 1 and acetic anhydride to form acetic acid and acylated cage intermediate, 1Ac_1_. 1Ac_1_ engages in weak binding with alcohol to form a hydrogen-bonded reactant complex (see also SI, Section S2). Finally, rate-determining acyl transfer to form ester is mediated *via* a well-defined transition state, **TS^‡^_1_**.^31^ The work herein calculates the effect of EFs on the barrier between **TS^‡^_1_** and the reactant complex preceding it.

**Fig. 3:**
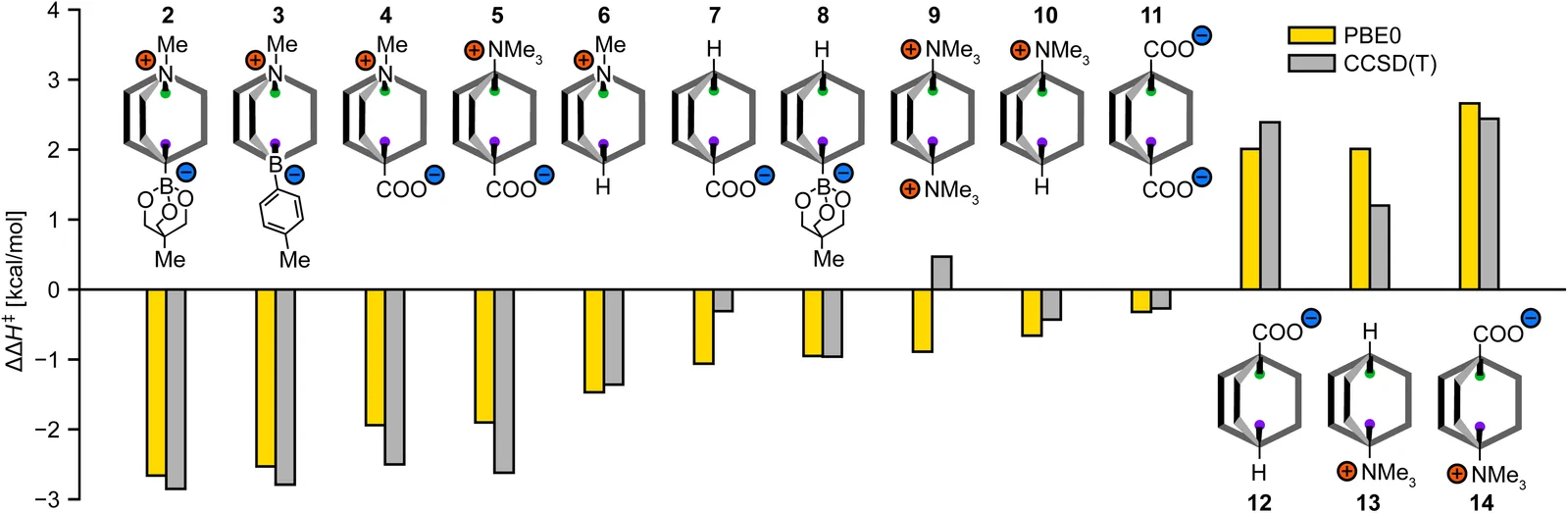
Calculated reaction barrier changes (ΔΔ*H*^‡^) for substituted cages 2–14 relative to cage 1 for the acylation reaction (**TS^‡^_1_**) in Fig. 2 obtained using density functional theory (yellow bars) and coupled clusters (grey bars). Substitutions are made at positions A and B, at, or attached to, the bridgehead atom (see Fig. 2b). Cages with positive charges closer to the anhydride (upper green dot) and negative charges closer to the carboxylic acid (purple dot) show barrier reductions of up to −3 kcal mol^−1^ (*i.e.* cage 2), and are therefore predicted to accelerate catalysis over the experimentally benchmarked parent cage 1. Structures 5/14, 10/13 and 7/12 are the same cage structure, with the A and B groups reversed relative to the anhydride.

**Fig. 4:**
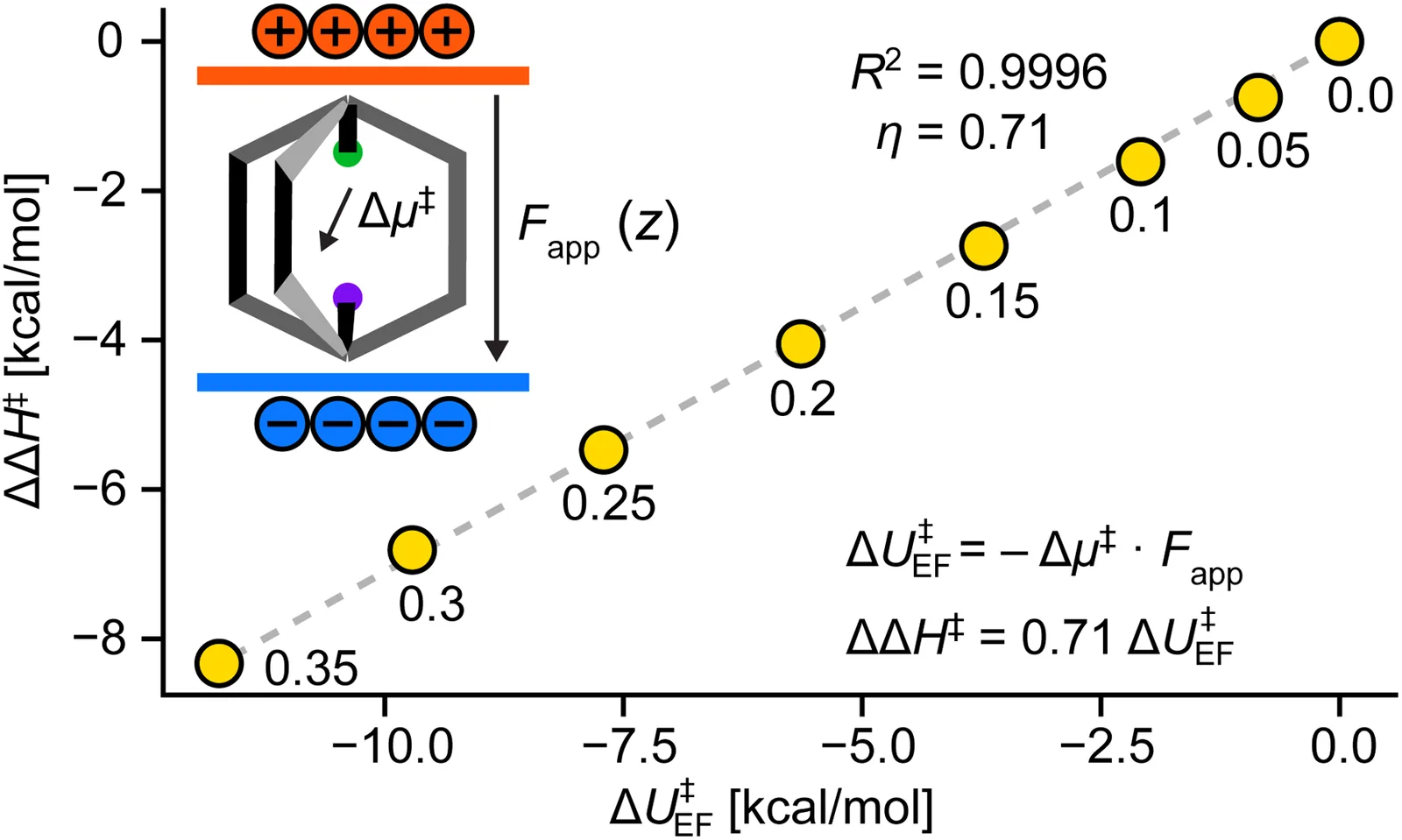
PBE0-calculated changes in acyl transfer activation enthalpy ΔΔ*H*^‡^ in unsubstituted cage 1 for TS_1_^‡^ in externally applied EFs along *z*, showing a 71% conversion of the EF–dipole interaction Δ*U*^‡^_EF_ to ΔΔ*H*^‡^. Points are labelled with their EF strength (in V Å^−1^).

**Fig. 5:**
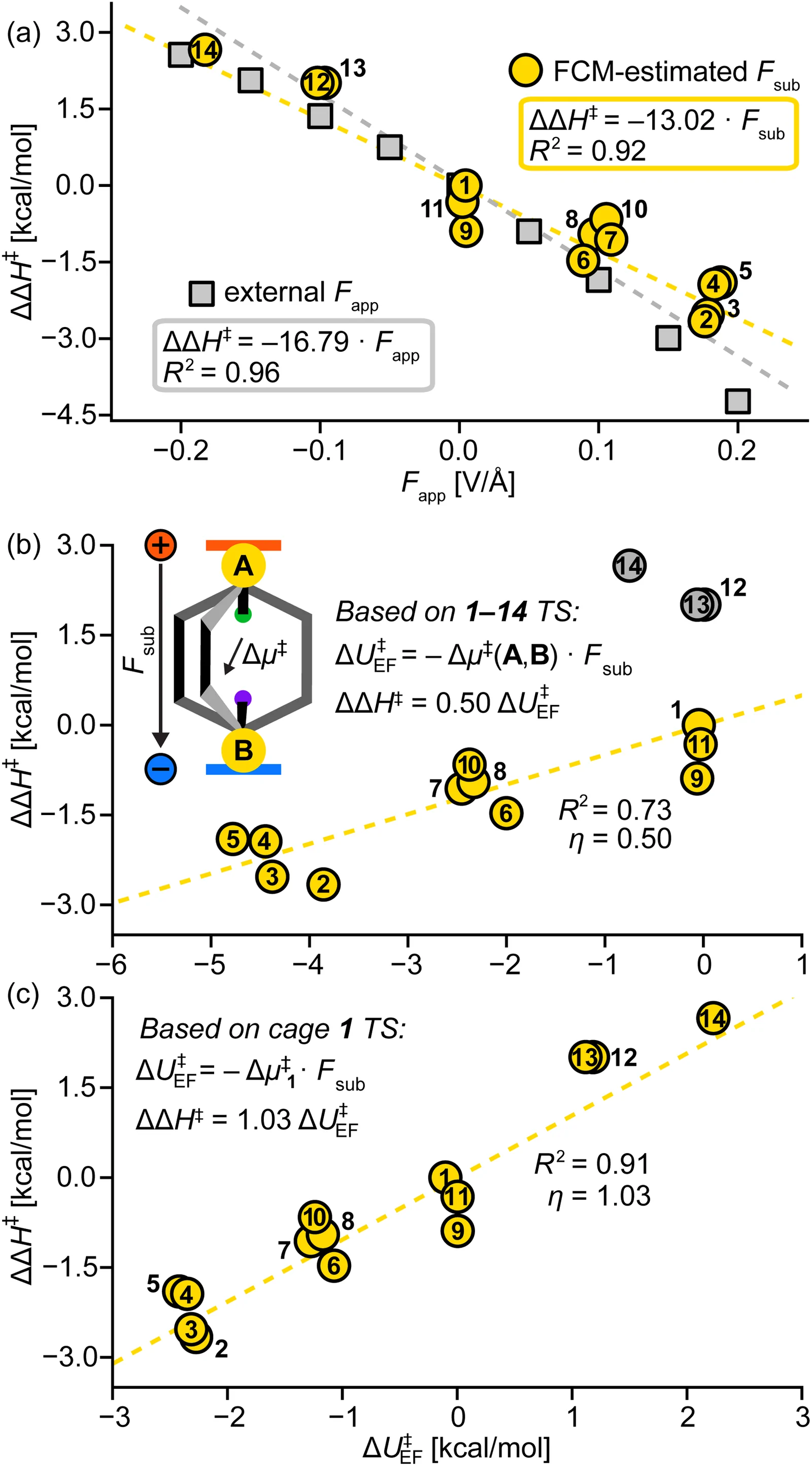
Validation of FCM. (a) Comparison between FCM-estimated local substituent fields *F*_sub_ (yellow) and externally applied field *F*_app_ (grey) and ΔΔ*H*^‡^ for cages 1–14 shows strong agreement. (b and c) Effect of substituent-induced EFs (Δ*U*^‡^_EF_) on the acylation barrier change ΔΔ*H*^‡^ relative to cage 1 computed using: (b) the activation dipole moment of each individual cage (2–14), or (c) the activation dipole moment of cage 1, *i.e.* to estimate the change in catalysis without using the TS or RC structures for cages 2–14. The regression line in (b) excludes cages 12–14 (grey).

**Fig. 6:**
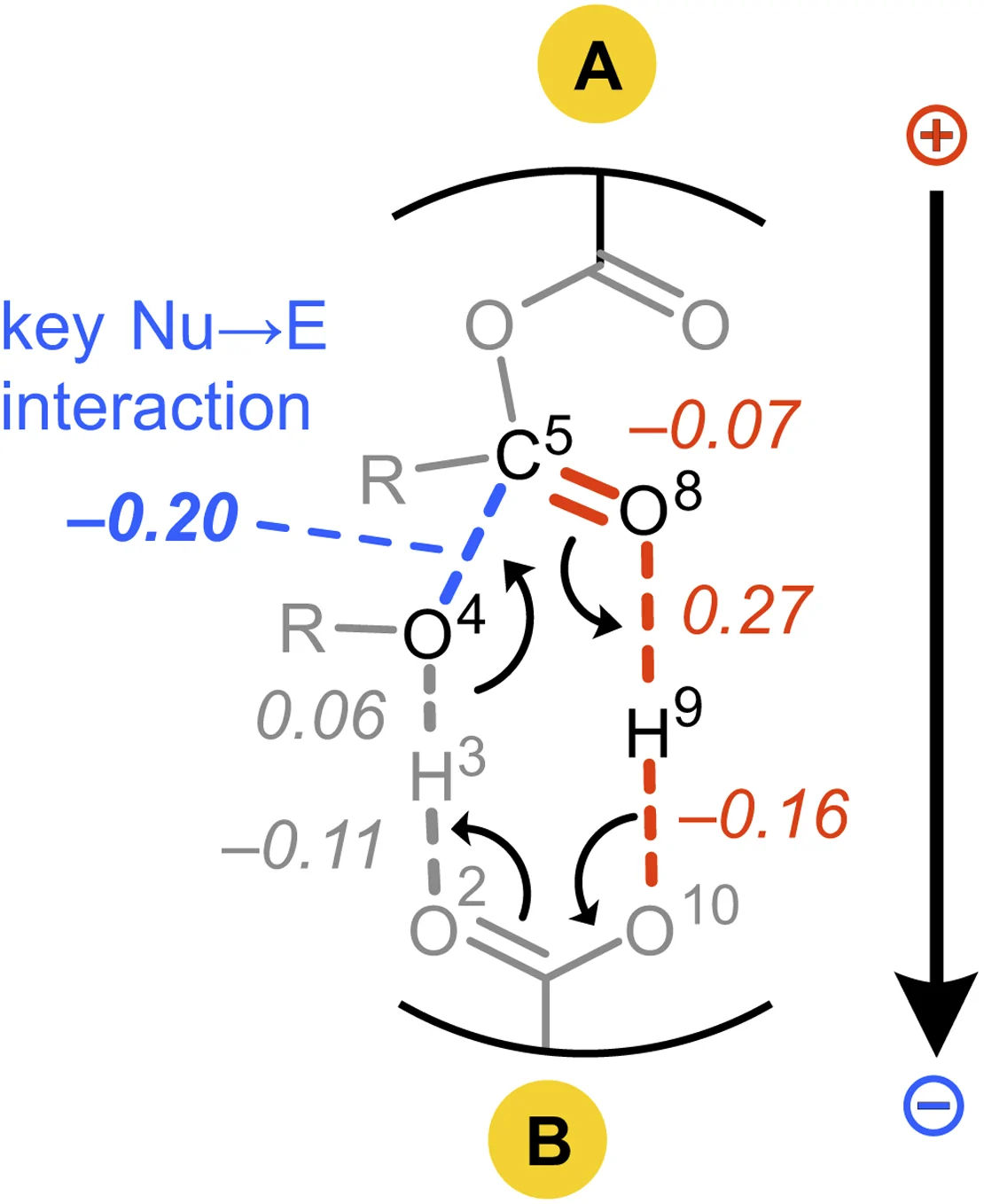
Mass-weighted bond length changes (Å) in TS_1_^‡^ for charged cage 2 relative to zero field cage 1 show the key role of the nucleophile (Nu) to electrophile (E) interaction along the electric field despite competing changes elsewhere. Negative numbers indicate a shorter bond in cage 2. Blue indicates field-induced reaction progress; red indicates field-induced reaction regress (compared with zero-field).

## Results and discussion

### Model system for quantifying EF catalysis

Local EF catalysis in artificial molecular systems has been championed by Coote and Shaik in theoretical work.^16,29,30,39,76^ Here we seek to contribute general molecular design rules to facilitate the push towards experimental implementation. Neutral programmable organic cage catalyst 1 (ref. 31, 32 and 77–79) (Fig. 2a and S1, A = B = CH), previously developed by one of us, was selected as a model system to explore the design concept in Fig. 1e. Unoriented electrostatic catalysis in cages is well-explored,^62–75^ including in cages with a convenient *C*_2_ axis, and application of our approach may be applicable where desymmetrisation and reaction orientation can be developed. We reasoned that the following properties of cage 1 and its analogues make it suitable for studying electric field catalysis:

(1) Plausible cavities hosting defined reactions: cage 1, a lysozyme active site structural mimic, catalyses organocatalytic acyl transfer using two endohedral^80^ carboxylic acid groups (Fig. 2d). This two-point interaction constrains the reaction axis (Fig. 1b) with respect to the cage triptycenyl axis.^32^ The acyl transfer step involves concerted proton transfer and so is expected to be electrostatically sensitive.^63,81,82^

Synthetic access to versions of cage 1 with different A and B triptycenes has already been achieved.^31^ Bridgehead substituted triptycenes (Fig. 2b)^83–85^ are well-described due to their importance in materials science.^86,87^ Large molecular cages such as 1 can use tuneable side-chains (*e.g.*^*t*^Bu) for solubility or to sterically protect charged groups and reduce charge-screening in the cavity.

(2) Tuneable fields: the A–B-substituted cores of the triptycene units of cage 1 resemble bridgehead-substituted bicyclo[2.2.2]octane-1-carboxylic acids, which are known to modulate the acid p*K*_a_ through-space (*i.e.* field effects)^88,89^ rather than through 6–8 sigma bonds (see Fig. S4 and associated discussion).^90,91^ Like an enzyme pocket, the compact nature of the cage cavity limits solvent access, reducing internal charge screening. In any case, chloroform (*ε*_r_ = 4.8) has a low dielectric constant, comparable to many protein models (*ε*_r_ = 4.0),^92^ and much lower than water (*ε*_r_ = 78.4), and is only a weak hydrogen-bond acceptor. Use of large, non-coordinating counterions can limit charge-screening in cage cavities to 5–15% (Fig. S11, and discussion), and are frequently employed in chlorinated solvents.^67,75^

(3) Alignment of EF and reaction: cage 1 strongly aligns the A–B charged groups with the reaction axis^10^ since the A–B (*z*−) axis is conformationally constrained due to pyridine–amide interactions.^31,32,77^ Molecular dynamics simulations of cage 1 (298 K, chloroform)^31^ show deviations from collinearity of only 3%, and an acid–acid distance-variance of 0.29 Å due to twisting along the triptycene–triptycene axis (Fig. S5).

(4) Minimise through-bond effects: the A and B groups are external to the reaction site, and through-bond interference is therefore unlikely, which is important for unambiguous quantification of EF catalysis. Further, there are no local flexible groups that might otherwise interfere.

Although implementation of ideal conditions for EF catalysis is challenging, we deem the above criteria a significant step towards a general design.

### The acyl transfer catalysis studied

The mechanism of acyl transfer catalysis (Fig. 2c and S2) in neutral cage 1 (Fig. 2a) was previously studied experimentally and proceeds in two steps. First, an anhydride acyl-donor (*e.g.* Ac_2_O) enters a rapid equilibrium with acylated cage intermediates 1Ac_1_, with an anhydride reversibly forming at the top or the bottom carboxylic acid (Fig. 2d). The second, rate-determining step involves internal transfer of the acyl group to a weakly bound alcohol substrate (Fig. 2d). Experimental Eyring analysis indicated the reaction is enthalpically stabilised (ΔΔ*H*^‡^ = −7.3 kcal mol^−1^) and a DFT investigation identified well-organised transition state **TS^‡^_1_** that closely matches the experimental value for Δ*G*^‡^_TS1_ and features synchronous proton transfer between the two aligned cage carboxylate groups (Fig. 2d). The rate-enhancement over the solution-phase reaction (*k*_cage__1_/*k*_solvent_) can exceed 10^4^.^31^ We therefore set out to quantify the effect of charged-analogues of cage 1 on the rate-determining step using the previously benchmarked^31^ computation to validate our conceptual design.

### Quantifying catalysis in local EFs

We designed seventeen additional synthetically plausible model variants of cage 1 with charged A and B substituents on the triptycene groups (*e.g.* as in Fig. 2b) predicted to generate internal fields (2–14 shown in Fig. 3, 15–18 in SI Fig. S8), and calculated the change in activation barrier **TS^‡^_1_** relative to cage 1, ΔΔ*H*^‡^ (see Methods), in the absence of counterions, for the acyl transfer reaction shown in Fig. 2d. We adopt a convention to place the internal anhydride at the top of the cage (green dot, Fig. 3).

DFT (PBE0-D3BJ/def2-SVP/SMD(CHCl_3_)) and coupled cluster (CC) calculations (DLPNO-CCSD(T)/cc-pVTZ/C-PCM(CHCl_3_)) (yellow and grey bars in Fig. 3) indicate that charged substituents modulate the enthalpic barrier by ±3 kcal mol^−1^ (or more, see Fig. S6). A positive-to-negative (top-to-bottom) dipolar field reduces the barrier (ΔΔ*H*^‡^) most (*e.g.* cages 2–5), providing a rate constant enhancement factor of 10^2^ above cage 1 (10^6^ over the solvent-promoted reaction).^31^ This value, if reproducible in experiment, indicates the remarkable potential of local EFs for order-of-magnitude enhancement of organocatalyst activity. The increased ΔΔ*H*^‡^ barriers for cages 12–14 (and the derivatives of cages 2, 3, 4, 6 where the substituents A and B are reversed), cages 15–18 (not shown, see SI Fig. S8) will be discussed later. We additionally verified that the collapse of the tetrahedral intermediate did not become rate-determining (Fig. S9), and that the pre-equilibrium cage-acylation to form 1Ac_1_ (Fig. 2d) was not strongly affected (Table S1). The effect of the EF catalysis is inversely sensitive to the solvent dielectric constant (*ε* = 2.37, 4.71, 7.42): −4.25 kcal mol^−1^ in toluene, −2.66 in CHCl_3_, and −2.26 in THF (Fig. S10). Further, in the presence of counterions (K^+^/Na^+^ and I^−^/Cl^−^), we estimate catalysis to be slightly attenuated (up to 0.7 kcal mol^−1^ for cage 2, 20%, Fig. S11). Next, we turned to quantifying the fields across the cage family.

### Quantifying the fields

We next sought to test the hypothesis that the reaction barrier changes for the acyl transfer reaction (**TS^‡^_1_**) in cages 2–14 (Fig. 3) are rationalised by the theory of electric field catalysis (*i.e.* are EF dependent). We will briefly summarise the pertinent theory of EF catalysis.^10,37^ The interaction energy *U*_EF_ between an applied electric field *F*_app_ and an electric dipole *µ* is given by:

1 *U*_EF_ = −*F*_app_·*µ*

where *F*_app_ and *µ* are vectors, meaning *U*_EF_ is more negative (favourable) when *F*_app_ and *µ* coalign and when their magnitudes are large. Eqn (1) describes a linear field–dipole interaction, but non-linear effects such as polarisability may be implicitly included through the dependence of *µ* on *F*_app_; for example, a molecule may develop more dipolar character in a strong, well-aligned *F*_app_.

In a reaction proceeding through a transition state (TS) from a reactant complex (RC), the change in activation interaction energy due to the application of a constant external field *F*_app_ is given by:

2 Δ*U*^‡^_EF_ = −*F*_app_(*µ*_TS_ − *µ*_RC_) = −*F*_app_·Δ*µ*^‡^

In eqn (2), Δ*U*^‡^_EF_ is the change in the field–dipole interaction energy of the TS relative to the RC. It therefore follows that if the EF is preferentially aligned with the reaction axis – the direction of the vector Δ*µ*^‡^ – a more favourable change in interaction energy will result. Or in more familiar terms: application of a well-oriented field *F*_app_ can stabilise the TS dipole over the RC dipole, providing a way for an EF to reduce the reaction barrier.

The change in the activation enthalpy in field *F*_app_ relative to a reference field *F*_0_ can be calculated as:

3 ΔΔ*H*^‡^_F–0_ = (*H*^‡^_F_ − *H*^RC^_F_) − (*H*^‡^_0_ − *H*^RC^_0_) = Δ*H*^‡^_F_ − Δ*H*^‡^_0_

where ΔΔ*H*^‡^_F–0_ is the change in the activation enthalpy for **TS^‡^_1_** in a field Δ*H*^‡^_F_ relative to the activation enthalpy in a zero field reference Δ*H*^‡^_0_ (*e.g.* the reaction barrier for cage 1). Therefore ΔΔ*H*^‡^ (dropping the subscript for generality) is a measure of field-dependent effects on the reaction barrier.

An interesting question in EF catalysis is how well the simple field–dipole interaction Δ*U*^‡^_EF_ (eqn (2)) translates to the experimentally measured (or *ab initio* calculated) change in enthalpy ΔΔ*H*^‡^ (eqn (3)). The response of an electric field to a molecule clearly goes beyond the first-order, redistributing electron density in all bonds and therefore changing the geometry and multipole (quadrupole, octapole…) moments. To phenomenologically account for these effects, we define a field-response factor *η*:

4 ΔΔ*H*^‡^ = *η*Δ*U*^‡^_EF_

which encompasses various higher-order changes within the geometry and electronic structure beyond the field–dipole interactions, as well as changes in the vibrational structure and solvent–solute interactions affecting enthalpy (and in principle entropy). At *η* = 0, higher-order effects dominate and the electric field does not affect the reaction barrier; if *η* = 1, higher-order effects are negligible and the observed change in barrier is fully converted to the barrier change. Therefore 1 − *η* is the extent of attenuation of the first-order field–dipole response, and *η* may be viewed as the “efficiency” with which an external field is converted into catalytic activity, analogously to *ρ* in Hammett-type plots.

### Application of an external EF to neutral cage 1

To probe the hypothesis that the changes in reaction barrier for the acyl transfer reaction (**TS^‡^_1_**) in cages 2–14 (Fig. 3) can be rationalised by the theory of electric field catalysis, we calculated the reaction barriers for the acyl transfer reaction inside neutral cage 1 in the presence of external (non-locally generated) electric fields. This experiment probes EF effects independently of any potential structural or through-bond effects in cages 2–14.

Following Stuyver,^93^ a constant external field *F*_app_ can be produced along the *z*-axis using a parallel plate capacitor. The reactant complex and transition state geometries were optimised in such uniform, unscreened, externally applied fields *F*_app_ between ±0.35 V Å^−1^. Cage 1 is experimentally benchmarked, and so we use DFT (PBE0-D3BJ/def2-SVP/SMD(CHCl_3_)) (see Methods, and Section S1 in the SI).^31^ From the resultant structures we extracted the dipole moments of the TS *µ*_TS_ and the reactant complex *µ*_RC_ which enabled us to calculate the activation dipole moment Δ*µ*^‡^ and the change in the field–dipole interaction energy Δ*U*^‡^_EF_ using eqn (2).

The calculated activation dipole moment Δ*µ*^‡^ aligns almost exactly with the *z*-axis (Fig. S3). In other words, the reaction axis is ideally oriented to interact with a field *F*_app_ applied in the *z*-axis, and indeed with charged substituents at position A and B.

We next computed ΔΔ*H*^‡^, the change in the activation enthalpy for **TS^‡^_1_** relative to the zero field (Table S2), at the different field strengths using eqn (3). We find that ΔΔ*H*^‡^ becomes more negative (a reduced reaction barrier) linearly (*R*^2^ = 0.9985, Fig. S12) with increasing *F*_app_ up to high positive field strength (+0.35 V Å^−1^, positive top, negative bottom). A negative field correspondingly increases ΔΔ*H*^‡^ (Fig. S13 and S14). The field-response factor *η* can be obtained as the gradient of a plot of ΔΔ*H*^‡^ against Δ*U*^‡^_EF_ (Fig. 4), and is linear (*R*^2^ = 0.9996), and shows a 71% conversion of Δ*U*^‡^_EF_ into ΔΔ*H*^‡^. This strong, linear catalytic response to the field indicates that acyl transfer catalysis in cage 1 will be strongly accelerated by positive EFs oriented along the *z*-axis. In contrast, applying EFs in various *x*–*y* planes shows a low field-dependency, with *η* ≈ 10% (Fig. S15). Overall, these results support the hypothesis that the studied cage-catalysed reaction should be suitable for electric field catalysis because: (i) the triptycene axis aligns well with the reaction axis, and (ii) the cage is highly resistant to mechanisms that could reduce EF catalysis, such as geometric distortions to oppose/screen *F*_app_ or reorientations that reduce the activation dipole Δ*µ*^‡^.

### Finite capacitor model for quantifying local EFs in organised cavities resembling cages 2–14

In order to discover whether the reaction barrier changes calculated in charged cage candidates 2–14 (Fig. 3) are also proportional to their internal (local) EFs, we set out to quantify the fields generated by the charged groups, A and B. These fields could be reconstructed from partial charges,^94^ but such an approach is not ideal as partial charges are not unambiguously defined. Instead, we sought to develop a simpler model, based on the rigorously defined dipole moment and geometry, to obtain estimates of the EF inside the cavity for any given A and B groups.

In previous work,^93^ external fields *F*_app_ are usually represented with a virtually infinite plate capacitor model:5
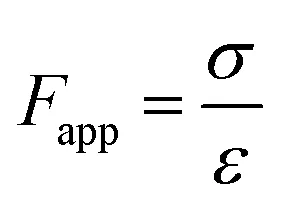
where *σ* is the charge density and *ε* the electric permittivity *ε*_0_*ε*_r_, and we set *ε*_r_ = 4.0.^92^ Here, since the charges are localised at sites A and B, we introduce a finite capacitor model (FCM) to estimate the electric field inside the cavity, which relies on the following assumptions:

(1) The local field produced by a substituent (*i.e. F*_A_, *F*_B_) behaves as if produced by a disk with radius *r* centered on the charged substituent atom. At distance *d* from the disk center along the reaction axis, the field is equal to:6
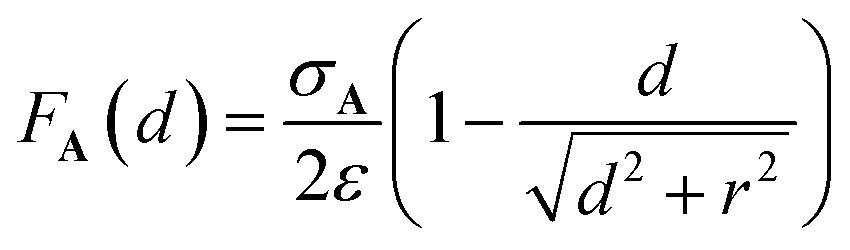


(2) We evaluate the substituent-produced field *F*_sub_ at the midpoint between A and B (center of the cage; *d*_A_ = *d*_B_ = ½*d*_AB_). For simplicity, we assume that each pair of substituents (*i.e.* each cage) can be represented by a single charge separation, *σ*_AB_, and that the charge is delocalised to the same extent in all cages (single value of *r*):7
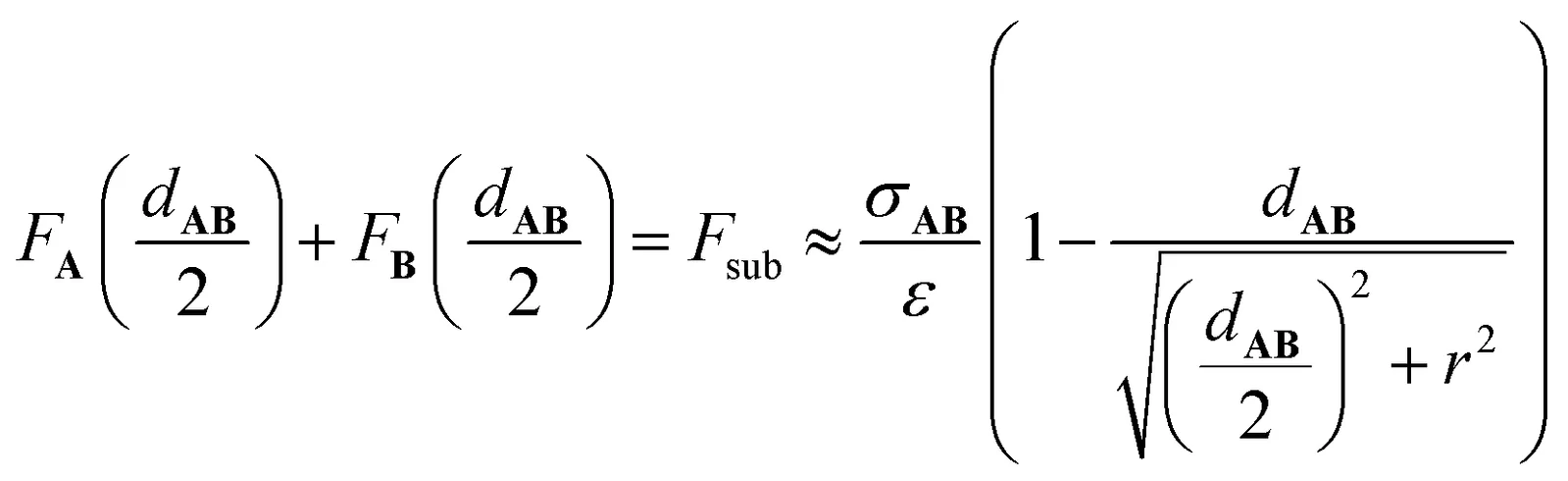


(3) Assuming that positive and negative charges *Q* are centered at the formally charged atoms of A and B, we can estimate the charge density *σ*_AB_ from the *z* component of the dipole moment of the empty substituted cage *µ*^*z*^_**AB**_:8
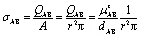
which gives the FCM equation:9
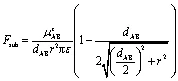


The FCM, eqn (9), agrees with the intuition that strong effective fields *F*_sub_ will be present in cages with: (i) a large dipole moment *µ*^*z*^_**AB**_; (ii) charges distributed over a small area *r*^2^π, and; (iii) a small distance *d*_AB_ between the substituents A and B. While *µ*^*z*^_**AB**_ and *d*_AB_ are straightforward to obtain by modelling (empty) cages using any electronic structure method, the choice of a value for disk radius *r* is less obvious. Electrostatic potential plots of the cages (Fig. S16–S18) indicate charge is approximately localised to the formally charged atom and its nearest neighbours. We therefore set *r* = 2.18 Å (∼1.5 the average length of bonds around the formally charged atoms). This value of *r* yields computed field strengths *F*_sub_ of ∼0.2 V Å^−1^ for the most active cages 2–5 (see Tables S3 and S4), only slightly lower than enzyme fields (*e.g.* cyclophilin A previously estimated at 0.5 V Å^−1^).^37,95^ These values, typical for ionic systems, are an order of magnitude smaller than ionising potentials or covalent bond strengths (∼3–5 eV Å^−1^; ∼100 kcal mol^−1^), but strong enough to lower activation barriers (∼0.6–1.1 eV = ∼14–25 kcal mol^−1^) by a substantial amount.

### Application of the finite capacitor model

We next estimated for cages 2–14 the local fields *F*_sub_ using the FCM (Fig. 5a, yellow) and compared the catalytic effect of these cages with catalysis in externally applied fields *F*_app_ (Fig. 5a, grey).

We find that catalysis is largely independent of the source of the electric field. This suggests that: (i) our value of *r* is chosen well and that the FCM produces reasonable estimates of *F*_sub_, and (ii) that barrier changes may be qualitatively predicted only from dipole moments and geometries, demonstrating that change in catalysis in these cages is a through-space effect. We next plotted the field–dipole interaction energy changes, Δ*U*^‡^_EF_ against ΔΔ*H*^‡^_**2–14**_ (Fig. 5b) to quantitatively determine to what extent the acyl transfer reaction barrier is dependent on the local EFs generated by the charged substituents, A and B. Fig. 5b shows two regimes: one for favourable fields (cages 1–11, yellow) and one for unfavourable fields (cages 12–14, grey). For the favourable fields, a very good linear correlation is found between the field–dipole interaction and the reaction barrier (*R*^2^ = 0.73), further confirming the strong EF effect on catalysis. Cages with unfavourable fields (12–14) deviate from this line, and are discussed in the next heading. The field-response factor *η* of 50 ± 5%, indicates very good conversion of the field into a reduced reaction barrier, with moderate attenuation (see text in SI, eqn (4)).

To demonstrate the possible use of the FCM in high-throughput screening, we approximately computed Δ*U*^‡^_**1–14**_ values using a single value of Δ*µ*^‡^_1_, taken from the unsubstituted cage 1 geometries, along with *F*_sub_ values from the FCM. In this case (Fig. 5c), we obtain idealised ∼1 : 1 correspondence (*η* = 1.03) between Δ*U*^‡^_EF_ and ΔΔ*H*^‡^, demonstrating a way of quantitatively estimating catalysis changes in substituted cages based only on the initial TS/RC pair, and many empty cage geometries. This result also implies that the partial attenuation of local EF effects on catalysis arises due to predictable changes in Δ*µ*^‡^_**2–11**_ in local fields *F*_sub_. In summary, we have validated our conceptual design: quantification of tuneable, measurable EFs in dipolar cavities. Experimentally, the catalytic motifs (*e.g.* carbonyl groups) or bound substrate can act as a reporter for the field strength using concepts from Stark spectroscopy (*i.e.* measuring field-dependent IR shifts).^96^

### Chemical insight and design rules

#### Unfavourable fields lead to reactant reorientation

It is worth examining why cages 12–14 do not show ΔΔ*H*^‡^ values that are the negative of their flipped counterparts (cages 7, 10, 5), and why they do not obey the correlation in Fig. 5b. Due to the open cavity of cage 1, the reactant complex is constrained only by a few hydrogen bonds (Fig. S2, <1 kcal mol^−1^).^31^ Strong, unfavourable fields therefore lead to facile reactant complex reorganisation (*e.g.* cages 12–14) that can alter Δ*µ*^‡^ and increase the catalytic barrier. Reactions in which the RC dipole is already approximately aligned with, but smaller than, the TS dipole are therefore the most straightforward to exploit. However, in general, optimal EF catalysis requires constraining the reactant complex relative to the TS, a design feature possible in supramolecular and enzyme cavities, but not in generic external EFs. Although enzyme catalysis is often rationalised in terms of geometric preorganisation or TS-specific binding, our results highlight an underappreciated requirement for optimal EF catalysis: the RC must be bound tightly enough to resist field-driven realignment, preserving the electrostatic tension that selectively stabilises the TS.

#### Favourable fields promote a late TS

The optimal field direction to promote acyl transfer catalysis in cage 1 is not immediately obvious from inspection of the cyclic proton-wire-like **TS^‡^_1_** (Fig. 6). Traditional organocatalytic design rules prompt the questions: how do the charged substituents, A and B, modulate the p*K*_a_ of the acid groups? Should the lower carboxylic acid be best activated as an acid to protonate the carbonyl electrophile (H^9^), or a base to deprotonate alcohol pronucleophile (H^3^)? Our analysis suggests neither seems to dominate the changing reaction barriers.

To gain chemical insight, we analysed bond lengths in **TS^‡^_1_** across cages 2–14, to assess how the local EF from the charged groups might alter the structure and position of the TS along the reaction coordinate (Fig. S23). The mass-weighted bond length changes (see SI, S2.11) for catalytically-proficient cage 2 relative to cage 1 (zero-field) are shown in Fig. 6, with negative values meaning a shorter bond in the favourable cage 2 TS. The data suggest a field-induced late TS in cages 2–5 characterised by advanced formation of the new O_4_–C_5_ bond (blue line, see also Fig. S28), despite a less-elongated carbonyl bond C_5_=O_8_ that is less protonated O_8_–H_9_ (red line, see also Fig. S25 and S28) and negligible lengthening (deprotonation) of the alcohol nucleophilic oxygen O_4_–H_3_ (Fig. S27). This analysis reveals a crucial insight: through-space electric field effects can dominate intuitive through-bond contributions to catalysis, such as electrophile protonation or nucleophile deprotonation. In this case, prediction of the favourable field direction for catalysis aligns with intuition associated with promoting HOMO (Nu, O_4_) → LUMO (E, C_5_) interactions.^10^ While limiting the rationale to frontier orbitals may not be appropriate because the EF affects many molecular orbitals (Fig. S29), this analysis highlights the opportunities in catalyst design outside of the multipoint interactions that dominate organocatalyst and bifunctional catalyst development.

#### Quantifying the catalytic activity due to the EF

Finally, we note that, for our example cage system, at *r* = 2.18 Å the best-performing cages 2–5 show a catalytic activity of around 13 kcal mol^−1^ per V Å^−1^, while the externally applied EF gives roughly 17 kcal mol^−1^ per V Å^−1^ (Fig. 5a and S21, S22), which respectively correspond to *η* ≈ 50% and *η* ≈ 71% conversion of Δ*U*^‡^_EF_ into ΔΔ*H*^‡^. While it may be difficult to increase *η*, we find that it does not decrease for the best-performing substituents, indicating no change of mechanism in stronger fields. This suggests that it may be possible to increase the catalytic activity even further, either by designing a cage with a larger Δ*µ*^‡^ or by identifying a cage with a larger *F*_sub_.

## Conclusions

In summary, we have proposed and computationally validated a dipolar cavity strategy for controlling EFs over solution-phase reactions. We exemplify our general design approach by modelling synthetically plausible charged analogues of known organocatalyst cage 1. Density-functional and coupled-cluster calculations indicate cavities of 1 nm hosting singly-charged substituents on their exterior can generate local fields comparable to enzyme active sites (∼0.2 V Å^−1^). Acyl transfer catalysis in the cavity of cage 1 is sensitive to the orientation and strength of the locally produced field, with barrier reductions of 13 kcal mol^−1^ per V Å^−1^, equivalent to a rate enhancement over the neutral cage of two orders of magnitude at the strongest investigated fields.

To rationalise the effect of the EF on catalysis, we developed a finite capacitor model for estimating cavity EF strengths from simple geometric and charge inputs. This model is general and transferable to any dipolar cavity satisfying our design approach, and we validate it against external field calculations. Such EF-based models may become important, since analysis of geometric perturbations of the transition states in local EFs in cage 1 analogues shows that through-space effects can dominate typical through-bond intuition in catalyst design.

Our results reinforce the value of supramolecular hosts as enzyme mimics, serving here as scaffolds capable of applying EFs independently of primary catalytic motifs. For instance, our observation that optimal catalysis required the substrate binding strength to exceed the electrostatic strain of the reactant complex invites new EF-based perspectives on “ground-state destabilisation” in enzymes, and suggests strong substrate binding may exist to enable optimal EF catalysis. Progress now requires experimental work to determine accessible structures and to what extent solvent, entropic, and counterion effects complicate implementation. Thus, our theoretical study sets the stage for a new generation of catalysts that exploit designed local EFs to bridge the gap between enzyme efficiency and synthetic applicability.

## Methods

All geometry optimisations were performed in the gas phase using Gaussian 16.^97^ Zero-point energy (ZPE) corrections were obtained using GoodVibes,^98^ and all reported thermochemical values include these corrections. Based on previous kinetic studies,^31^ a unimolecular rate-determining step was assumed (reactant complex → transition structure), so no corrections for change in standard state were necessary. All structures used in the analysis were confirmed to be minima or first-order saddle points through harmonic frequency calculations. For DFT calculations, as previously reported for this system,^31^ the PBE0 hybrid functional^99^ was used in conjunction with Ahlrichs' def2-SVP basis set^100^ and a damped D3 dispersion correction (D3BJ).^101,102^ Solvent effects (CHCl_3_) were modelled using the SMD implicit solvation model,^103^ and included as a single-point correction to Δ*H*; for best- and worst-performing cages, similar results were obtained (Fig. S7) by performing geometry optimisations and frequency calculations with an implicit solvent (C-PCM^104,105^). Explicit electric field calculations were performed by applying static fields, *F*_app_, along the reaction axis. In these calculations, two bridgehead atoms of the triptycene framework were frozen to prevent rigid-body reorientation in the field. High-level single-point energies were calculated using DLPNO-CCSD(T)^106^ as implemented in ORCA 5.0.3,^107,108^ employing the cc-pVTZ basis set.^109^ Solvent effects in these correlated calculations were included *via* the conductor-like polarisable continuum model (C-PCM),^104,105^ with chloroform as the solvent. Electrostatic potential (ESP) maps were generated using the orca_vpot utility in ORCA 5.0.3,^107,108^ based on electron densities computed with the PBE0 functional.^99^

## Author contributions

KGA and IR conceived and designed the study. IR developed the theoretical framework and equations. MV, KGA and IR performed the computational investigations, data curation, formal analysis, validation, and visualisation. KGA and IR developed the methodology, supervised the project, administered the project, and provided resources. KGA and IR wrote the original draft, and all authors contributed to review and editing of the manuscript.

## Conflicts of interest

There are no conflicts to declare.

## Supplementary Material

SC-OLF-D6SC04940K-s001

## Data Availability

Cartesian coordinates are available on Zenodo https://doi.org/10.5281/zenodo.15148998. Supplementary information (SI): computational details, all geometry files, additional graphs and analysis, and additional reference annotations. See DOI: https://doi.org/10.1039/d6sc04940k.
