# Supplementary material for: Towards next generation enzyme mimics through local electric field control in organic cage catalysts
Source: Chem Sci. 2026 Oct 2. Online ahead of print. doi: 10.1039/d6sc04940k (PMC13631960; doi:10.1039/d6sc04940k)
Supplement: Supplementary file 1 [file SC-OLF-D6SC04940K-s001.pdf]

Supplementary Information (SI) for Chemical Science.
This journal is © The Royal Society of Chemistry

Supplementary Information: Towards Next Generation Enzyme Mimics Through Local Electric Field Control in Organic Cage Catalysts

Marco Vitek [1], Igor Rončević* [2], and Keith G. Andrews* [3]

[1]Institute of Organic Chemistry and Biochemistry of the Czech Academy of Sciences, Flemingovo nám. 2, 16610 Prague, Czech Republic
[2]Department of Chemistry, University of Manchester, Oxford Road, Manchester, M13 9PL, UK
[3]Department of Chemistry, Durham University, Lower Mount Joy, South Rd, Durham, DH1 3LE, UK
*Electronic mail: igor.roncevic@manchester.ac.uk, keith.g.andrews@durham.ac.uk

August 24, 2026

Contents

S1 Computational Methods 2

S2 Supplementary Results and Discussion 3

S2.1 Cage 1 Overview and Names . . . 3
S2.2 Reaction Overview . . . 4
S2.3 Through Space Effects in Triptycenes . . . 6
S2.4 Molecular Dynamics Analysis of Cage 1 . . . 7
S2.5 Gas-Phase, Solvation, and Structural Effects on Activation Enthalpy Differences . . . 8
S2.6 Effect of the Substituents on Equilibria and Rate-Determining Steps . . . 10
S2.7 Counterions and Charge Screening . . . 12
S2.8 External Field Effects . . . 13
S2.9 Electrostatic Potential Maps . . . 15
S2.10 Finite Capacitor Model Validation . . . 17
S2.11 Numerical data . . . 19
S2.12 Geometrical Analysis of the TS Complex . . . 21
S2.13 Orbital Energy Trends under External Electric Fields . . . 25

References 26

Main Text Reference Annotations 26

S1 Computational Methods

Unless specified otherwise, all geometry optimisations were performed in the gas phase using GAUSSIAN 16 [1]. Zero-point energy (ZPE) corrections were obtained using `GoodVibes` [2], and all reported thermochemical values include these corrections. All structures used in the analysis were confirmed to be minima or first-order saddle points through harmonic frequency calculations.

For DFT calculations, as previously reported for this system [3], the PBE0 hybrid functional [4] was used in conjunction with Ahlrichs' def2-SVP basis set [5] and a damped D3 dispersion correction (D3BJ) [6, 7]. Solvent effects ($CHCl_3$, THF, toluene) were modelled using the SMD implicit solvation model [8] and were included only in single-point energy calculations on gas phase optimised structures. In addition, a selected subset of cage structures was optimised in $CHCl_3$ using the conductor-like polarisable continuum model (CPCM) at the same PBE0-D3BJ/def2-SVP level of theory.

Explicit electric field calculations were performed by applying static fields, F_{app}, along the reaction axis. In these calculations, the coordinates for two bridgehead atoms of the triptycene framework were frozen to prevent rigid-body reorientation in the field.

Following Stuyver [9], a constant field, F_{app}, can be produced by a parallel plate capacitor characterised by the charge density, σ:

$$F_{\text{app}} = \frac{\sigma}{\varepsilon} \tag{S1}$$

where ε is the electric permittivity $\varepsilon_0 \cdot \varepsilon_r$ with ε_r set to 4.0 [10], a value commonly used for enzyme models and appropriate here due to the largely hydrocarbon-based framework of the cage. Additionally, the solvent, chloroform, has a similar value, $\varepsilon_r = 4.8$, and is unable to significantly solvate the reaction along the triptycene-triptycene axis in any case.

High-level single-point energies were calculated using DLPNO-CCSD(T) [11] as implemented in ORCA 5.0.3 [12], employing the cc-pVTZ basis set [13]. Solvent effects in these correlated calculations were included via the CPCM model [14], with chloroform as the solvent.

Electrostatic potential (ESP) maps were generated using the `orca_vpot` utility in ORCA 5.0.3 [12], based on electron densities computed with the PBE0 functional [4].

Cartesian coordinates are available on Zenodo at https://doi.org/10.5281/zenodo.15148998.

S2 Supplementary Results and Discussion

S2.1 Cage 1 Overview and Names

Figure S1 shows various structural views, names and identifiers to help readers visualise the complex 3D structure of cage **1**. No IUPAC name could be generated using any software available to the authors.

depictions of the triptycene fragment used to make the cage

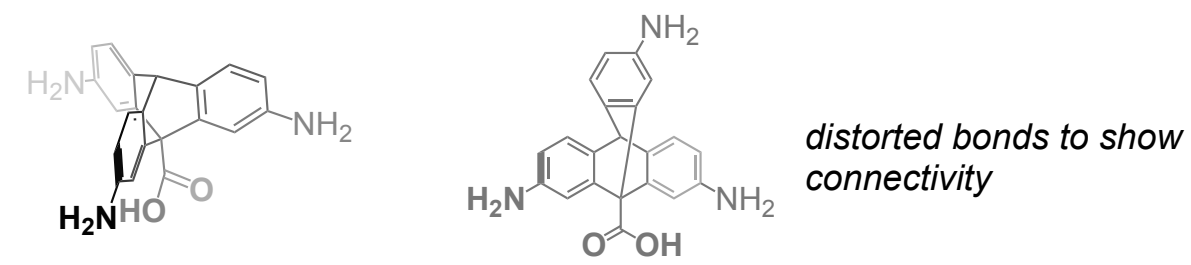

NC(C=C1)=CC2=C1C3C4=CC=C(N)C=C4C2(C(O)=O)C5=CC(N)=CC=C53

depictions of cage 1

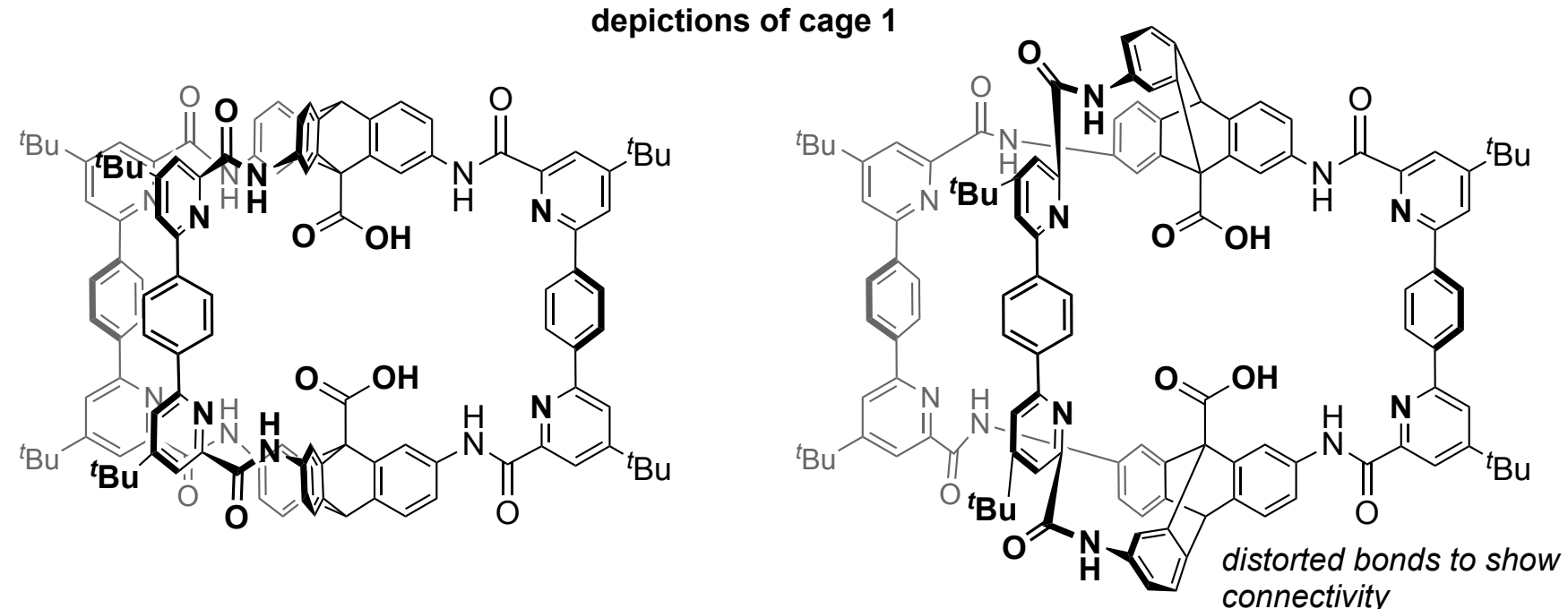

SMILES= O=C(C1=CC(C(C)(C)C)=CC(C2=CC=C(C=C2)C3=CC(C(C)(C)C)=CC4=N3)=N1)NC5=CC=C(C6C7=C8C=C(NC(C9=CC(C(C)(C)C)=CC(C%10=CC=C(C=C%10)C%11=CC(C(C)(C)C)=CC(C(NC(C=C%12)=CC%13=C%12C%14C%15=CC=C(C=C%15C%13(C(O)=O)C%16=CC(NC%17=O)=CC=C%16%14)NC4=O)=O)=N%11)=N9)=O)C=C7)C(C8(C(O)=O)C%18=CC(NC(C%19=CC(C(C)(C)C)=CC(C%20=CC=C(C%21=CC(C(C)(C)C)=CC%17=N%21)C=C%20)=N%19)=O)=CC=C%186)=C5

InChIKey= OEASIOBUUCTGRA-UHFFFAOYSA-N

InChI= 1S/C120H106N12O10/c1-113(2,3)67-43-91-61-19-21-62(22-20-61)92-44-68(114(4,5)6)50-98(128-92)106(134)122-76-34-40-82-88(58-76)120(112(141)142)89-59-77-35-41-83(89)104(82)84-42-36-78(60-90(84)120)126-110(138)102-54-72(118(16,17)18)48-96(132-102)66-29-25-64(26-30-66)94-46-70(116(10,11)12)52-100(130-94)108(136)124-75-33-39-81-87(57-75)119(111(139)140)85-55-73(121-105(133)97(49-67)127-91)31-37-79(85)103(81)80-38-32-74(56-86(80)119)123-107(135)99-51-69(115(7,8)9)45-93(129-99)63-23-27-65(28-24-63)95-47-71(117(13,14)15)53-101(131-95)109(137)125-77/h19-60,103-104H,1-18H3,(H,121,133)(H,122,134)(H,123,135)(H,124,136)(H,125,137)(H,126,138)(H,139,140)(H,141,142)

Figure S1: Names and identifiers for the cage **1** structure used in this study.

S2.2 Reaction Overview

The acyl transfer reaction in cage **1** has previously been studied both experimentally and computationally [3]. From this analysis, the mechanism has been described in good detail, and kinetic parameters were determined (Figure S2). For the purposes of this study, we assume that rapid, reversible anhydride exchange will result in an equilibrating population of **1Ac**$_1$ able to undergo the rate-determining acyl transfer step, which will control the overall catalysis rate.

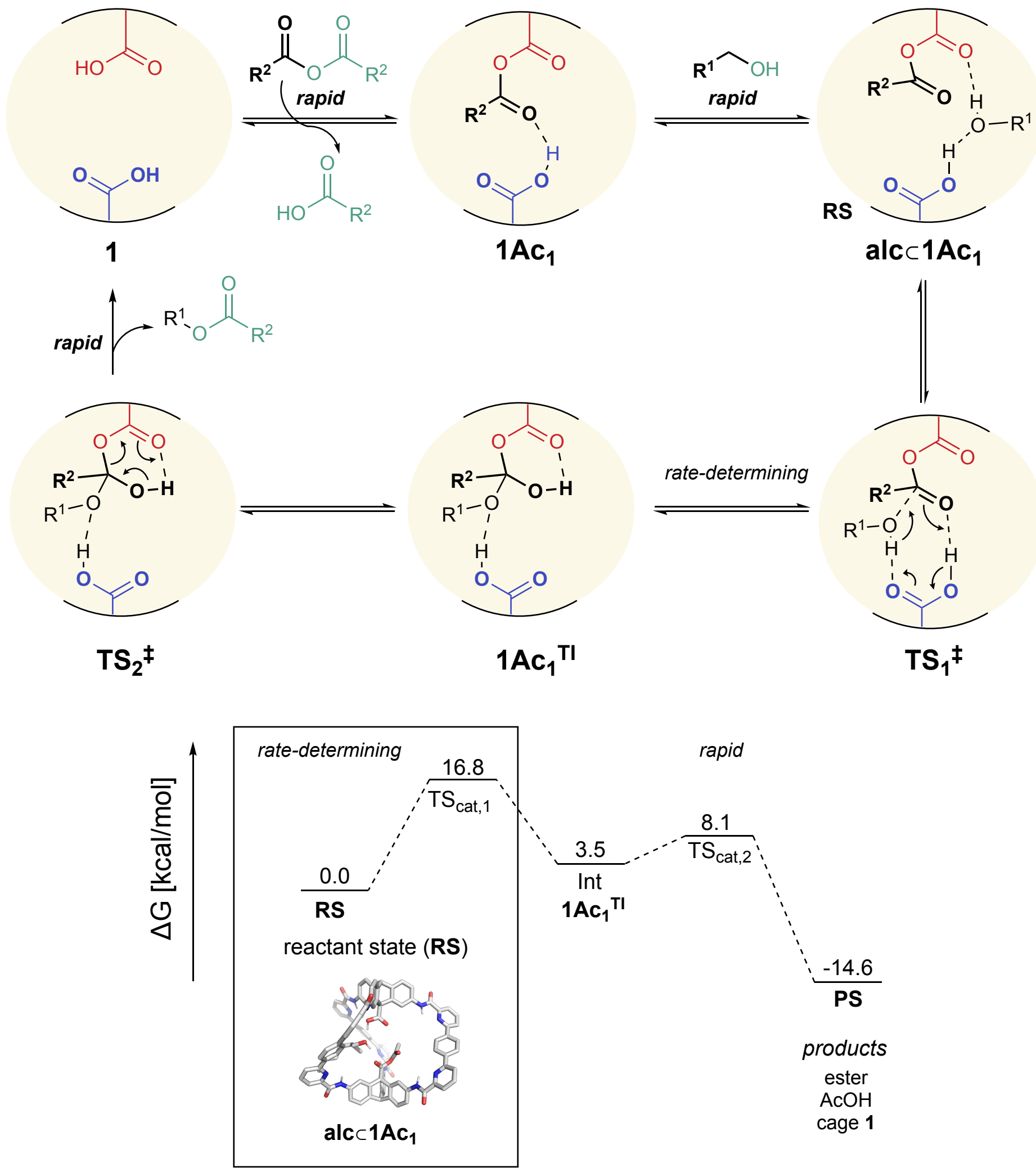

Figure S2: Mechanism for the cage **1**-promoted acyl transfer reaction. Experimentally, acylation has been shown to be rapid, and the alcohol binding occurs in fast exchange. The implicit tetrahedral intermediate (TI) is too short lived to be observed. The rate-determining step is, by a comfortable energetic margin, the acyl transfer from **alc**$\subset$**1Ac**$_1$ via **TS**$_1^\ddagger$ to form **1Ac**$_1^{\mathbf{TI}}$. The reported reaction is performed in dry, neutral $CDCl_3$ (ethanol free).

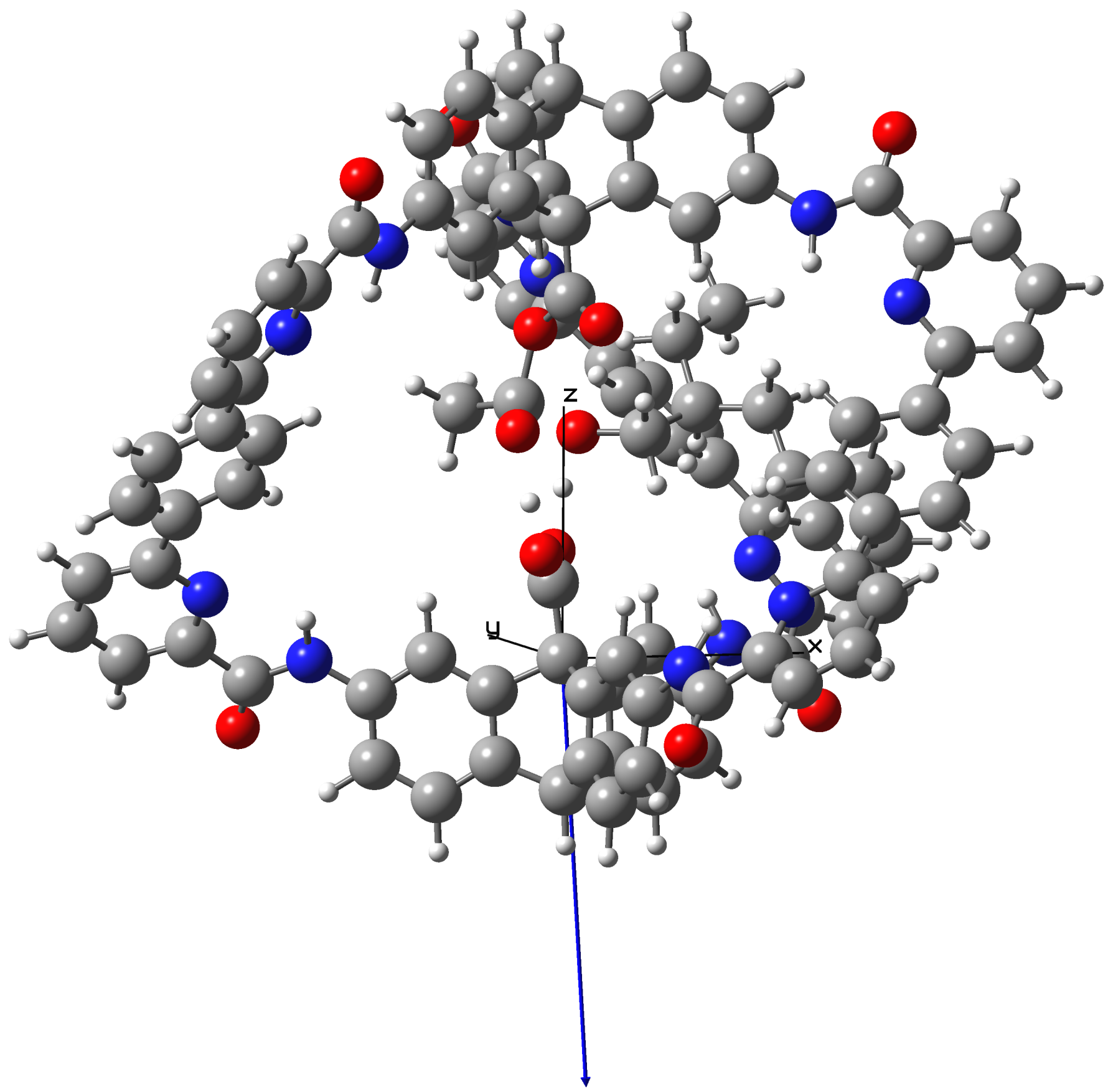

Figure S3: Optimised transition state structure of cage **1** with the cartesian coordinate axes (black lines) indicating the *x*, *y*, and *z* directions. The blue arrow represents the direction of the dipole moment $\vec{\mu}_{\mathrm{TS}}$, which is oriented predominantly along the *z*-axis and aligns with the reaction coordinate. Note that Gaussian displays the dipole vector arrow as negative to positive. The structure shown corresponds to calculations under an applied external electric field of 0.35 V/Å in the positive *z*-direction.

S2.3 Through Space Effects in Triptycenes

We provide a brief discussion of our statement: "The A-B-substituted cores of the triptycene units of cage **1** resemble bridgehead-substituted bicyclo[2.2.2]octane-1-carboxylic acids, which are known to modulate the acid p*K*a through-space (i.e. field effects) rather than through 6–8 sigma bonds."

The central polycycle in a triptycene is a bicyclo[2.2.2]octane core. The bridgehead substituents on the cages are not like para-substituents on an aromatic ring – there is no possible π-resonance pathway. Instead, there are 6–8 sigma bonds (depending on whether the charged atom is at the bridgehead, or attached to the bridgehead) between the ion centre and the reactive groups (e.g. proton or anhydride) at the carboxylic acid.

Substituents on bicyclo[2.2.2]octane-1-carboxylic acids are well known since the 1960s to affect the pK_a of the acid by field effects, on the basis of ingenious bond deletion experiments (main text refs 90–91, especially *J. Am. Chem. Soc.* **1968**, 90, 2, 336-341) [15, 16]. Briefly, deleting a carbon atom gives a bicyclo[2.2.1]heptane-1-carboxylic acid. This deletion alters the through-bond electronic communication differently to the through-space (field) effect. The predicted effect difference was calculated by Wilcox and Leung according to existing models for inductive and field effects. When the pK_a values of a series of octane/heptane acids were experimentally measured, their ratios matched precisely with the field model, and was 20 standard deviations from matching the inductive model. More intuitively, chemists rarely invoke electronic through-bond effects across 6–8 sigma bonds.

Since triptycene carboxylic acids are geometrically analogous to bicyclo[2.2.2]octane-1-carboxylic acids, the same field pK_a effects are expected, and it is known that carbonium ions and radicals at triptycene bridgehead positions are not stabilised by the presence of the aromatic rings (i.e. demonstrating that moving from bicyclooctane to triptycene is unlikely to invoke a response from the new π-system (*J. Am. Chem. Soc.* **1954**, 76, 4, 1088–1096) [17].

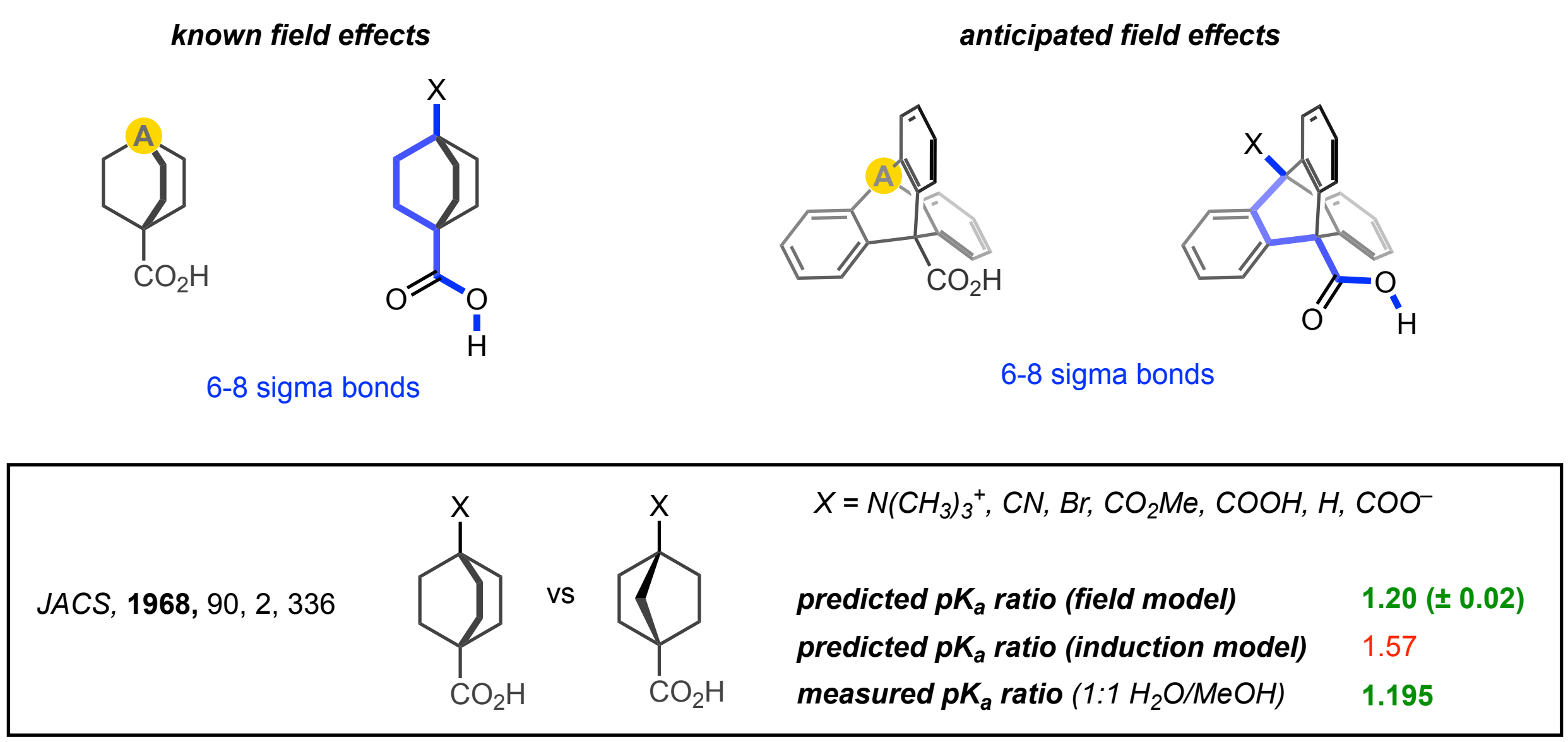

Figure S4: Summary of reported experimental work demonstrating the through-space (field) effects for substituents on bicyclo[2.2.2]octane-1-carboxylic acids , and the relevance for triptycenes.

S2.4 Molecular Dynamics Analysis of Cage 1

To support the use of static electric field models and the assumptions underlying the finite capacitor framework, we summarise previously reported molecular dynamics (MD) simulations of cage **1** in chloroform [3]. These simulations demonstrate that the A/B-substituted bridgehead positions remain nearly collinear throughout the trajectory, ensuring that the field defined between them consistently encompasses the catalytic site. The reported angle and distance distributions validate the structural rigidity of the cage under ambient conditions and justify the use of fixed field orientations in our calculations.

According to the reported method [3], molecular dynamics (MD) simulations were performed using the GROMACS 2021.3 program [18, 19]. Cage **1** was placed into a 5 nm × 5 nm × 5 nm cubic box with 500 chloroform molecules. The systems were energy-minimised. They were equilibrated in an NVT ensemble using a v-rescale thermostat (298 K; τ=0.1 ps) for 20 ps and an NPT ensemble using a c-rescale barostat (1 atm; τ=2 ps) for 20 ps [20]. Production runs were performed with v-rescale thermostats and c-rescale barostats [21]. All calculations were performed using a 2 fs time step. 1 μs of data were analysed, and distances and angles extracted using the GROMACS *distance* and *angle* functions. The average distance between the two carboxylic acid carbon atoms was 6.77 Å with a standard deviation of 0.29 Å (4% deviation), demonstrating a good balance between rigidity and flexibility. The two bridgehead positions, A and B, in cage **1** have an average distance of 1.51 nm with a standard deviation of 0.28 Å (2% deviation). The deviation from parallel alignment of the two triptycene groups in cage **1** was approximated by measuring the deviation from 0° in each of the angles, θ_1, θ_2, where θ_1 = ⟨ijk and θ_2= ⟨jkl, where i is the outer bridgehead position A; j is the closest carboxylic acid carbon; k is the opposite carboxylic acid carbon; and l is the bridgehead position B. The angle distribution over 1 μs has a maximum at only 5.9°, a deviation of 3% from collinearity, with 96% of the ensemble at less than 11° deviation from collinearity (Figure S5).

These measurements from the MD simulations support the hypothesis that the field defined between bridgehead positions, A and B, will completely and permanently encompass the transition states between the two carboxylic acid groups in cage **1**, even as the cage explores available conformations in solution. These deviations from linearity are also well within the distance chosen for the finite capacitor plate diameter.

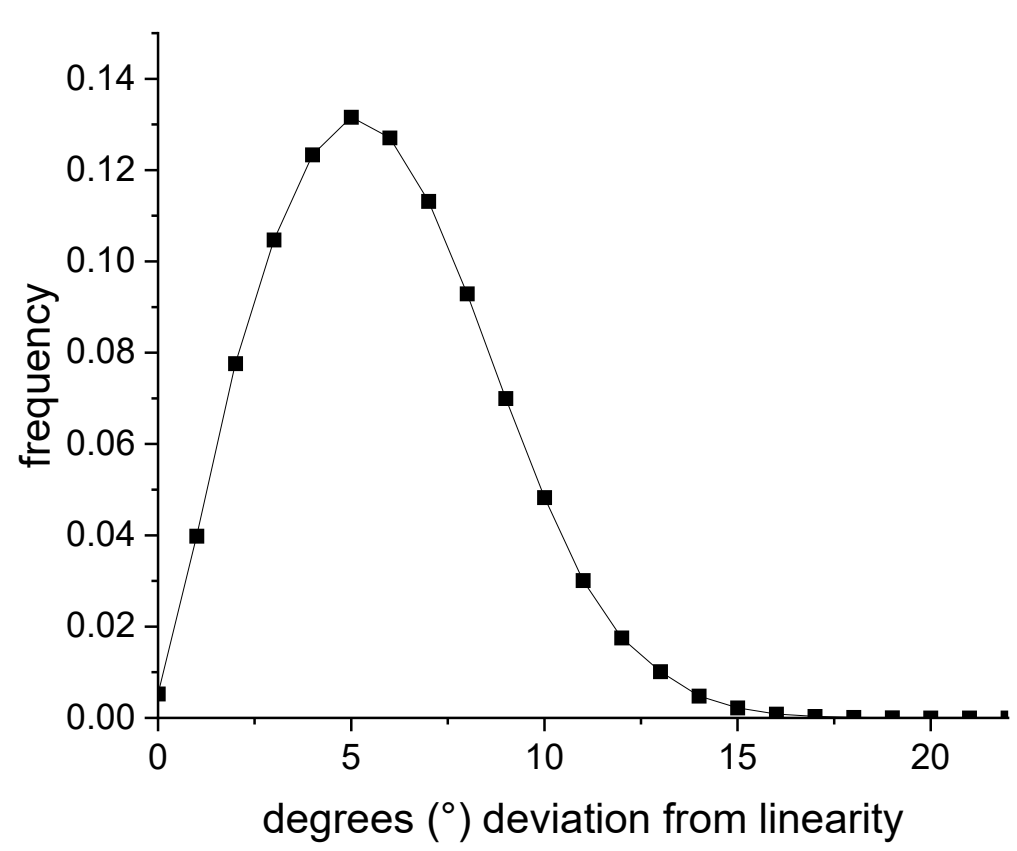

Figure S5: Ensemble deviation from linear, parallel alignment of the two triptycene groups in cage **1**, approximated as the angle between bridgehead position A or B and the two carboxylic acid carbons, measured over 1 μs using molecular dynamics simulations in chloroform at 298 K.

S2.5 Gas-Phase, Solvation, and Structural Effects on Activation Enthalpy Differences

To assess the influence of vibrational corrections on catalytic barrier predictions, we evaluated $\Delta\Delta H^\ddagger$ values for cages **2**–**14** based on single-point electronic energies from PBE0-D3BJ/SMD and DLPNO-CCSD(T)/CPCM calculations in chloroform, without including zero-point energy (ZPE) or thermal corrections. The resulting data are shown in Figure S6.

Compared to the ZPE-corrected values presented in Figure 5 of the main text, the uncorrected barriers exhibit systematically larger magnitudes—i.e., both catalysis and inhibition appear more pronounced. This indicates that ZPE corrections, particularly from low-frequency modes in the transition state, serve to moderate the purely electronic contributions to the activation enthalpy. Nevertheless, the qualitative trends and substituent ranking remain mostly unchanged, confirming that while electronic structure effects dominate the field response, vibrational contributions—especially from soft modes—play a non-negligible moderating role.

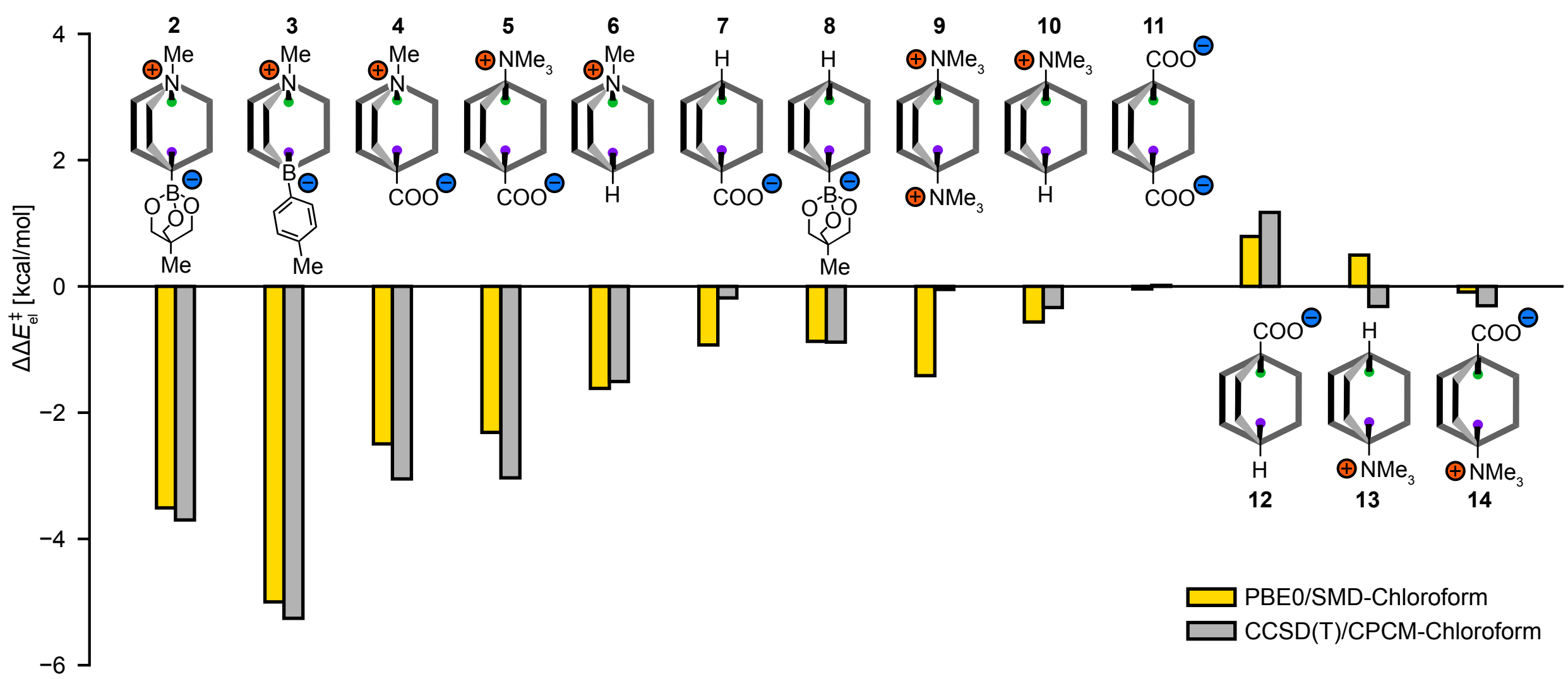

Figure S6: Activation energy differences $\Delta\Delta E^\ddagger_{\mathrm{el}}$ for cages **2**–**14** computed without including ZPE corrections, using PBE0-D3BJ/SMD($CHCl_3$) (yellow) and DLPNO-CCSD(T)/CPCM($CHCl_3$) (grey) in chloroform.

We investigated whether implicit solvation during geometry optimisations affects the computed activation barriers. Although chloroform has a relatively low dielectric constant, inclusion of solvent effects could, in principle, capture additional reorganisation contributions. Geometry optimisations and frequency calculations with C-PCM solvation (PBE0-D3BJ) were therefore performed for the reference cage **1**, best-performing cages **2**, **3** and **4**, and worst-performing cages **13** and **14**.

The results (Figure S7) show that: (i) the qualitative trends are unchanged, with cages **2**, **3** and **4** substantially lowering the barrier and cages **13** and **14** raising it relative to cage **1**; and (ii) solvation does not significantly screen the electronic field effects. Instead, slightly more negative $\Delta\Delta H^\ddagger$ values (by 0.5–1.5 kcal/mol) were obtained for all substituted cages relative to gas-phase geometries with single-point solvation corrections. In the case of cage **3**, small spurious additional imaginary frequencies were found at the TS; these were addressed by applying the quasiharmonic approximation to both the reactant complex and TS.

These findings highlight the challenges of obtaining fully converged results for such large, flexible systems when including implicit solvation throughout the optimisation. As the overall conclusions remain unaffected, we present gas-phase optimisations in the main text and provide these solvent-inclusive results here for completeness.

We also investigated a set of additional cages **15**–**18**, in which the substituents were inverted relative to their orientations in the original cages. The resulting $\Delta\Delta H^\ddagger$ values are shown in Figure S8.

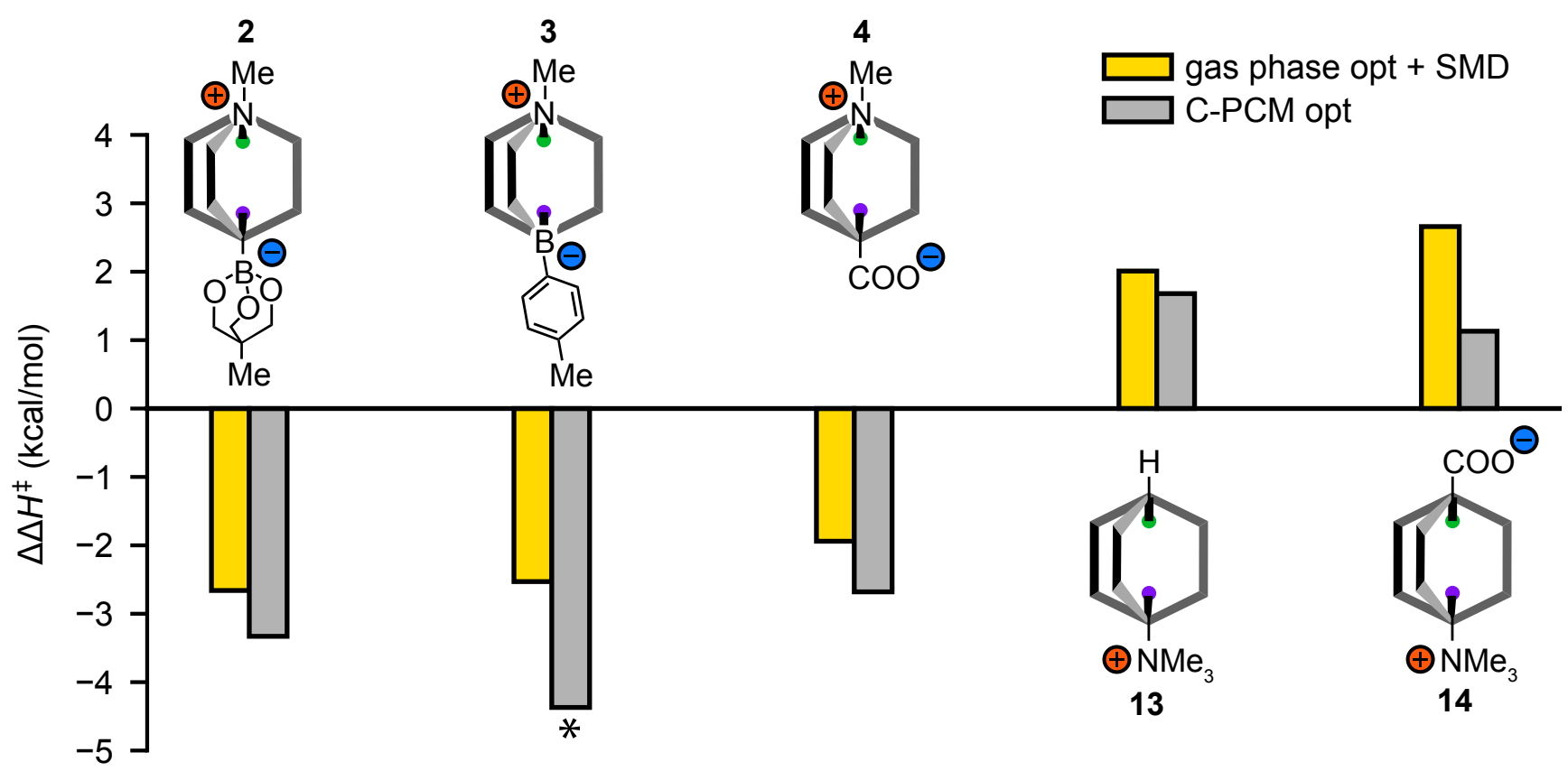

Figure S7: Comparison of activation enthalpy differences $\Delta\Delta H^{\ddagger}$ (including ZPE corrections; * indicates cases where the quasiharmonic approximation was applied to remove small spurious imaginary modes) for cages **2**, **3**, **4**, **13**, and **14**. Barriers were obtained either from gas-phase geometries with solvent effects ($CHCl_3$) included via SMD single-point calculations (yellow) or from full geometry optimisations with CPCM solvation in chloroform (grey). Values are given relative to cage **1**.

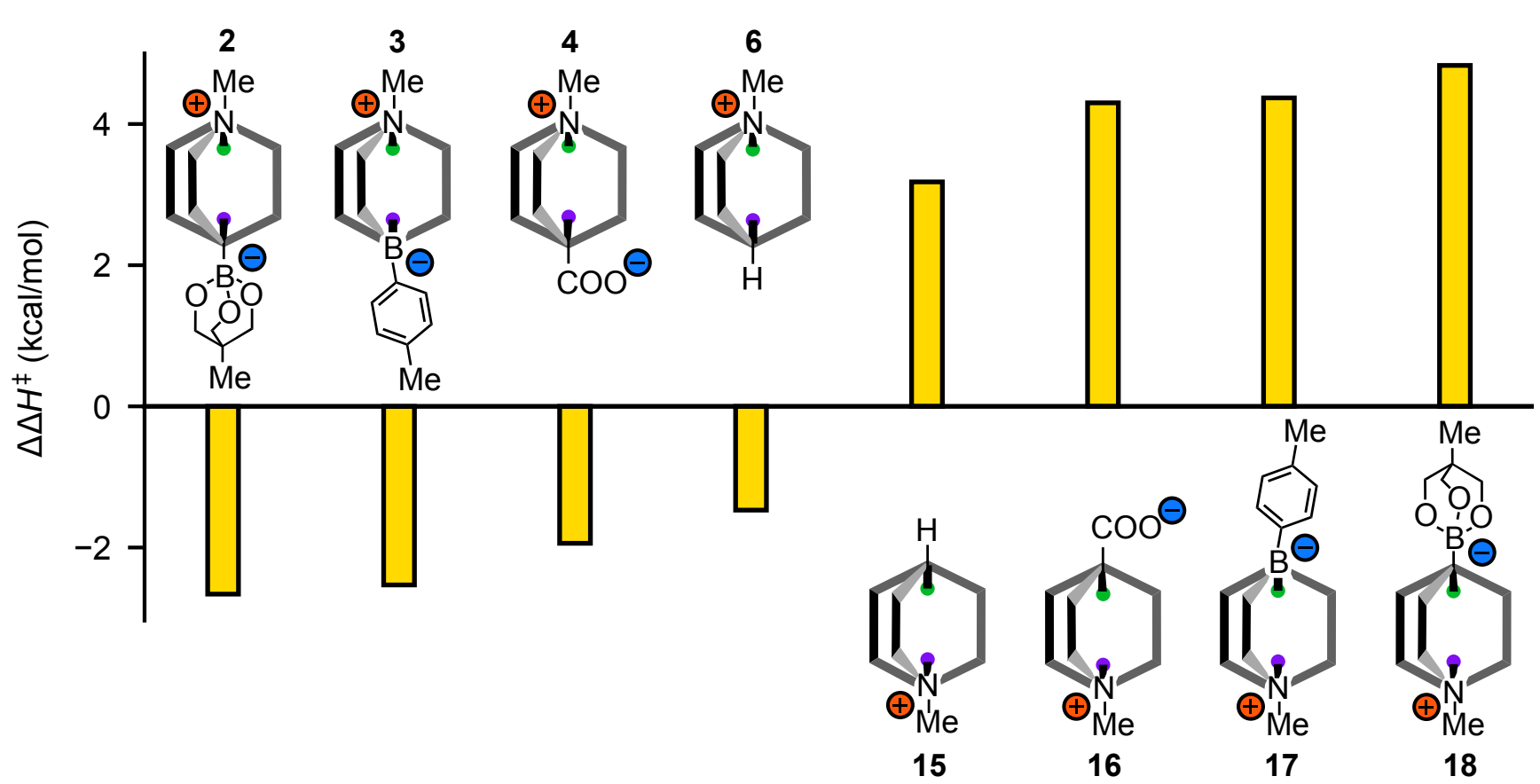

Figure S8: Activation enthalpy differences $\Delta\Delta H^{\ddagger}$ computed using PBE0-D3BJ/SMD($CHCl_3$) and including ZPE corrections for cages **2**, **3**, **4**, **6**, and **15–18**, the latter corresponding to structures with inverted substituent orientations. Values are referenced to cage **1**.

S2.6 Effect of the Substituents on Equilibria and Rate-Determining Steps

To determine whether the introduction of the charged substituents alters the rate-determining step, the key intermediates and transition states were recalculated for cage **2** and compared with the parent cage **1**. In both cases, $TS_1^{\ddagger}$ remains higher in energy than $TS_2^{\ddagger}$, indicating that the same step remains rate-determining after functionalisation (Figure S9).

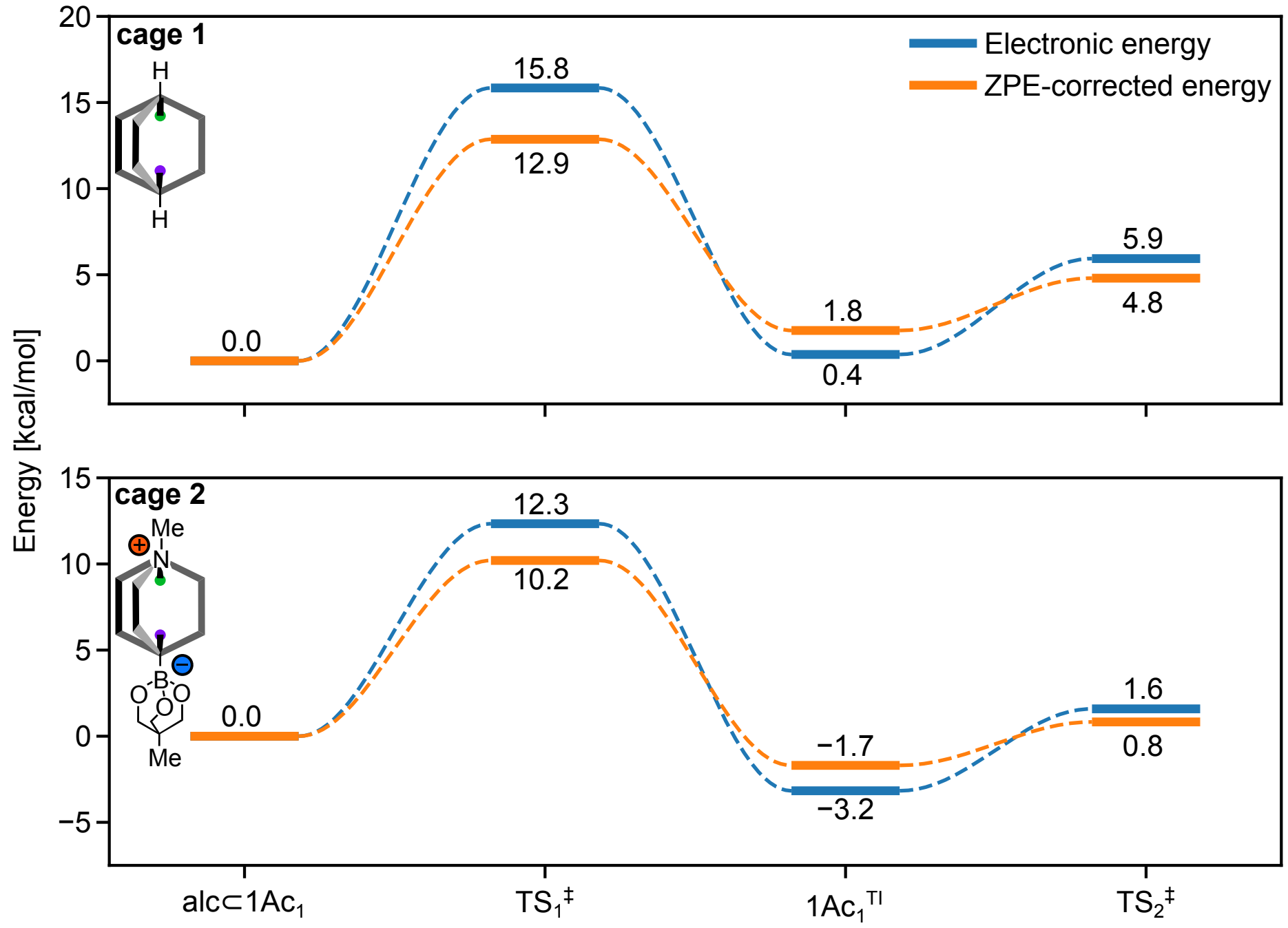

Figure S9: Relative electronic energies (blue) and zero-point-energy (ZPE)-corrected energies (orange) of the reactant state alc⊂$1Ac_1$, $TS_1^{\ddagger}$, $1Ac_1^{TI}$, and $TS_2^{\ddagger}$ for cages **1** and **2**, calculated at the PBE0-D3BJ/SMD($CHCl_3$) level. For each cage, energies are reported in kcal/mol relative to the corresponding alc⊂$1Ac_1$, set to 0.0 kcal/mol. In both cages, $TS_1^{\ddagger}$ lies higher in energy than $TS_2^{\ddagger}$, consistent with the step associated with $TS_1^{\ddagger}$ remaining rate-determining for cage **2**.

To assess the robustness of the calculated substituent effect, the activation enthalpy difference between cages **2** and **1** was evaluated in THF, $CHCl_3$, and toluene, with the same trend observed across all three solvents (Figure S10).

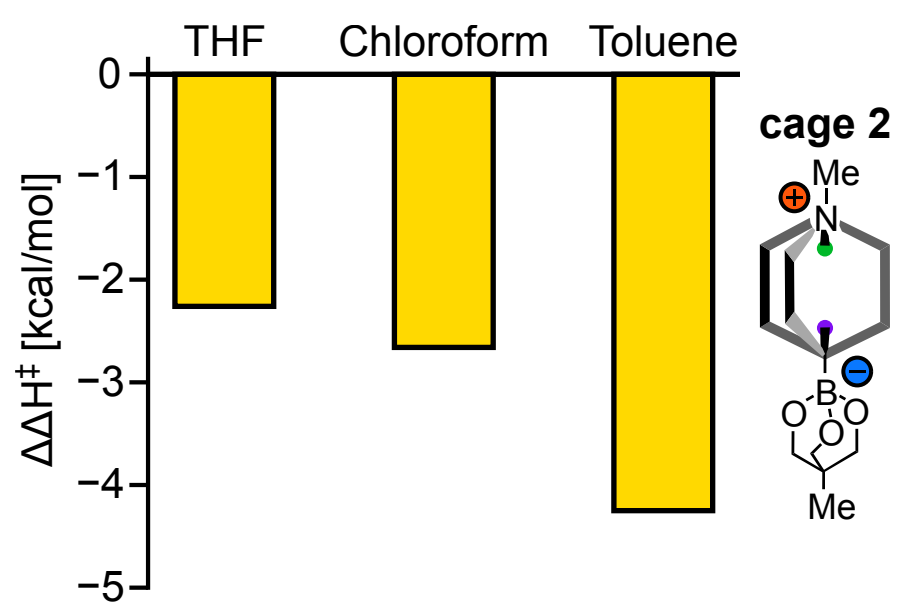

Figure S10: Activation enthalpy differences, $\Delta\Delta H^{\ddagger}$, for cage **2** relative to cage **1** in three solvents (THF, $CHCl_3$, and toluene; ε = 7.42, 4.71, 2.37), calculated using PBE0-D3BJ/SMD and including zero-point energy (ZPE) corrections.

To assess whether the orientation of the charged substituents alters the pre-equilibrium formation of the monoacetylated cage, ZPE-corrected reaction energies were calculated for the balanced reaction

$$\mathbf{X} + \mathrm{Ac_2O} \rightleftharpoons \mathbf{XAc1} + \mathrm{AcOH},$$

where $\mathbf{X} = \mathbf{1}$, **5**, or **14**. Alcohol binding was omitted from this comparison to isolate the thermodynamics of cage acetylation. The calculated reaction energies are small for all three cages (-1.84 to $+1.69$ kcal/mol), indicating that monoacetylation remains approximately thermoneutral upon introduction and reversal of the charged substituents. In particular, cages **1** and **5** give essentially identical reaction energies (a difference of $+0.06$ kcal/mol), while monoacetylation of cage **14** is modestly favoured, (Table S1). In practice, this pre-equilibrium does not affect the proposed experimental quantification of electric field catalysis for the acyl transfer step, since acyl transfer can be measured independently of the cage acylation by preformation of the acylated cage intermediate.

Table S1: ZPE-corrected reaction energies for monoacetylation of cages **1**, **5**, and **14**, calculated according to $\mathbf{X} + \mathrm{Ac_2O} \rightarrow \mathbf{XAc1} + \mathrm{AcOH}$ where $\mathbf{X} = \mathbf{1}$, **5**, or **14**.

Cage	$E_{\mathrm{ZPE}}(\mathbf{X} + \mathrm{Ac_2O})$ (a.u.)	$E_{\mathrm{ZPE}}(\mathbf{XAc1} + \mathrm{AcOH})$ (a.u.)	$\Delta E^{\mathrm{ZPE}}_{\mathrm{acet}}$ (kcal/mol)
**1**	-5863.1338	-5863.1312	1.63
**5**	-6224.1139	-6224.1112	1.69
**14**	-6224.1122	-6224.1151	-1.84

S2.7 Counterions and Charge Screening

We also performed a brief literature survey to probe the likely effect of a large non-coordinating counterion on the local EF screening. Specifically, we identified the popular metal-organic coordination cage catalysts reported by Lusby, which are Pd(II)-based lantern type cages with BArF counterions unable to approach the cavity, typically deployed in chlorinated solvent as used in this study. To estimate the distance between the counterion and the charged atom, we located representative crystal structures of cages with BArF counterions. Shown are the distances between the formally charged B and Pd atoms in the unit cell for three structures (each with two counterions), with their Cambridge Crystallographic Database Identifier and Deposition Number.

IGUZUI 1978675 [22] B. . . Pd distances = 7.94 Å, 12.40 Å

FEGJOU 2157970 [23] B. . . Pd distances = 8.90 Å, 16.6 Å

FEGJAG 2157966 [23] B. . . Pd distances = 8.80 Å, 9.0 Å

The X-ray data show that the charged cage metal ions are typically ∼9 Å or more away from the BArF ion. The majority of this steric protection comes from BArF, since the cage palladium atoms are sterically unhindered.

For the organic cages studied in this manuscript, the distance from the charged bridgehead ions to reactive groups inside the cavity covers the range 4.8–8.8 Å – i.e. most of the reactive site is much closer than the BArF counterion can get. An estimate using Coulomb's law shows that at distances, d, of 9–16 Å, the counterion will have a small effect, screening only 5–15% of the EF measured inside the cavity, 5 Å from the charged atom [proportional to $1/(5+d)^2$ and approximating as point charges]. This result matches very well with the calculated screening of the smaller ions (see point a, above).

Notably, the ion-to-ion distance in the zwitterionic organic cages studied in this manuscript (i.e. with plus/minus charged groups at opposite poles, e.g. cage **2**) is ∼14 Å, which is small enough that counterion pairs to these plus/minus cage ions may prefer to pair with each other (and possibly precipitate) rather than poorly solvate the cage ions.

We additionally estimated the counterion influence by placing counterions (Na^+/K^+ Cl^-/I^-) at different distances from the cage, and recomputing single-point energies (Fig. S11). These distances are reasonable (see discussion above), and they represent different amounts of screening of the bare ion Coulombic potential.

Our results (Fig. S11) show that bare counterions attenuate the catalysis of the best-performing cage **2** by only 5–20% (0.2–0.6 kcal/mol), and therefore do not alter the conclusions of the study.

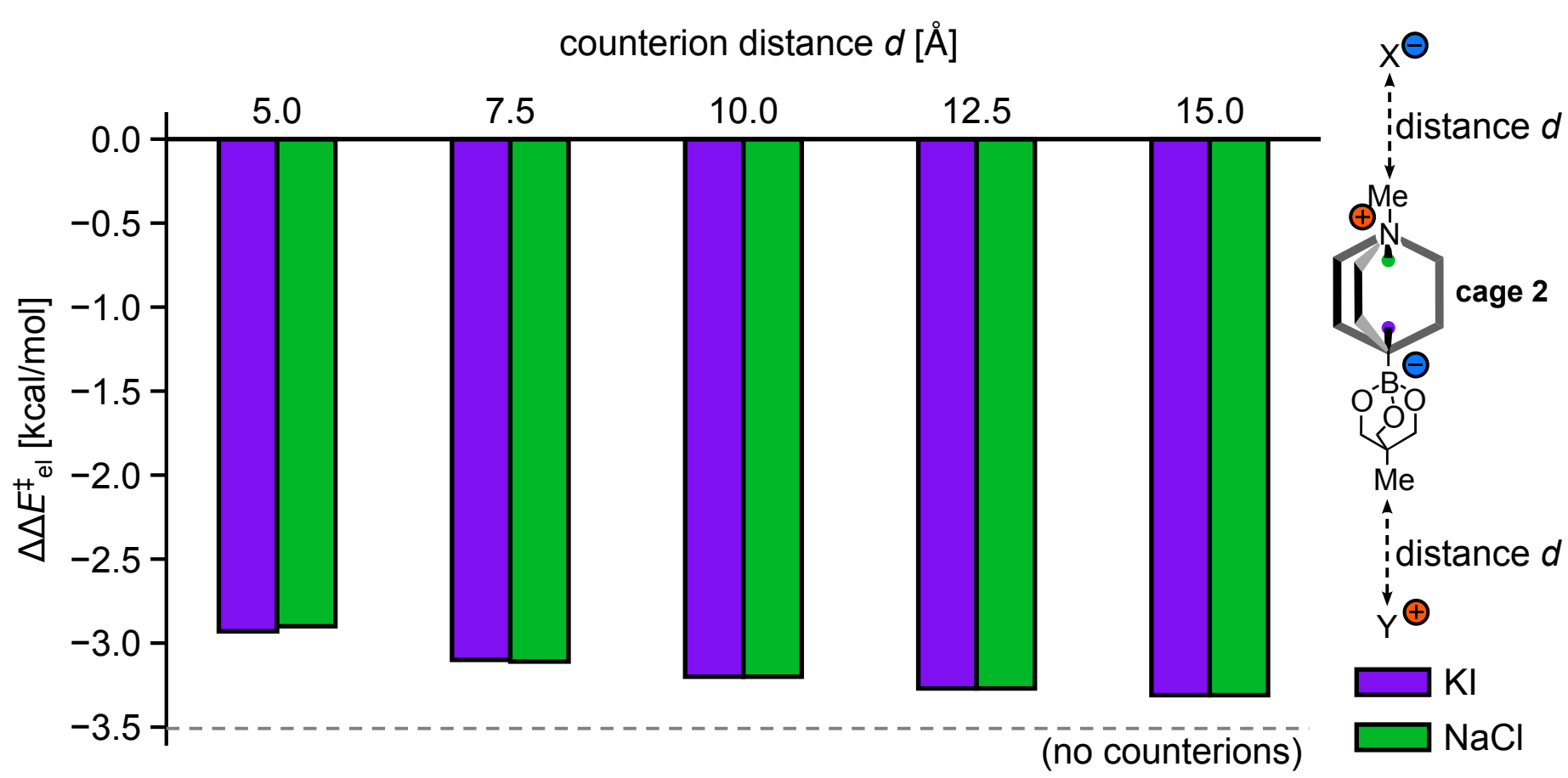

Figure S11: Electronic activation-energy differences, $\Delta\Delta E^{\ddagger}_{el}$, for cage **2** relative to cage **1**, calculated at the PBE0-D3BJ/SMD($CHCl_3$) level without zero-point energy (ZPE) corrections. The systems contain either K^+ below the cage and I^- above it, or Na^+ below the cage and Cl^- above it. Each ion was positioned at a distance d of 5.0–15.0 Å from the corresponding terminal methyl group, as illustrated in the inset. The grey dashed line indicates the value obtained without counterions, $\Delta\Delta E^{\ddagger}_{el} = -3.51$ kcal/mol.

S2.8 External Field Effects

The activation enthalpy differences in cage **1** relative to cage **1** in a zero field were calculated as follows:

$$\Delta\Delta H^{\ddagger} = \Delta H^{\ddagger}_{\text{field}} - \Delta H^{\ddagger}_{\text{zero-field}} \tag{S2}$$

where each activation enthalpy was computed from single-point energy differences on geometries optimised at the respective field strength:

$$\Delta H^{\ddagger} = H^{\ddagger}_{\text{TS}} - H^{\ddagger}_{\text{R}} \tag{S3}$$

All structures were optimised in the gas phase, and solvent effects were included via single-point calculations using SMD($CHCl_3$)-PBE0-D3BJ/def2-SVP.

The correlation between the applied positive z-oriented external field and the resulting activation enthalpy changes $\Delta\Delta H^{\ddagger}$, including zero-point energy (ZPE) corrections is shown in Figure S12. The activation barrier decreases linearly with increasing field strength, with an excellent correlation coefficient ($R^2 = 0.9985$) and a slope of -21.92 (kcal/mol)/(V/Å).

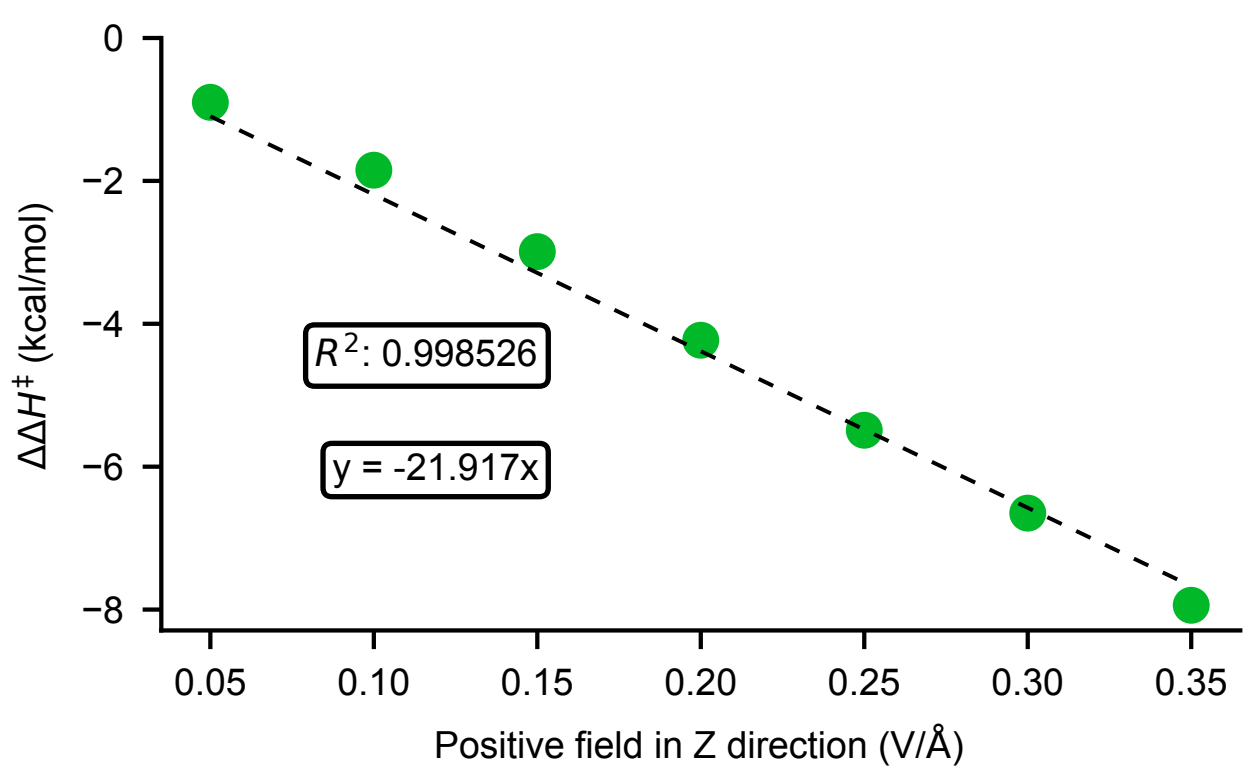

Figure S12: Activation enthalpy difference $\Delta\Delta H^{\ddagger}$ (including ZPE corrections) as a function of external electric field strength applied in the positive z-direction for cage **1**, SMD($CHCl_3$)-PBE0-D3BJ/def2-SVP.

To complement the positive field analysis presented in the main text (Figure 4, positive potential at top, negative potential at bottom), we evaluated the effect of externally applied electric fields in the *opposite* (negative z) direction—that is, with a more negative potential positioned at the top of the cage, closer to the anhydride group (negative field). The transition state remains directional with respect to the cage framework, and the same z-axis convention was used to ensure consistent comparison.

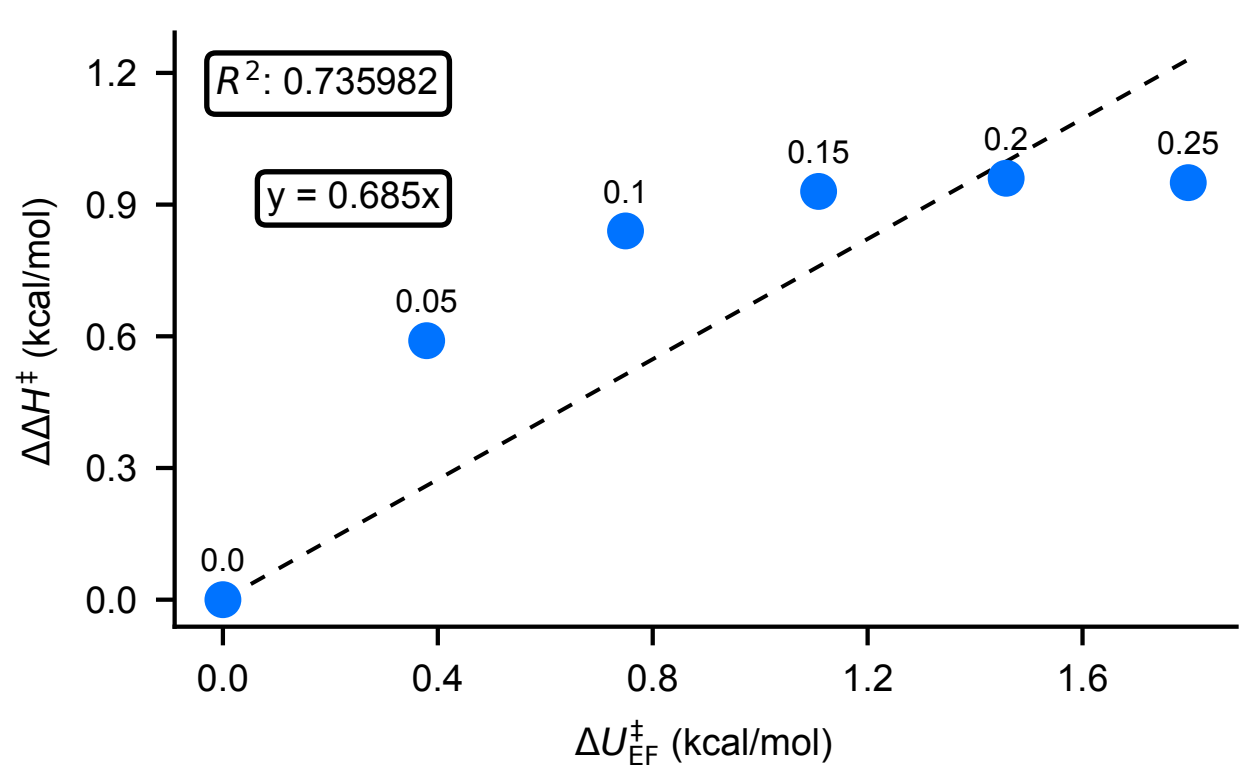

Figure S13: Effect of an external electric field applied in the negative z-direction on the activation enthalpy difference $\Delta\Delta H^{\ddagger}$ in unsubstituted cage **1**, plotted against the corresponding interaction energy $\Delta U^{\ddagger}_{\text{EF}}$ (uncorrected, electronic energies only), SMD($CHCl_3$)-PBE0-D3BJ/def2-SVP. Points are labelled with their EF strength (in V/Å).

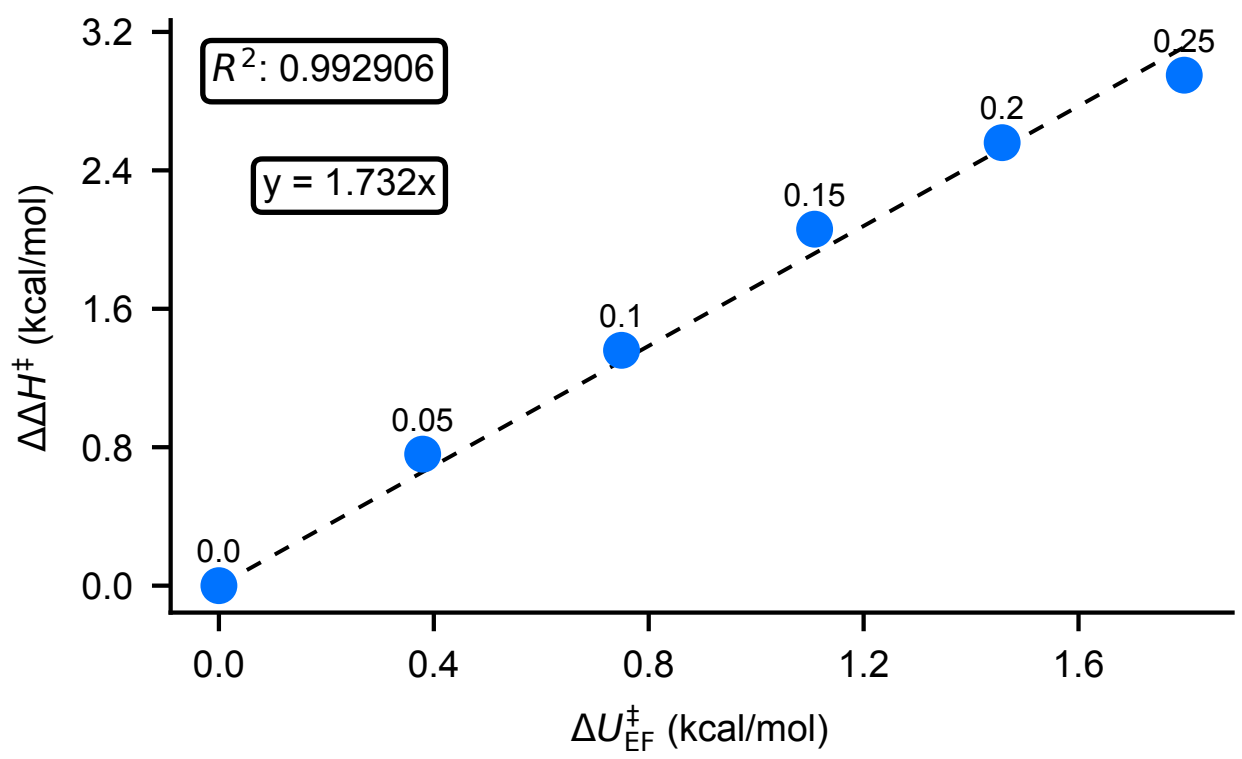

Figure S14: Effect of an external electric field applied in the negative z-direction on the activation enthalpy difference $\Delta\Delta H^{\ddagger}$ in unsubstituted cage **1**, plotted against the corresponding interaction energy $\Delta U^{\ddagger}_{\mathrm{EF}}$ (ZPE corrected electronic energies), SMD($CHCl_3$)-PBE0-D3BJ/def2-SVP. Points are labelled with their EF strength (in V/Å).

We computed the change in activation enthalpy $\Delta\Delta H^{\ddagger}$, relative to the zero-field case, for a series of applied field strengths and plotted it against the corresponding electrostatic interaction energy $\Delta U^{\ddagger}_{\mathrm{EF}}$. Without ZPE corrections (Figure S13), we find a positive correlation ($R^2 = 0.74$, slope = 0.69), while inclusion of ZPE (Figure S14) improves the linearity dramatically ($R^2 = 0.99$), increasing the slope to 1.73.

The high value obtained with ZPE corrections exceeds the theoretical limit of unity expected from a pure dipole–field interaction model, indicating that second-order field-induced effects—such as inductive polarisation or electron density redistribution within the cage—contribute to the stabilisation of the transition structure. These results reinforce the importance of electric field directionality in tuning reactivity and highlight the need to account for vibrational contributions when evaluating field effects computationally.

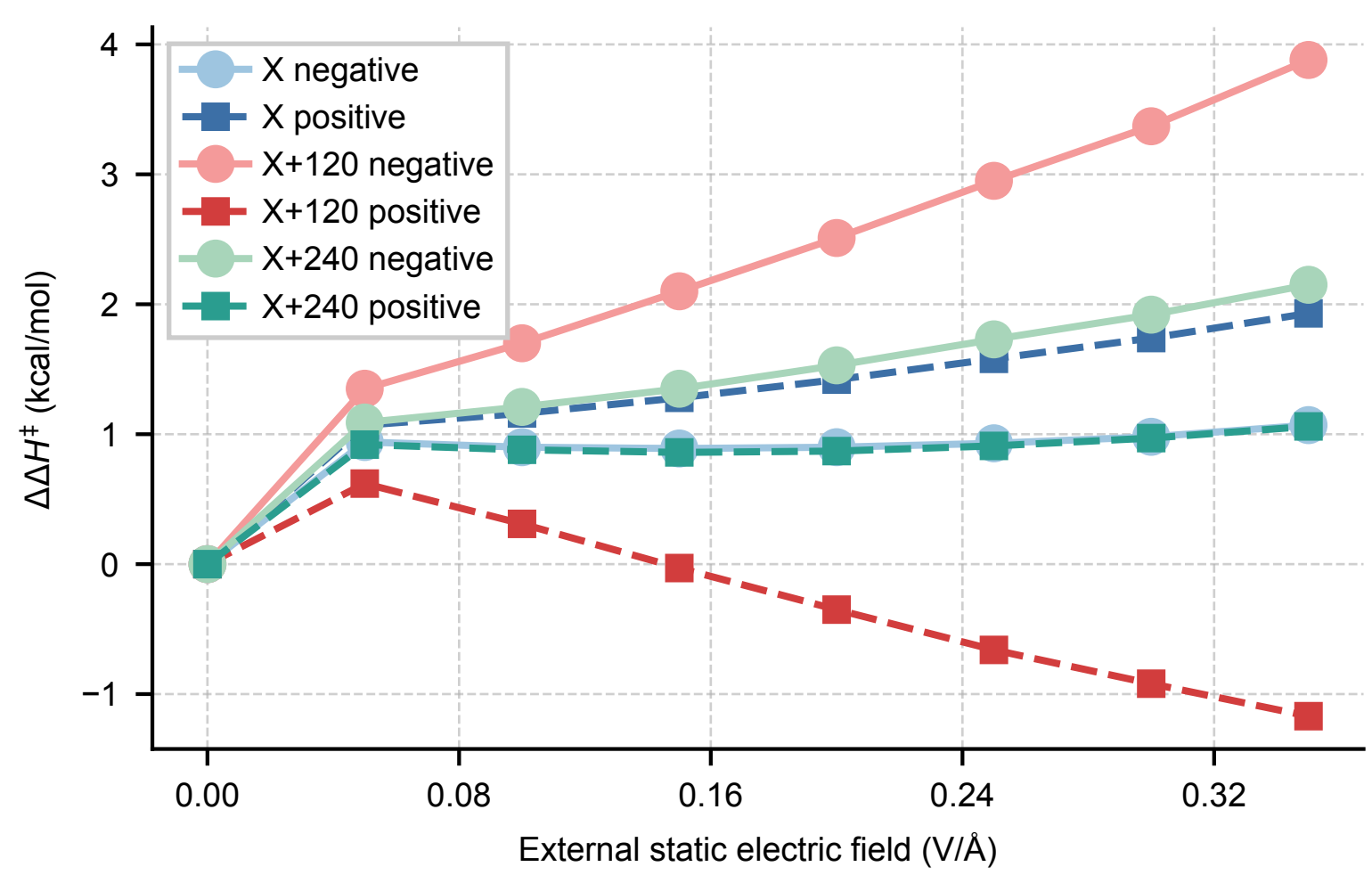

Figure S15: Activation enthalpy changes $\Delta\Delta H^{\ddagger}$ in unsubstituted cage **1** under static external electric fields applied in three in-plane directions, evaluated without structural relaxation at CPCM($CHCl_3$)-PBE0-D3BJ/def2-SVP level of theory.

To examine the spatial sensitivity of cage **1** to external electric fields, we applied static fields in three directions within the molecular plane: along the x-axis, and at $\pm 120^\circ$ and $\pm 240^\circ$ relative to it. Unlike the main analysis along the z-axis, these calculations were performed as single-points on the field-free optimised structures, without reoptimisation under the applied field.

The results, shown in Figure S15, indicate that while catalytic effects remain modest compared to the z-oriented field, directional trends are still present. The positive and negative field orientations (solid and dashed lines, respectively) lead to linear changes in activation enthalpy $\Delta\Delta H^{\ddagger}$, suggesting that in-plane fields also perturb the electronic structure

of the cage and $TS_1^{\ddagger}$, but to a lesser extent than the z-axis. These effects are likely due to anisotropic polarisation and small projections of the activation dipole onto the field vector.

S2.9 Electrostatic Potential Maps

To examine how substituent charges influence the electrostatic environment of the cage interior, we computed and visualised ESP distributions across relevant planes. These maps form the basis of the finite capacitor model (FCM) introduced in the main text, which represents each substituent charge as a localised source generating an internal electric field.

To justify the assumptions of the finite capacitor model (FCM), we visualised the electrostatic potential (ESP) of cage **5** across three planes relevant to substituent-induced field generation:

(a) 1 Bohr above the formally positively charged substituent (values closer the the atoms began probing the nuclear charge)

(b) 1 Bohr below the formally negatively charged substituent

(c) through the geometric center of the cage and reaction site.

The ESP was computed using the `orca_vpot` utility in ORCA with the PBE0 functional and visualised as both 2D maps and 1D profiles along the central horizontal axis (Figures S16–S18).

In all cases, the ESP is strongly localised near the formally charged atoms and their immediate bonding environment. The potential decays rapidly within 2–3 Å from the charge center, indicating that the spatial distribution of field-generating charge is relatively compact. Based on these profiles, we chose a disk radius of $r = 2.18$ Å to represent the effective area over which the substituent charge is distributed in the FCM. This corresponds approximately to 1.5 times the average bond length around the charged atoms, capturing both the point charge and its immediate electrostatic influence.

In contrast, the ESP in the mid-plane of the cage (Figure S18) shows limited structure, as the field at this point results from multiple overlapping atomic contributions within the dense cage interior. The lack of a clear peak or dip in the ESP at the reaction site highlights the difficulty of decomposing the substituent-induced field in this region directly. Nevertheless, the ESP distributions near the substituents justify the use of a localised charge model in the FCM. This approach allows the model to quantitatively reproduce the electrostatic field felt at the transition state in all charged cages and supports its application for rapid field estimation based on empty cage geometries.

Importantly, we observe similar ESP localisation behavior across all substituted cages, suggesting that the use of a single effective disk radius is both reasonable and broadly applicable. The value of $r = 2.18$ Å therefore provides a consistent and physically motivated choice for modeling field-generating substituents in the FCM framework.

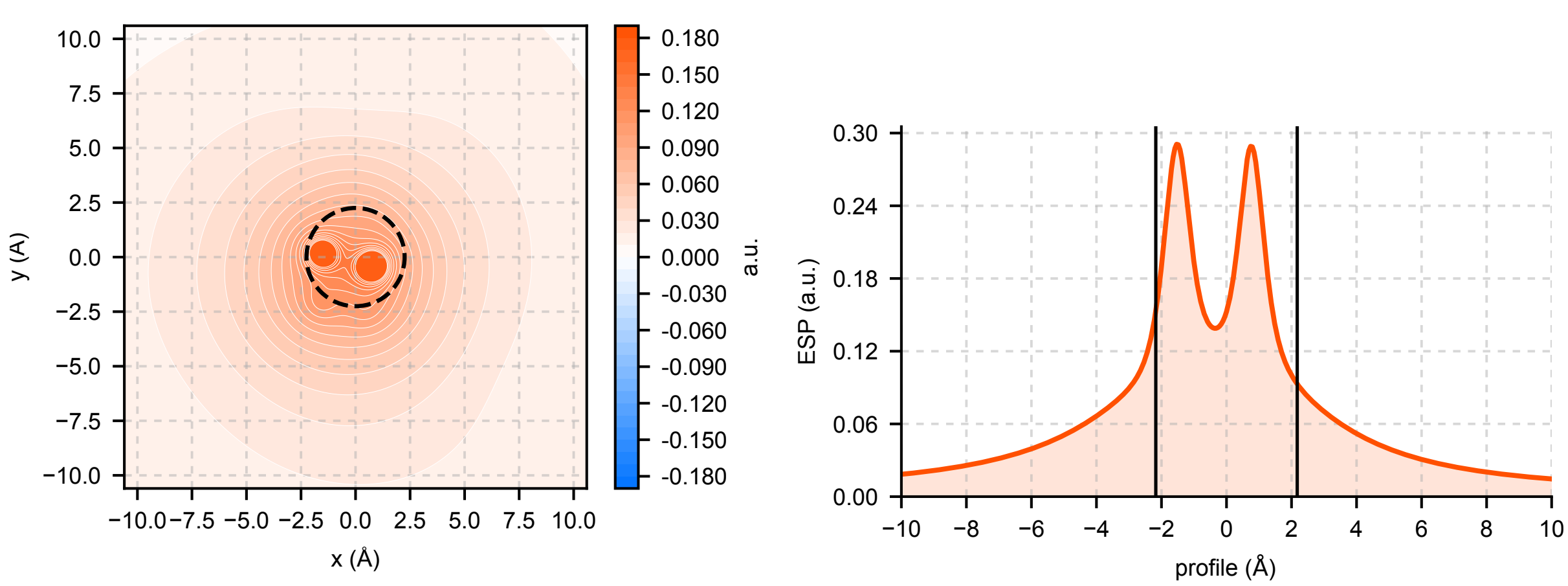

Figure S16: Electrostatic potential (ESP) above the positively charged substituent in cage **5**, computed on a plane 1 Bohr above the formal charge centre. Left: 2D ESP map; right: 1D ESP profile along the central axis of the map. The black dashed circle (left) and black vertical lines (right) denote the chosen disk radius of $r = 2.18$ Å, corresponding to the effective spatial extent of the substituent charge used in the FCM.

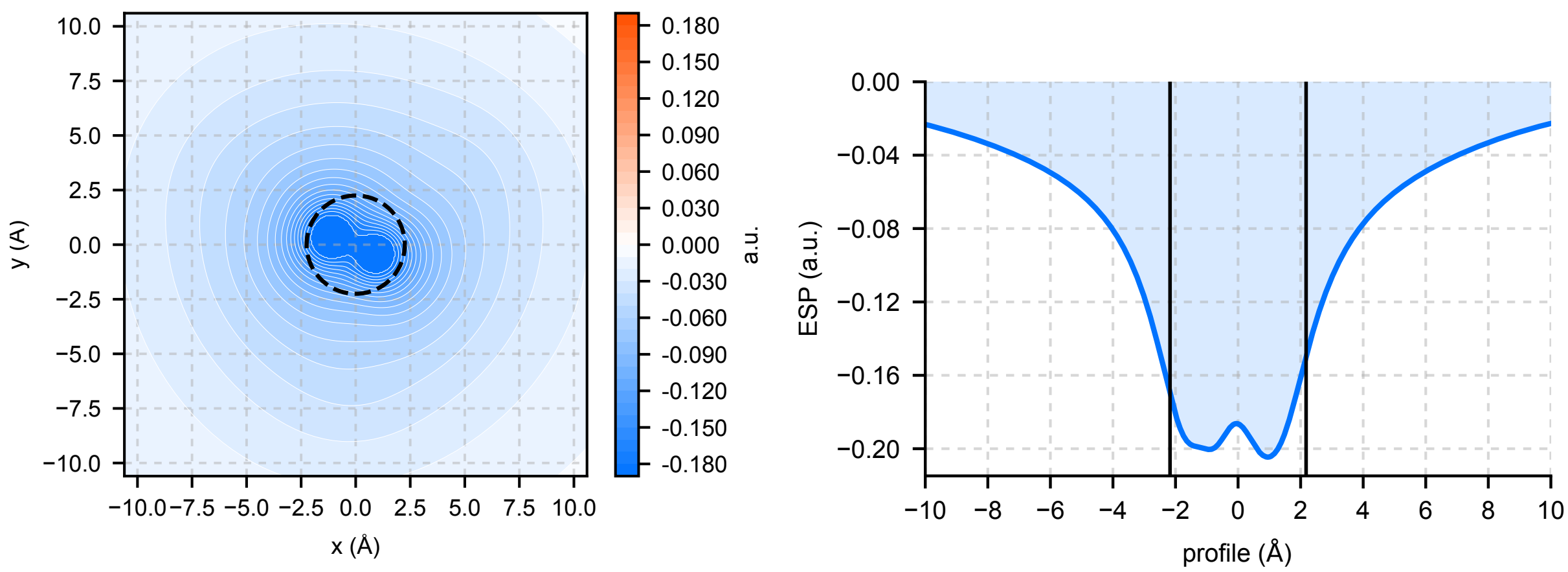

Figure S17: Electrostatic potential (ESP) below the negatively charged substituent in cage **5**, computed on a plane 1 Bohr below the formal charge center. Left: 2D ESP map; right: 1D ESP profile along the central axis of the map. The black dashed circle (left) and black vertical lines (right) denote the chosen disk radius of $r = 2.18$ Å, corresponding to the effective spatial extent of the substituent charge used in the FCM.

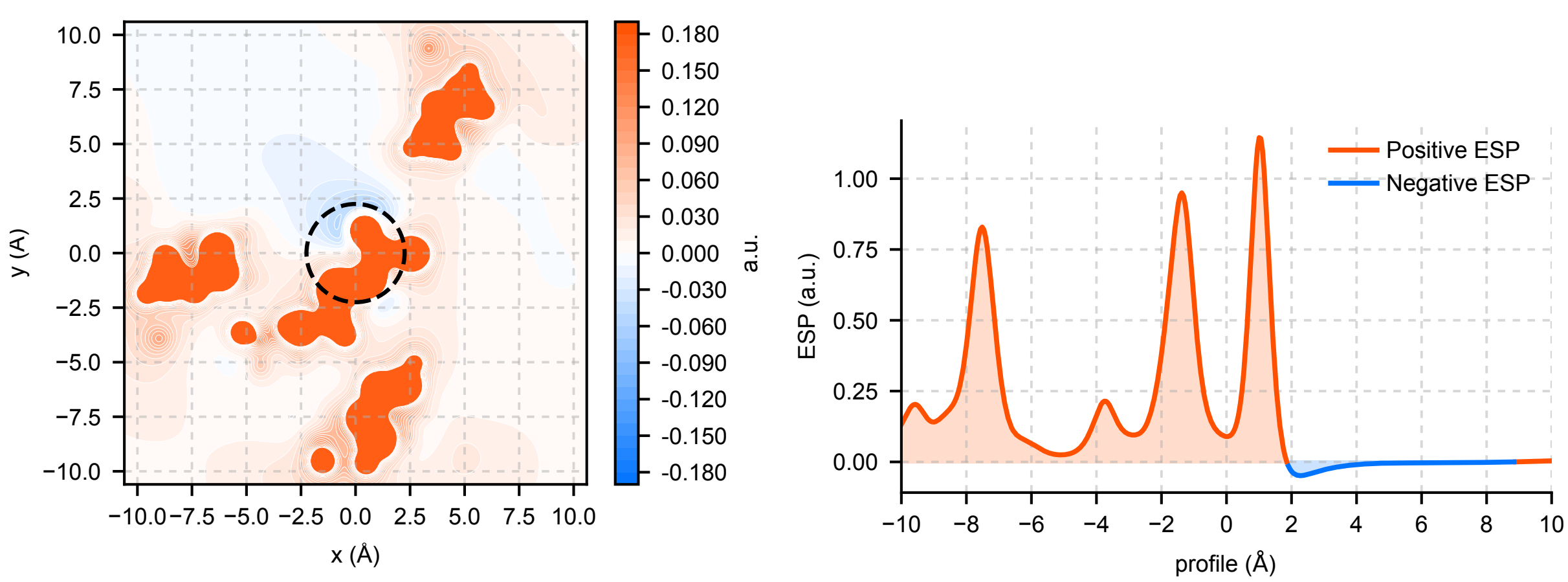

Figure S18: Electrostatic potential (ESP) in the mid-plane of cage **5** passing through the reaction site, computed at the center between A and B substituents. Left: 2D ESP map; right: 1D ESP profile across the horizontal axis. The black dashed circle (left) denote the chosen disk radius of $r = 2.18$ Å, corresponding to the effective spatial extent of the substituent charge used in the FCM.

S2.10 Finite Capacitor Model Validation

We evaluated the robustness of the FCM used to estimate local electric field effects in substituted cages. This includes testing its sensitivity to the choice of disk radius and comparing predictions using dipole moments from either transition state or empty cage geometries.

To evaluate the sensitivity of the finite capacitor model (FCM) to the chosen disk radius r, we computed $\Delta U^{\ddagger}_{\mathrm{EF}}$ values using two alternative radii: 1.5 Å and 3.0 Å. These values bracket our chosen standard of $r = 2.18$ Å, corresponding to approximately 1.5 times the average bond length around the formally charged substituent atoms.

Figures S19 and S20 show that the correlation between $\Delta U^{\ddagger}_{\mathrm{EF}}$ and $\Delta\Delta H^{\ddagger}$ is largely unaffected by the choice of r. In both cases, the coefficient of determination remains nearly constant ($R^2 \approx 0.91$). This indicates that although the slope of the correlation varies with r, the ranking of cage performance—and thus the identity of the best-performing substituents—remains stable. As such, the FCM retains its predictive power across a broad range of reasonable disk radii, supporting our use of a single representative value $r = 2.18$ Å throughout.

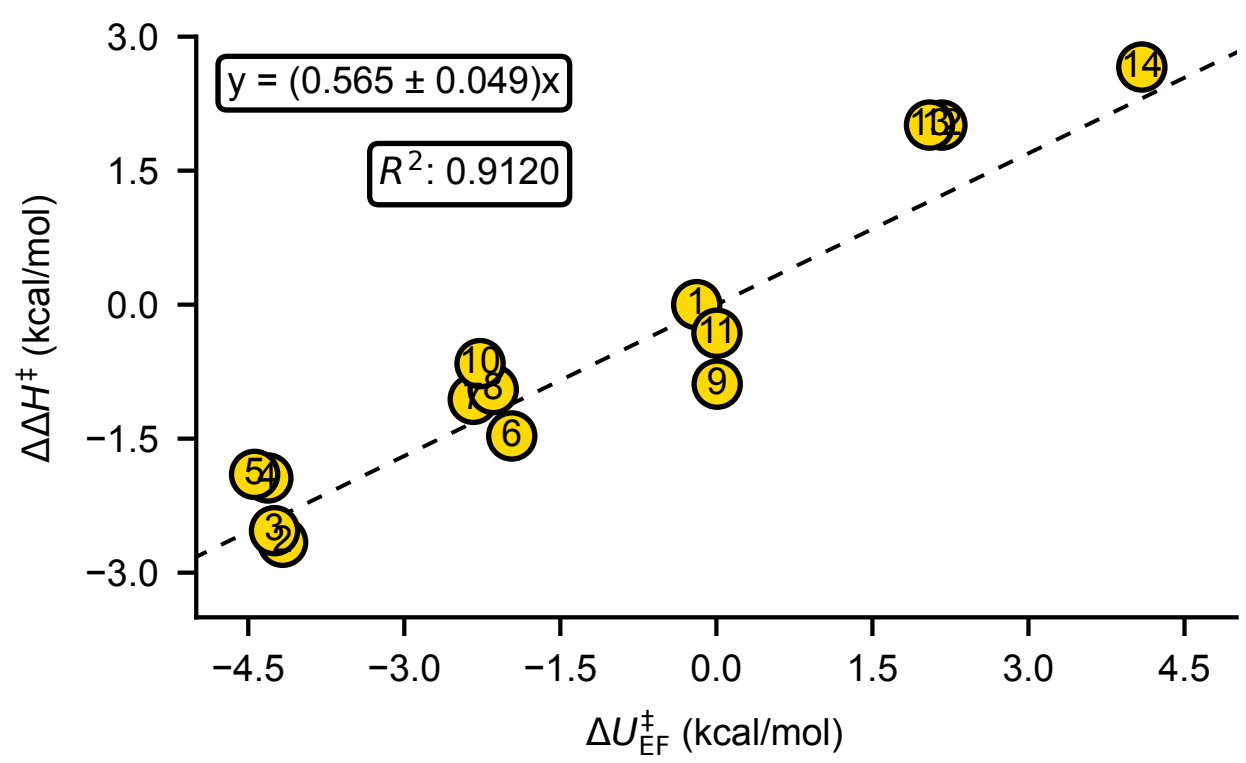

Figure S19: Correlation between computed activation enthalpy changes $\Delta\Delta H^{\ddagger}$ and electrostatic interaction energies $\Delta U^{\ddagger}_{\mathrm{EF}}$ for cages **1**–**14** calculated using the finite capacitor model with a disk radius of 1.5 Å.

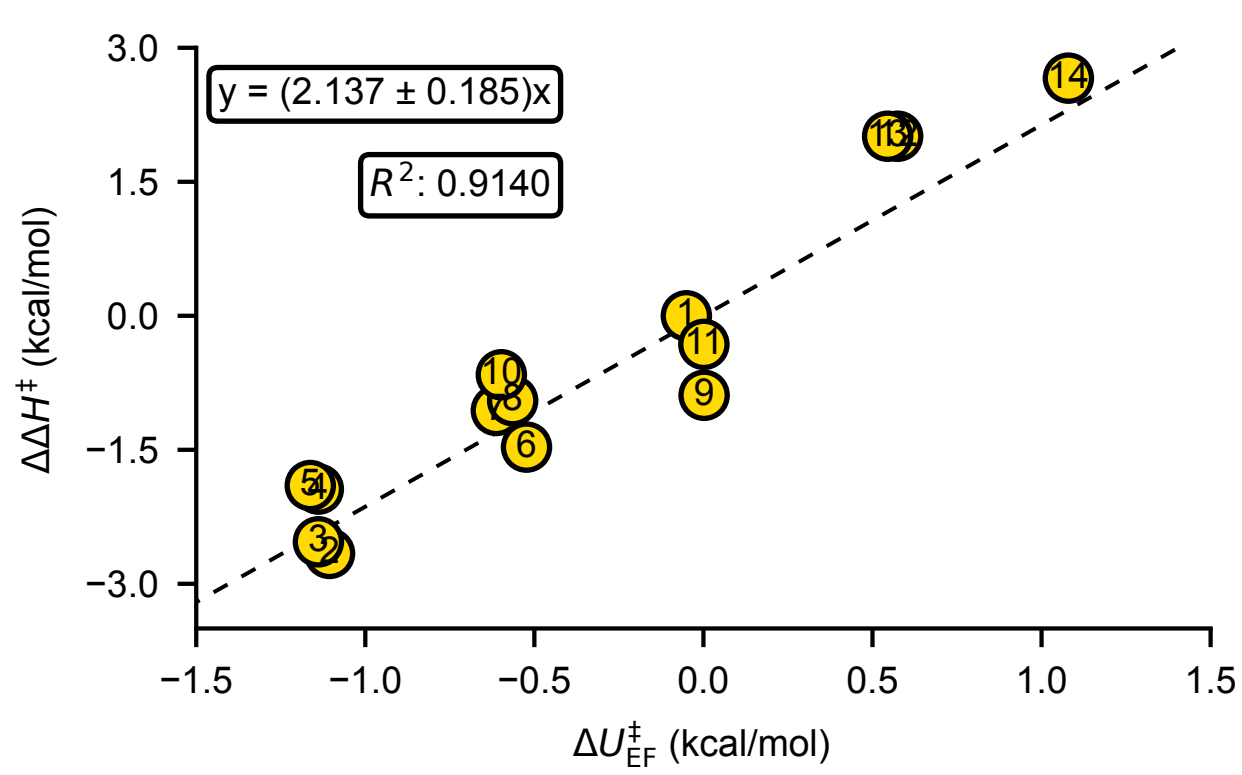

Figure S20: Correlation between computed activation enthalpy changes $\Delta\Delta H^{\ddagger}$ and electrostatic interaction energies $\Delta U^{\ddagger}_{\mathrm{EF}}$ for cages **1**–**14** calculated using the finite capacitor model with a disk radius of 3.0 Å.

To evaluate the reliability of the finite capacitor model (FCM) in predicting the catalytic response to local electric fields, we compared its field estimates against explicitly applied field calculations in GAUSSIAN. In Figure S22, the grey points represent reference activation enthalpy differences $\Delta\Delta H^{\ddagger}$ computed for cage **1** under a series of externally applied fields along the reaction axis. These values serve as a benchmark for electrostatic barrier modulation.

The yellow datasets in each plot correspond to substituted cages **1**–**14**, where the electric field at the reaction site was estimated using the FCM. In Figure S21, FCM fields were calculated using the activation dipole moment difference $\Delta\mu^{\ddagger}$ of each individual transition state. In Figure S22, the same analysis was performed using only dipole moments from the empty cages, thereby avoiding transition state optimisations.

Both approaches show strong inverse correlations between the estimated field strength and the reaction barrier ($R^2 = 0.919$ and $R^2 = 0.910$, respectively), with similar slopes (approximately -13.0 to -12.5 (kcal/mol)/(V/Å)). These results demonstrate that the FCM captures the key physics of field-induced catalysis and that using ground-state dipoles alone offers a computationally efficient screening strategy. The explicit field results further support the linear field dependence and validate the electrostatic model underlying this analysis.

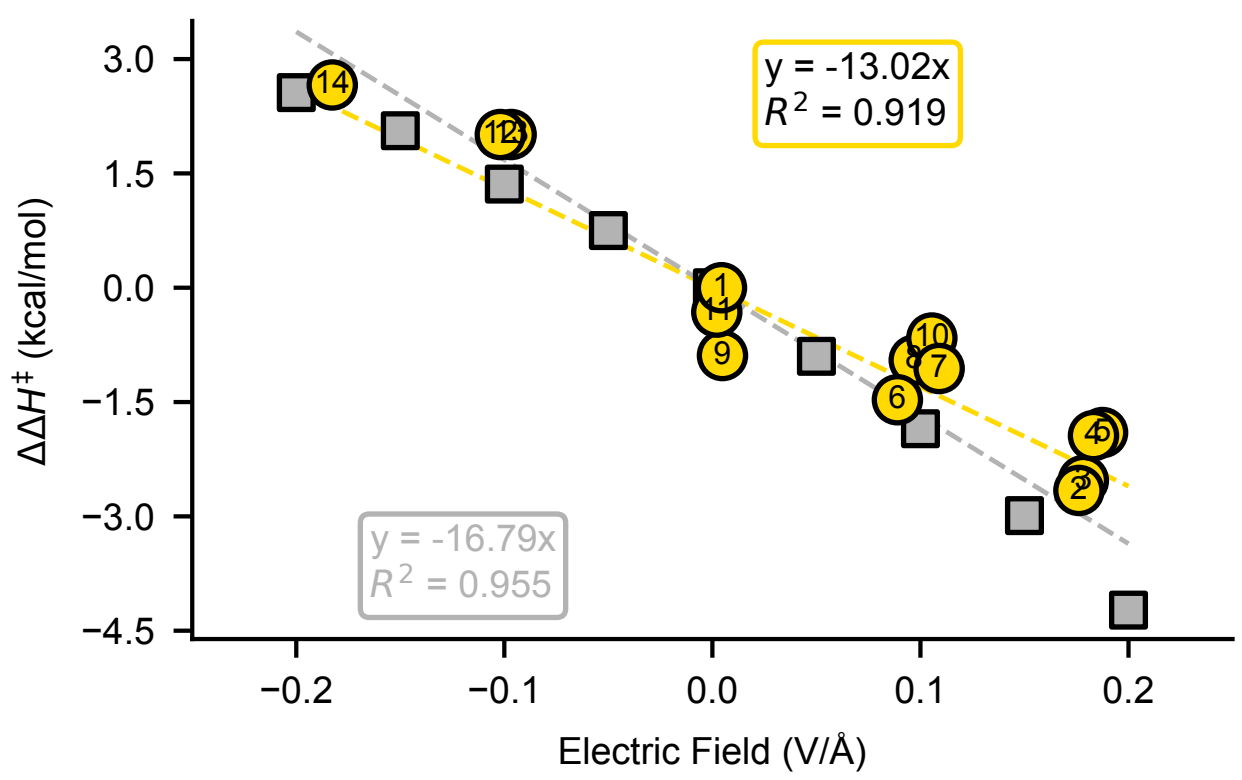

Figure S21: Correlation between activation enthalpy differences $\Delta\Delta H^{\ddagger}$ and electric field strengths at the reaction site. Grey points: reference values for cage **1** under externally applied fields (computed using Gaussian). Yellow points: substituted cages **1–14** with fields estimated using the FCM.

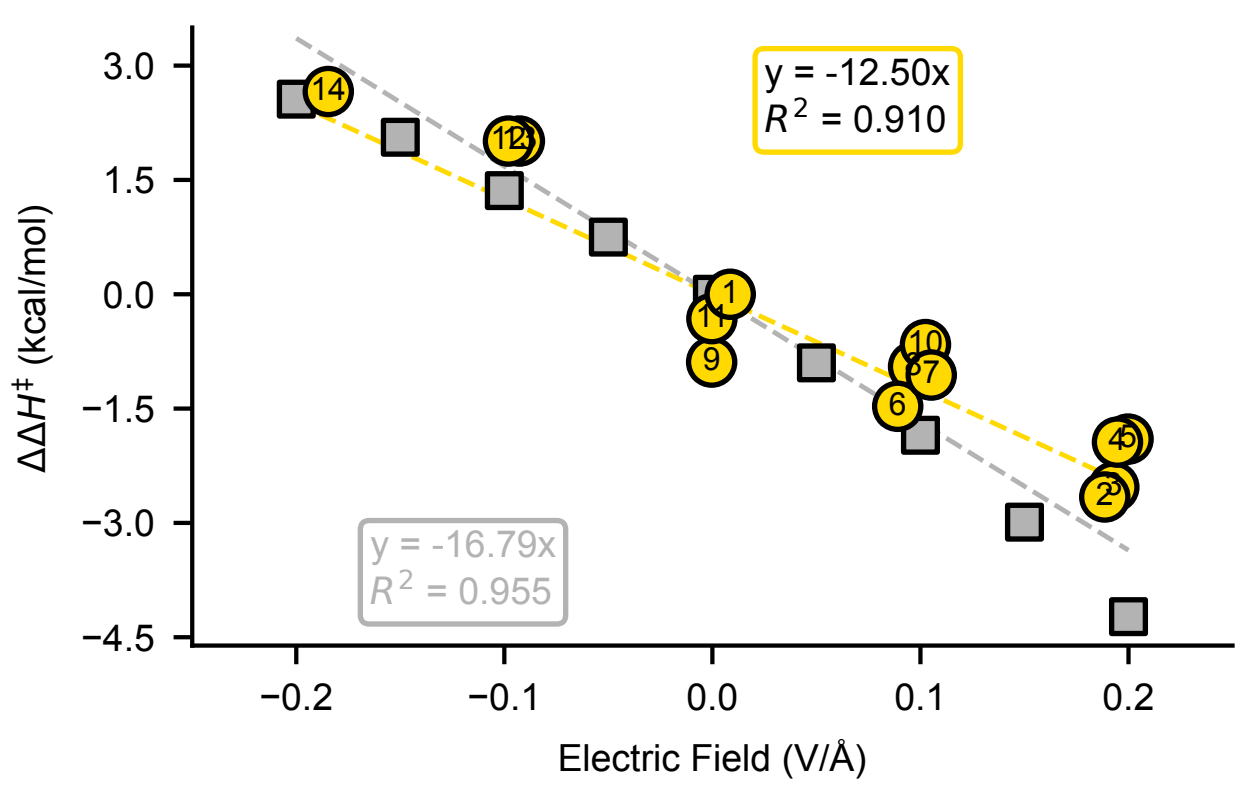

Figure S22: Same analysis as Figure S21, but using dipole moments from the empty cage geometries for field estimation via the FCM. The grey reference dataset is identical in both panels.

S2.11 Numerical data

Table S2: Computed activation enthalpies $\Delta\Delta H^{\ddagger}_{\mathrm{PBE0}}$, electrostatic interaction energies $\Delta U^{\ddagger}_{\mathrm{EF}}$, and activation dipole moments $\Delta\mu^{\ddagger}$ for the unsubstituted cage **1** under increasing external electric fields. All $\Delta\Delta H^{\ddagger}$ values include zero-point energy (ZPE) corrections. Activation enthalpies, interaction energies and dipole moments were obtained from single-point calculations at the SMD($CHCl_3$)-PBE0-D3BJ/def2-SVP level of theory.

Explicit electric field (V/Å)	$\Delta\Delta H^{\ddagger}_{\mathrm{PBE0}}$ (kcal/mol)	$\Delta U^{\ddagger}_{\mathrm{EF}}$ (kcal/mol)	$\Delta\mu^{\ddagger}$ (Debye)
-0.25	2.95	1.80	1.25
-0.20	2.56	1.46	1.26
-0.15	2.06	1.11	1.28
-0.10	1.36	0.75	1.29
-0.05	0.76	0.38	1.32
0.00	0.00	0.00	2.24
0.05	-0.90	-0.85	4.10
0.10	-1.85	-2.08	6.84
0.15	-2.99	-3.73	9.88
0.20	-4.23	-5.64	12.86
0.25	-5.49	-7.71	15.76
0.30	-6.65	-9.71	18.32
0.35	-7.94	-11.73	21.15

Table S3: Computed electrostatic interaction energies $\Delta U^{\ddagger}_{\mathrm{EF}}$ and induced electric field strengths for the corresponding empty cages. All values were obtained from single-point calculations at the SMD($CHCl_3$)-PBE0-D3BJ/def2-SVP level of theory.

Cage	$\Delta U^{\ddagger}_{\mathrm{EF}}$ (kcal/mol)	Induced electric field (V/Å)
1	-0.09	0.001
2	-2.03	0.189
3	-2.07	0.193
4	-2.09	0.195
5	-2.15	0.200
6	-0.96	0.089
7	-1.13	0.105
8	-1.04	0.097
9	0.00	0.000
10	-1.10	0.102
11	0.00	0.000
12	1.06	-0.098
13	1.00	-0.093
14	1.98	-0.185

Table S4: Computed activation enthalpy differences $\Delta\Delta H^{\ddagger}$ using PBE0 and CCSD(T) methods, electrostatic interaction energies $\Delta U^{\ddagger}_{EF}$, induced electric field strengths, and activation dipole moments $\Delta\mu^{\ddagger}$(A,B) for the cage transition structures. All $\Delta\Delta H^{\ddagger}$ values include zero-point energy (ZPE) corrections. Interaction energies, induced field strengths, and dipole moments were obtained from single-point calculations at the SMD($CHCl_3$)-PBE0-D3BJ/def2-SVP level of theory.

Cage	$\Delta\Delta H^{\ddagger}_{PBE0}$ (kcal/mol)	$\Delta\Delta H^{\ddagger}_{CCSD(T)}$ (kcal/mol)	$\Delta U^{\ddagger}_{EF}$ (kcal/mol)	Induced electric field (V/Å)	$\Delta\mu^{\ddagger}$(A,B) (Debye)
1	0.00	0.00	-0.10	0.004	2.24
2	-2.66	-2.85	-4.16	0.176	4.56
3	-2.53	-2.79	-4.82	0.178	5.12
4	-1.94	-2.50	-4.81	0.183	5.06
5	-1.90	-2.62	-5.13	0.188	5.31
6	-1.47	-1.36	-2.22	0.090	4.69
7	-1.06	-0.31	-2.64	0.109	4.69
8	-0.95	-0.96	-2.57	0.097	5.01
9	-0.89	0.47	0.00	0.005	2.71
10	-0.66	-0.43	-2.55	0.105	4.69
11	-0.32	-0.27	0.00	0.002	2.80
12	2.01	2.39	0.01	-0.102	0.02
13	2.01	1.20	-0.06	-0.097	-0.13
14	2.66	2.44	-0.69	-0.183	-0.85

S2.12 Geometrical Analysis of the TS Complex

We evaluated the key bond lengths, atom distances, and bond angles in the transition states for the cages **1–14** to ascertain whether any correlation could be found between these parameters and the negative (favorable) $\Delta\Delta H^{\ddagger}$ found in cages with a positive field (positive A, negative B).

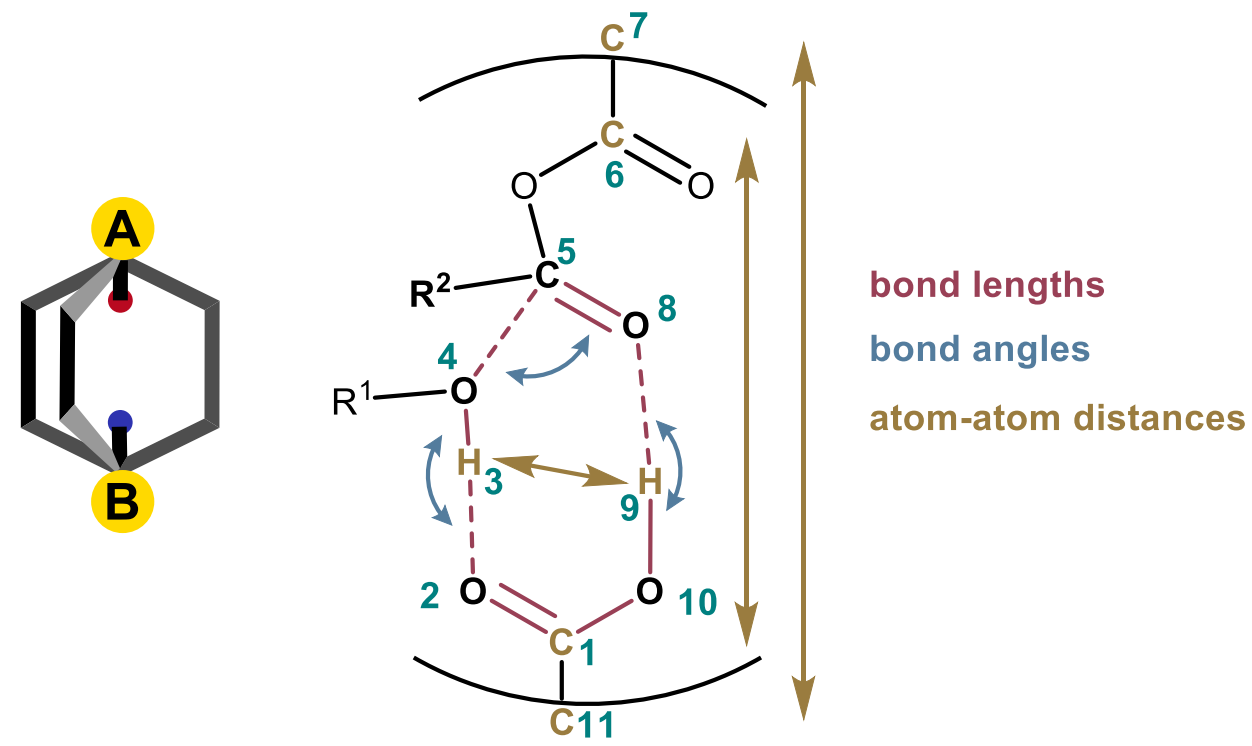

Figure S23: Numbering scheme for atoms in the transition state in cages **1–14**, where **A** is at the top, **B** at the bottom, and a positive field indicates a more negative **B**.

Cage Height: The cage heights are almost invariant across structures **1–14** ($\bar{x}_{C1-C6}$ = 5.72 Å, σ_{C1-C6} = 0.04 Å), demonstrating that the **A** and **B** functional groups and associated fields do not strongly perturb the distance between the two acid groups that promote catalysis in the transition state (Figure S24).

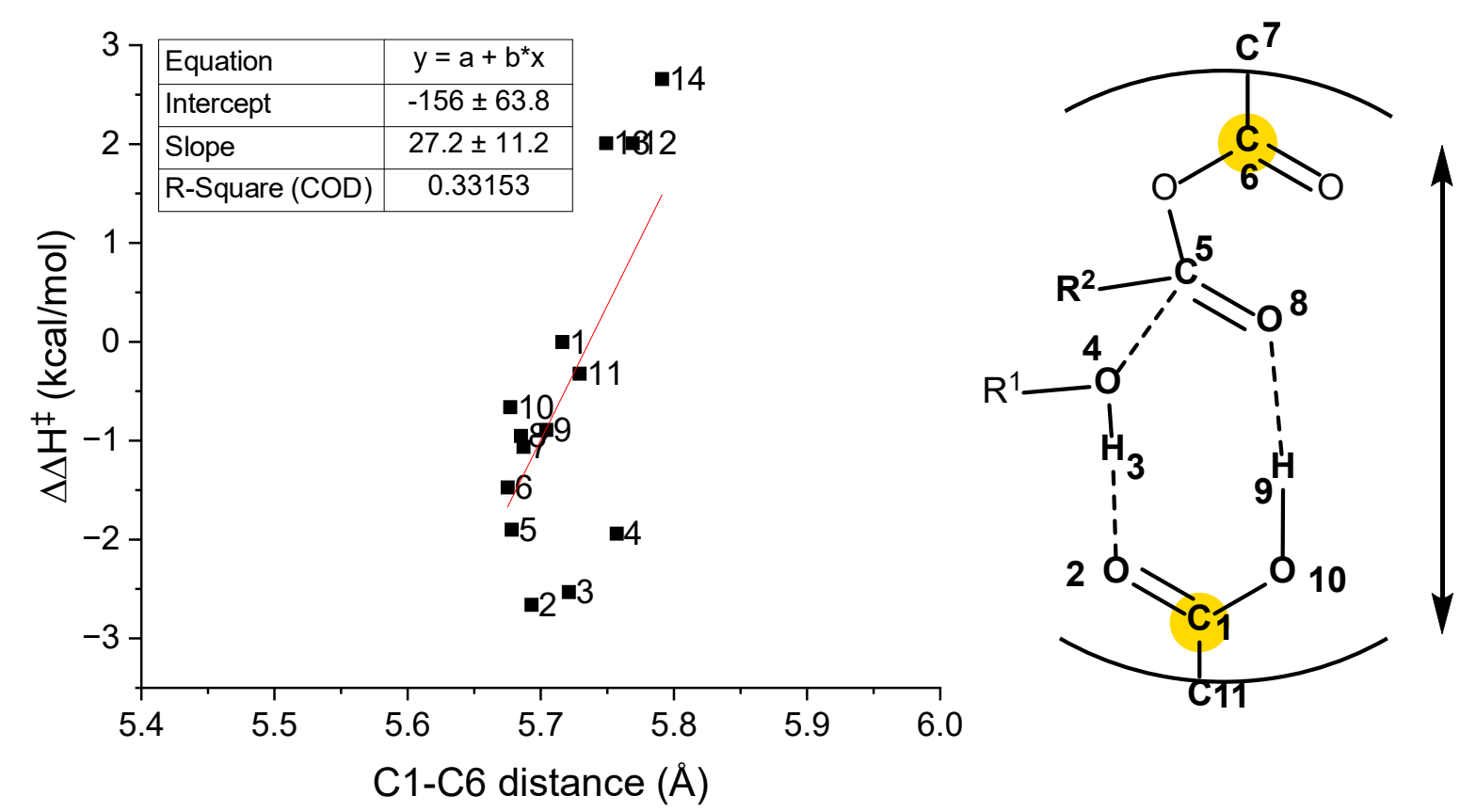

Figure S24: Parameters extracted from the transition states found in cages **1–14** using PBE0-D3BJ/def2-SVP and the change in $\Delta\Delta H^{\ddagger}$. Cage cavity height (C1–C6 atom-atom distance) shows 0.1 Åvariation, with no correlation between cavity height and $\Delta\Delta H^{\ddagger}$ for catalytically efficient cages **2–5**.

The Role of H9: The correlation between $\Delta\Delta H^{\ddagger}$and bond lengths involving the acidic proton H9 (bond lengths: O8–H9 and H9–O10) in the transition state, is linear (R_{O8-H9} = 0.9427; R_{H9-O10} = 0.8938), with lesser protonation of the acyl donor correlated with more negative (more favorable) $\Delta\Delta H^{\ddagger}$ (Figure S25). This correlates with the cage field polarity (proton moves towards the most negative end), but does not make intuitive sense for reducing $\Delta\Delta H^{\ddagger}$, since one would expect a shorter R_{O8-H9} value if protic activation of the carbonyl oxygen was energetically important. In other words, a less protonated electrophile is correlated with a reduced barrier, against expectation. The average value of R_{O8-H9} and R_{H9-O10} across the series (shown in blue) gives another clue: it is close to the zero-field value (cage 1, 1.20 Å) for all species. This shows O10 and O8 barely move, and that the proton H9 is positioned by the overall field.

The bond angle for O8–H9–O10 is closer to 180° for cages with a more stabilised (negative) $\Delta\Delta H^{\ddagger}$, in line with expectation, since H-bonds closer to 180° are stronger (Figure S26).

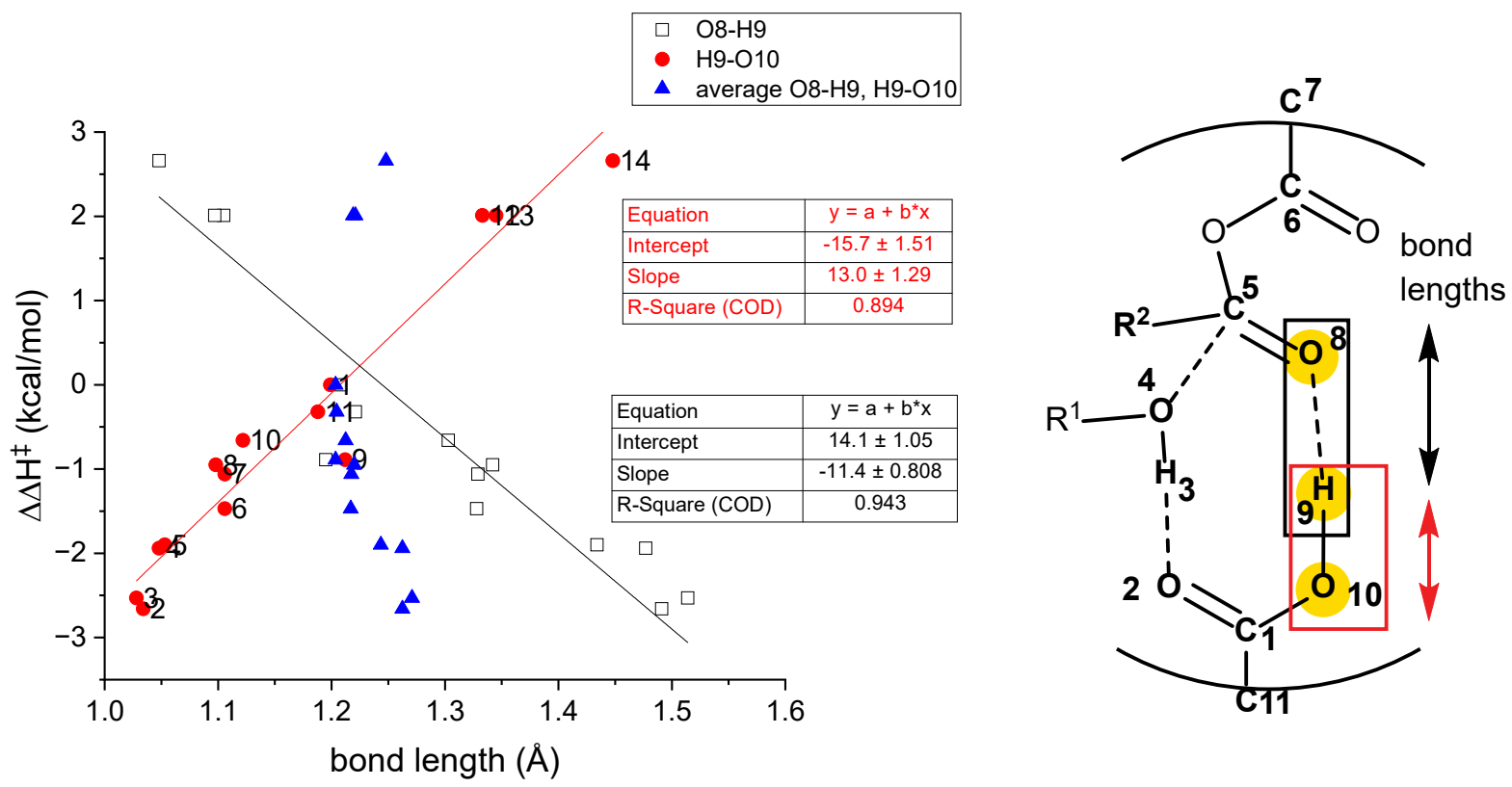

Figure S25: Parameters extracted from the transition states found in cages **1–14** using PBE0-D3BJ/def2-SVP and the change in $\Delta\Delta H^{\ddagger}$. Atom-atom distances for O8–H9 (black) and H9–O10 (red) and the average of the two values (blue) for each of cage **1–14** are shown. There is an inverse correlation between $\Delta\Delta H^{\ddagger}$ and R_{O8-H9} for all cages (R_{O8-H9} increases as $\Delta\Delta H^{\ddagger}$ decreases), indicating the field effect does not work due to electrophilic activation of O8.

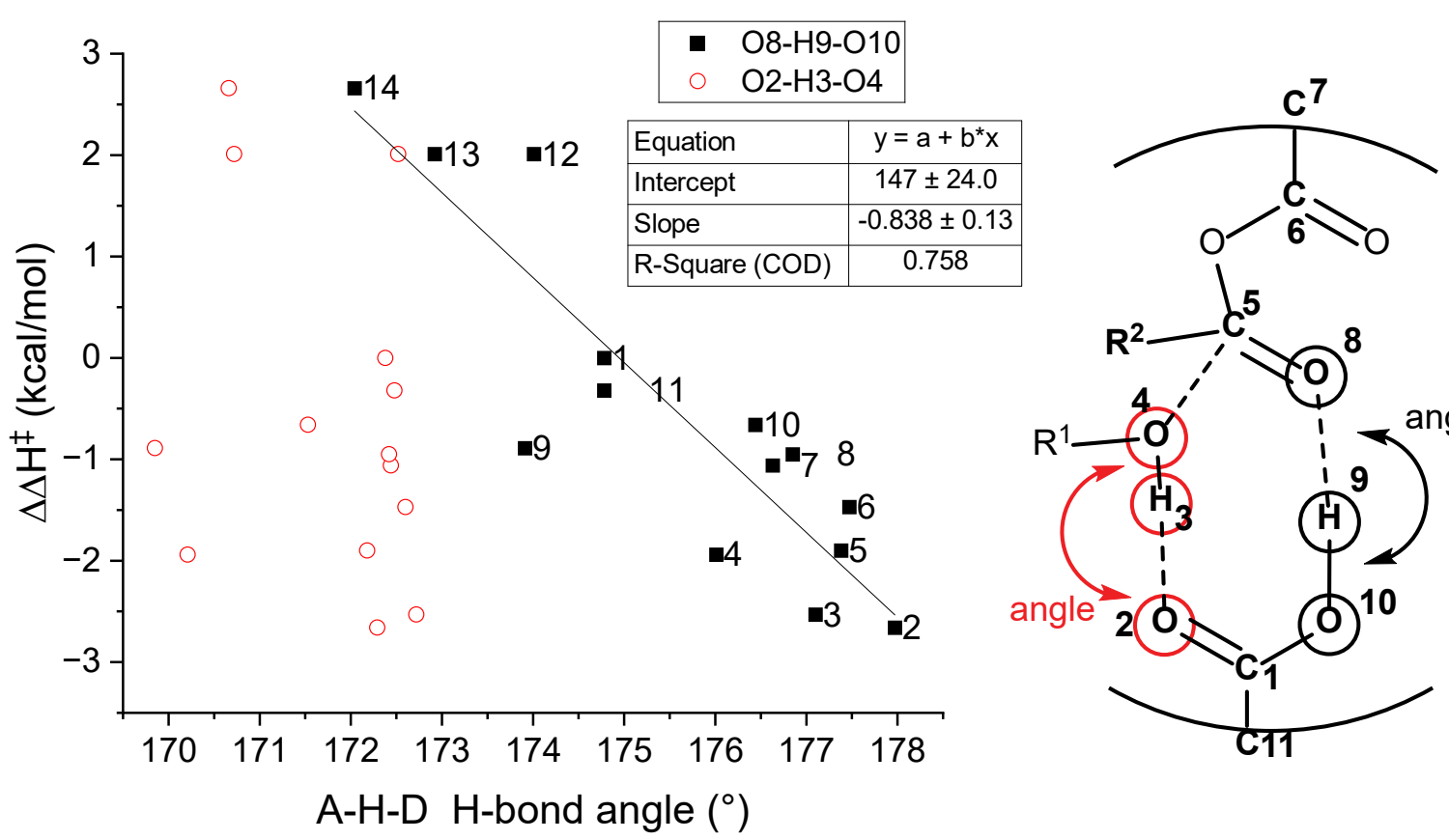

Figure S26: Parameters extracted from the transition states found in cages **1–14** using PBE0-D3BJ/def2-SVP and the change in $\Delta\Delta H^{\ddagger}$. Acceptor-H-Donor bond angles for O8,H9,O10 and O2,H3,O4. There is no correlation between $\Delta\Delta H^{\ddagger}$ and the O2,O3,O4 bond angle, and a moderate correlation between $\Delta\Delta H^{\ddagger}$ and the O2,O3,O4 bond angle, with stronger (more linear) bonds equating to a more stabilised TS (e.g. cages **2–5**), though the correlation if just considering cages **2–5** is not convincing.

The Role of H3: The angle of the H-bond O2–H3–O4 is $\approx 172^{\circ}$ with a standard deviation of about 1°, indicating no real change across different cage TS structures. (Figure S26). The alcohol RO–H bond length, H3–O4, increases with decreasing $\Delta\Delta H^{\ddagger}$ until $\Delta\Delta H^{\ddagger} < -1$ kcal/mol, after which it remains constant at 1.14 Å. The acceptor bond length, O2–H3, shortens from 1.49 Å to 1.26 Å with decreasing $\Delta\Delta H^{\ddagger}$ until $\Delta\Delta H^{\ddagger} < -1$ kcal/mol, after which it also remains constant. In other words, the average bond lengths of H2–O3 and H3–O4 become invariant once $\Delta\Delta H^{\ddagger} < -1$ kcal/mol suggesting this parameter does not dominate the field-stabilisation of the TS for most proficient cages **2–5**. (Figure S27). This data indicates that the lengthening of the alcohol oxygen–proton bond in the transition state is only a partial contributor to the stabilisation of the transition state in negative (favorable) fields.

Role of the New Carbon-Oxygen Bond: The most important bond parameter appears to be the new bond forming between O4 and C5. The favorable field in cage **2** permits a O4–C5 incipient bond length of 1.61 Å at the TS, which lengthens to 1.83 Å in the unfavorable field of cage **14**, with a linear correlation found across the cages **1–14** (R_{O4-H5} = 0.7603). Note that the breaking acyl C5–O8 double bond is *also* shorter in negative fields (e.g. cage **2**), despite the closer nucleophile approach (Figure S28). This "later" transition state with respect to C–O bond formation, which greater resembles the tetrahedral intermediate, reflects the apparent increased charge stabilisation of the acyl donor carbonyl at the positive end of the field (towards positive **A**), allowing closer nucleophile approach. There may be a

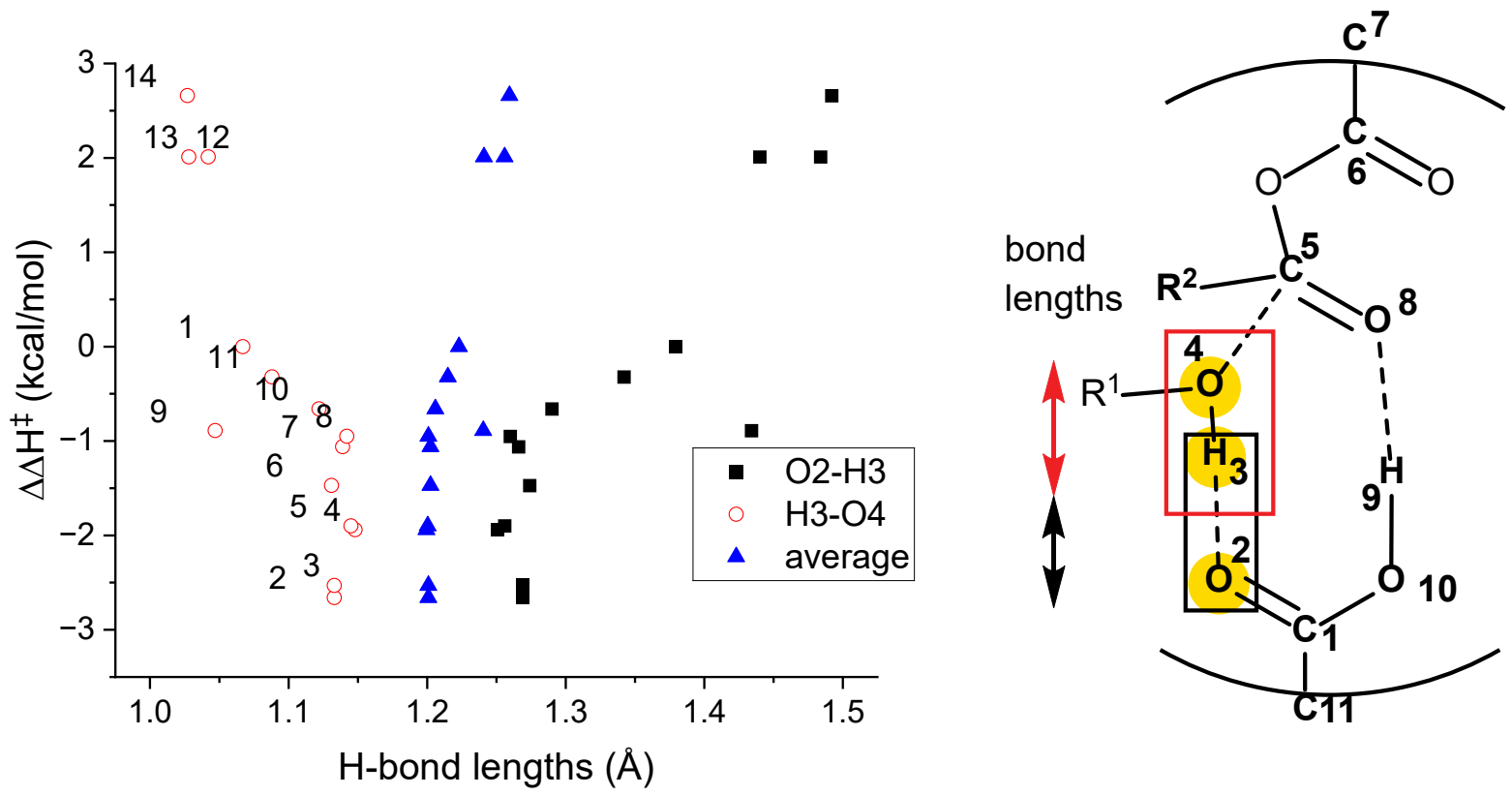

Figure S27: Parameters extracted from the transition states found in cages **1–14** using PBE0-D3BJ/def2-SVP and the change in $\Delta\Delta H^{\ddagger}$. Atom-atom distances for O2–H3 (black) and H3–O4 (red) and the average of the two values (blue) for each of cage **1–14** are shown. There is a poor correlation between $\Delta\Delta H^{\ddagger}$ and R_{H3-O4} for all cages, indicating the field effect does not predominantly work due to nucleophilic activation. For unfavorable fields, the broad, unsymmetrical bond length changes across O2,H3,O4 indicate somehwat greater rearrangement in the TS.

synergy between positive A and negative B. The combined stabilising effect of **A** and **B** in cage **2** ($\Delta\Delta H^{\ddagger}_{2} = -2.85$ kcal/mol) is greater by 0.5 kcal/mol than the sum of the individual effects of the charged groups on cage **6** and cage **8** ($\Delta\Delta H^{\ddagger}_{6+8} = -2.32$ kcal/mol).

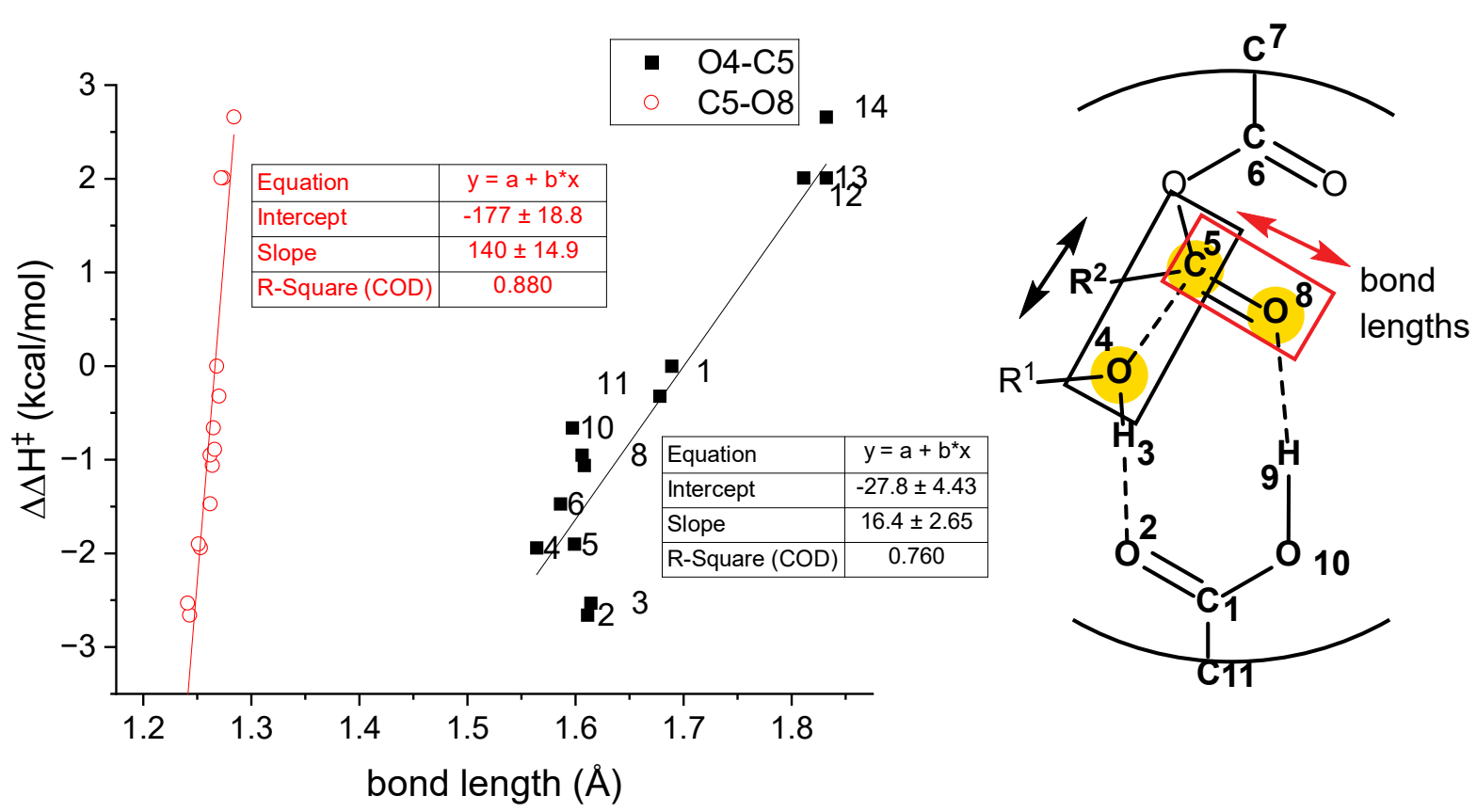

Figure S28: Parameters extracted from the transition states found in cages **1–14** using PBE0-D3BJ/def2-SVP and the change in $\Delta\Delta H^{\ddagger}$. Atom-atom distances for O4–C5 (black) and C5–O8 (red) for each of cage **1–14** are shown. There is a strong positive correlation between $\Delta\Delta H^{\ddagger}$ and R_{C5-O8} for all cages, indicating the field effect does not predominantly work by weakening the carbonyl bond of the electrophile. The strong correlation across a large bond-length change between $\Delta\Delta H^{\ddagger}$ and R_{O4-C5} strongly indicates the role of the field is to promote/stabilise the new oxygen-carbon bond.

Mass-weighted Bond Calculations In Figure 6 in the main text, the bond length differences in the transition states for cage **2** – cage **1** are shown as mass-weighted bond length changes between atoms a and b, x_{ab}:

$$x_{\text{ab}} = r \cdot \sqrt{\mu_{\text{rm}}} \tag{S4}$$

where r is the bond length in the transition state and μ_{rm} is the reduced mass of atoms a and b:

$$\mu_{\text{rm}} = \frac{m_{\text{a}} m_{\text{b}}}{m_{\text{a}} + m_{\text{b}}} \tag{S5}$$

Tables for the Geometrical Analysis of the TS Complex

Table S5: Computed activation enthalpies $\Delta\Delta H^{\ddagger}_{\text{PBE0}}$, and selected bond parameters in $TS^{\ddagger}_{1}$ for cages **1–14**. All $\Delta\Delta H^{\ddagger}$ values include zero-point energy (ZPE) corrections. Activation enthalpies were obtained from single-point calculations at the SMD($CHCl_3$)-PBE0-D3BJ/def2-SVP level of theory. Angles x-y-z with y central are notated with <.

Cage	$\Delta\Delta H^{\ddagger}_{\text{PBE0}}$	O8–H9 (Å)	H9–O10 (Å)	average O8–H9 H9–O10 (Å)	O8–H9–O10 < (°)	C1–C6 (Å)
1	0	1.208	1.199	1.204	174.78	5.716
2	-2.66	1.491	1.034	1.263	177.97	5.693
3	-2.53	1.514	1.028	1.271	177.1	5.721
4	-1.94	1.477	1.048	1.263	176.01	5.757
5	-1.9	1.434	1.053	1.244	177.38	5.678
6	-1.47	1.328	1.106	1.217	177.47	5.675
7	-1.06	1.329	1.106	1.218	176.63	5.687
8	-0.95	1.342	1.098	1.220	176.85	5.685
9	-0.89	1.195	1.212	1.204	173.91	5.704
10	-0.66	1.303	1.122	1.213	176.44	5.677
11	-0.32	1.221	1.188	1.205	174.78	5.729
12	2.01	1.105	1.333	1.219	174.01	5.769
13	2.01	1.097	1.345	1.221	172.92	5.749
14	2.66	1.048	1.448	1.248	172.04	5.791

Table S6: Computed activation enthalpies $\Delta\Delta H^{\ddagger}_{\text{PBE0}}$, and selected bond parameters in $TS^{\ddagger}_{1}$ for cages **1–14**. All $\Delta\Delta H^{\ddagger}$ values include zero-point energy (ZPE) corrections. Activation enthalpies were obtained from single-point calculations at the SMD($CHCl_3$)-PBE0-D3BJ/def2-SVP level of theory. Angles x-y-z with y central are notated with <.

Cage	$\Delta\Delta H^{\ddagger}_{\text{PBE0}}$	O2–H3 (Å)	H3–O4 (Å)	average O2–H3 H3–O4 (Å)	2-3-4 < (°)	O4–C5 (Å)	C5–O8 (Å)
1	0	1.379	1.067	1.223	172.38	1.689	1.268
2	-2.66	1.269	1.133	1.201	172.29	1.611	1.243
3	-2.53	1.269	1.133	1.201	172.72	1.614	1.241
4	-1.94	1.251	1.148	1.200	170.21	1.564	1.253
5	-1.9	1.256	1.145	1.201	172.18	1.599	1.251
6	-1.47	1.274	1.131	1.203	172.6	1.586	1.262
7	-1.06	1.266	1.139	1.203	172.44	1.608	1.264
8	-0.95	1.26	1.142	1.201	172.42	1.606	1.262
9	-0.89	1.434	1.047	1.241	169.85	1.711	1.266
10	-0.66	1.29	1.122	1.206	171.53	1.597	1.265
11	-0.32	1.342	1.088	1.215	172.48	1.678	1.27
12	2.01	1.44	1.042	1.241	172.52	1.811	1.274
13	2.01	1.484	1.028	1.256	170.72	1.832	1.272
14	2.66	1.492	1.027	1.260	170.66	1.832	1.284

S2.13 Orbital Energy Trends under External Electric Fields

To further explore the effect of applied electric fields on cage **1**, we analysed the energies of frontier molecular orbitals (FMOs) across a range of field strengths. Figure S29 displays the computed energies for the HOMO and nearby occupied orbitals (blue), as well as the LUMO and nearby unoccupied orbitals (green), as a function of field strength.

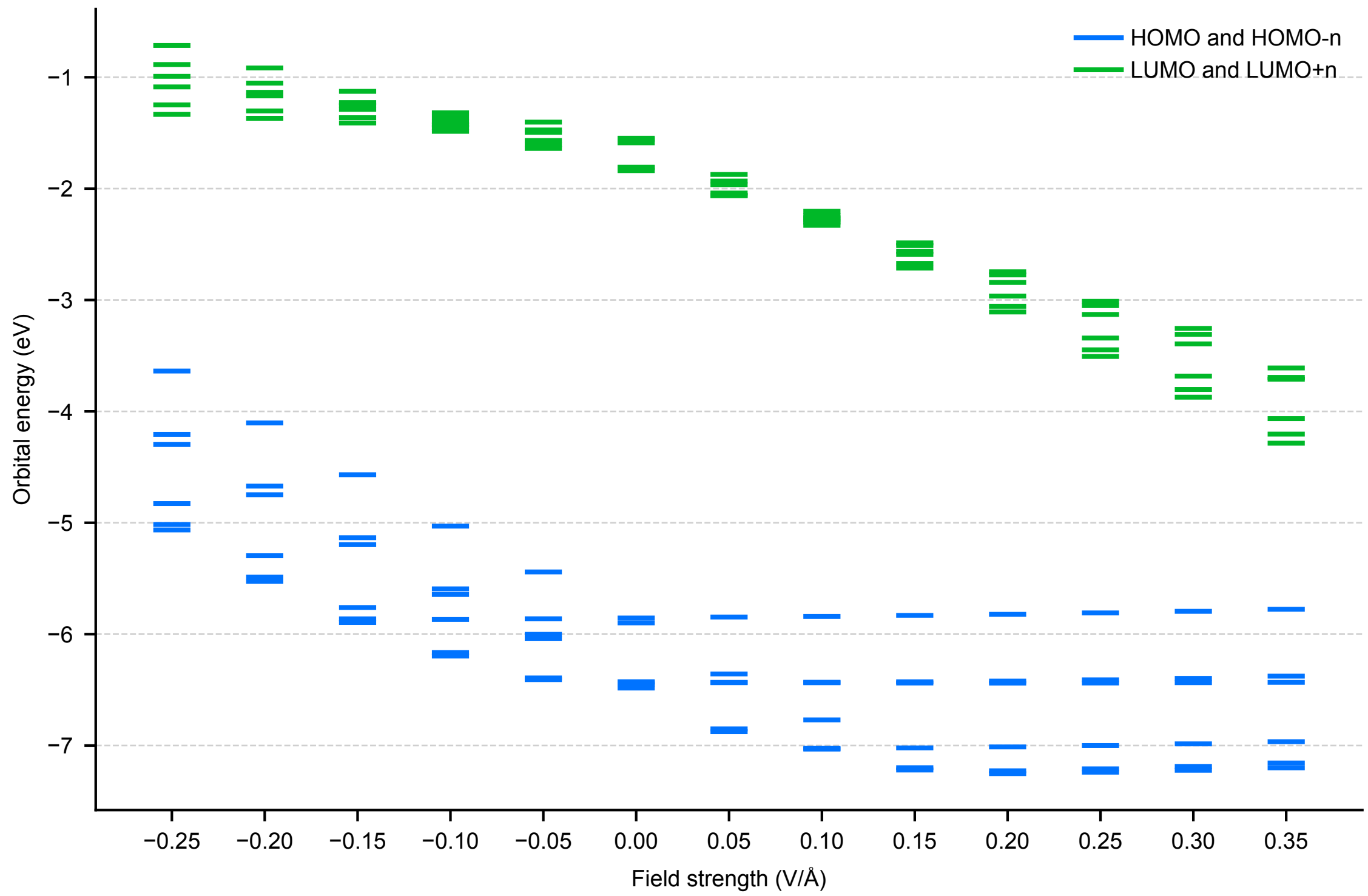

Figure S29: Orbital energies of cage **1** as a function of external electric field strength. Blue: HOMO and lower occupied orbitals; green: LUMO and higher unoccupied orbitals. All calculations at the SMD($CHCl_3$)-PBE0-D3BJ/def2-SVP level.

While we observe general trends in orbital stabilisation with increasing field strength, the figure does not reflect a consistent shift of the same orbital across the field series. Instead, both the HOMO and LUMO identities change as the field strength varies, indicating reordering of electronic structure. That is, the HOMO at −0.25 V/Å is not necessarily the same orbital (in spatial character or localisation) as the HOMO at −0.10 V/Å, and similarly for the LUMO.

This reordering reflects the complex polarisation of the electron density across the wider cage structure. As such, the direct use of frontier orbital energies as a descriptor for field-induced catalytic effects is unviable here: the FMOs involved at one field strength may be electronically or spatially distinct from those at another. Moreover, the catalysis occurs at a localised site between two functional groups, and these orbitals often extend well beyond this region.

Consequently, orbital energy diagrams do not capture the key effects driving catalysis under the applied field, which are thought to be the alignment of the activation dipole moment with the field vector and stabilisation of the transition state via charge redistribution. These quantities are more reliably probed using explicit field calculations of activation enthalpies and dipole-derived interaction energies, as described in the main text.

References

(1) Frisch, M. e.; Trucks, G.; Schlegel, H. B.; Scuseria, G.; Robb, M.; Cheeseman, J.; Scalmani, G.; Barone, V.; Petersson, G.; Nakatsuji, H., et al. Gaussian 16, 2016.

(2) Luchini, G.; Alegre-Requena, J.; Funes-Ardoiz, I.; Paton, R. *GoodVibes: automated thermochemistry for heterogeneous computational chemistry data [version 1; peer review: 2 approved with reservations]*. *F1000Research* **2020**, *9*,

(3) Andrews, K. G.; Piskorz, T. K.; Horton, P. N.; Coles, S. J. *Enzyme-like Acyl Transfer Catalysis in a Bifunctional Organic Cage*. *Journal of the American Chemical Society* **2024**, *146*, PMID: 38914009, 17887–17897,

(4) Adamo, C.; Barone, V. *Toward reliable density functional methods without adjustable parameters: The PBE0 model*. *The Journal of Chemical Physics* **1999**, *110*, 6158–6170,

(5) Weigend, F.; Ahlrichs, R. *Balanced basis sets of split valence, triple zeta valence and quadruple zeta valence quality for H to Rn: Design and assessment of accuracy*. *Phys. Chem. Chem. Phys.* **2005**, *7*, 3297–3305,

(6) Grimme, S.; Antony, J.; Ehrlich, S.; Krieg, H. *A consistent and accurate ab initio parametrization of density functional dispersion correction (DFT-D) for the 94 elements H-Pu*. *The Journal of Chemical Physics* **2010**, *132*, 154104,

(7) Grimme, S.; Ehrlich, S.; Goerigk, L. *Effect of the damping function in dispersion corrected density functional theory*. *Journal of Computational Chemistry* **2011**, *32*, 1456–1465,

(8) Marenich, A. V.; Cramer, C. J.; Truhlar, D. G. *Universal Solvation Model Based on Solute Electron Density and on a Continuum Model of the Solvent Defined by the Bulk Dielectric Constant and Atomic Surface Tensions*. *The Journal of Physical Chemistry B* **2009**, *113*, PMID: 19366259, 6378–6396,

(9) Stuyver, T.; Huang, J.; Mallick, D.; Danovich, D.; Shaik, S. *TITAN: A Code for Modeling and Generating Electric Fields—Features and Applications to Enzymatic Reactivity*. *Journal of Computational Chemistry* **2020**, *41*, 74–82,

(10) Li, L.; Li, C.; Zhang, Z.; Alexov, E. *On the Dielectric "Constant" of Proteins: Smooth Dielectric Function for Macromolecular Modeling and Its Implementation in DelPhi*. *Journal of Chemical Theory and Computation* **2013**, *9*, PMID: 23585741, 2126–2136,

(11) Riplinger, C.; Neese, F. *An efficient and near linear scaling pair natural orbital based local coupled cluster method*. *The Journal of Chemical Physics* **2013**, *138*, 034106,

(12) Neese, F.; Wennmohs, F.; Becker, U.; Riplinger, C. *The ORCA quantum chemistry program package*. *The Journal of Chemical Physics* **2020**, *152*, 224108,

(13) Dunning Thom H., J. *Gaussian basis sets for use in correlated molecular calculations. I. The atoms boron through neon and hydrogen*. *The Journal of Chemical Physics* **1989**, *90*, 1007–1023,

(14) Barone, V.; Cossi, M. *Quantum Calculation of Molecular Energies and Energy Gradients in Solution by a Conductor Solvent Model*. *The Journal of Physical Chemistry A* **1998**, *102*, 1995–2001,

(15) Wilcox, C. F.; Leung, C. *Transmission of substituent effects. Dominance of field effects*. *Journal of the American Chemical Society* **1968**, *90*, 336–341,

(16) Cole Thomas W., J.; Mayers, C. J.; Stock, L. M. *Chemistry of the bicyclo[2.2.2]octanes. XV. Dissociation constants of 4-substituted cubane-1-carboxylic acids. Evidence for the field model for the polar effect*. *Journal of the American Chemical Society* **1974**, *96*, 4555–4557,

(17) Bartlett, P. D.; Greene, F. D. *Triptycene 1-Carboxylic Acid and Related Compounds. The Decomposition of Ditriptoyl Peroxide*. *Journal of the American Chemical Society* **1954**, *76*, 1088–1096,

(18) Abraham, M. J.; Murtola, T.; Schulz, R.; Páll, S.; Smith, J. C.; Hess, B.; Lindahl, E. *GROMACS: High performance molecular simulations through multi-level parallelism from laptops to supercomputers*. *SoftwareX* **2015**, *1-2*, 19–25,

(19) Van Der Spoel, D.; Lindahl, E.; Hess, B.; Groenhof, G.; Mark, A. E.; Berendsen, H. J. C. *GROMACS: Fast, flexible, and free*. *Journal of Computational Chemistry* **2005**, *26*, 1701–1718,

(20) Bussi, G.; Donadio, D.; Parrinello, M. *Canonical sampling through velocity rescaling*. *The Journal of Chemical Physics* **2007**, *126*, 014101,

(21) Bernetti, M.; Bussi, G. *Pressure control using stochastic cell rescaling*. *The Journal of Chemical Physics* **2020**, *153*, 114107,

(22) Martí-Centelles, V.; Spicer, R. L.; Lusby, P. J. *Non-covalent allosteric regulation of capsule catalysis*. *Chemical Science* **2020**, *11*, 3236–3240,

(23) O'Connor, H. M.; Tipping, W. J.; Vallejo, J.; Nichol, G. S.; Faulds, K.; Graham, D.; Brechin, E. K.; Lusby, P. J. *Utilizing Raman Spectroscopy as a Tool for Solid- and Solution-Phase Analysis of Metalloorganic Cage Host–Guest Complexes*. *Inorganic Chemistry* **2022**, *62*, 1827–1832,

Main text reference annotations for:

Towards Next Generation Enzyme Mimics Through Local Electric Field Control in Organic Cage Catalysts

In order to facilitate the expeditious locating of supporting claims referenced in the main text, we include an annotated reference justification. The aim of the justification is to aid transparency, and save time for readers. The trail includes:

- details of which tables etc quoted values are taken from (Figures/Tables noted in the Annotation column refer to items in the referenced text);
- quotes from the original manuscripts to allow instant localisation of supporting claims using modern computer search functions;
- author comment on groups of references;
- longer footnotes;
- examples of counter-references disputing a claim made in the manuscript, where appropriate;

Main text manuscript citation quote	Annotation
**INTRODUCTION**	
Oriented electric fields (EFs) in enzyme active sites are increasingly recognised to enhance catalytic efficiency, primarily by stabilising the transition state (TS) dipole relative to the reactant-complex (RC) dipole (Figure 1a-c).[1–7]	Warshel has long championed the role of electrostatic catalysis in enzymes. [1,2]<br>Selection of reviews using EFs for enzyme design. [3–5]<br>Boxer has pioneered experimental studies. [6,7]
Although this "EF catalysis" is theoretically well-grounded using *external* fields[8–12] and in enzymes,[4,13,14]	Shaik and others have discussed and described EF theory and its trajectory recently.<br>Much of the notable work has focused on enzyme contexts. [4,13,14]
Further, most synthetic catalysts lack the architectural precision to robustly control *locally*-generated *through-space* EFs at the reaction site in solution, and rely on *through-bond* activation.[15]	Heschlag et all discuss the differences between small molecule catalysts and enzymes, including the role of binding:<br>"A fundamental difference between enzymes and small chemical catalysts is the ability of enzymes to use binding interactions with nonreactive portions of substrates to accelerate chemical reactions. Remote binding interactions can localise substrates to the active site, position substrates relative to enzymatic functional groups and other substrates, trigger conformational changes, induce local destabilisation, and modulate an active site environment by solvent exclusion." [15]<br><br>And: "Reactions of bound substrates can be facilitated by use of so-called "intrinsic binding energy", which can pay for substrate desolvation, distortion, *electrostatic destabilisation*, and entropy loss" (our emphasis). [15]
Therefore, molecular strategies to optimise catalysis using designed EFs remain underexplored,[4,16–28]	A significant number of reviews/perspectives,[19,20] computational studies,[16,18,21,24,26] and computational methods[17,27] concerning EFs in catalysis and design[25] have been recently reported.<br><br>Application of local electric fields to solution phase catalysis remains disproportionately underexplored.
in part because their potential remains unquantified.[29,30]	Coote and coworkers have reported theoretical progress towards molecular electrostatic catalysts. Unambiguous quantification of solution phase fields or demonstration of the precise effect of fields on catalytic barriers remains unreported.
Here, we describe a supramolecular approach to control and quantify *local* oriented EFs in chemical catalysis using charges arranged across the cavity of an organic cage enzyme mimic.[31,32]	Andrews has recently reported a hexapyridine cage,[32] and shown it performs acyl transfer catalysis.[31]
Enzymes constrain substrates with primary motifs, and tune strong EFs with secondary residues,[23] as in the case of lysozyme (Figure 1d).[33,34]	Oriented EF in lysozyme: secondary residues modulate the primary Asp52 and Glu35 active site motifs.[33,34] Lysozyme is a much studied enzyme with a polarising secondary field (see Fig 1bi)[33], although the field strength is, we believe, still unquantified.<br><br>Secondary spheres have been discussed.[23]

In this way, enzymes can favour stabilisation of the TS dipole despite the need for reactant complex stabilisation,[6,35]	Boxer has shown that values of K_M (a measure of substrate binding) remain relatively unchanged in mutants of KSI even in cases where k_{cat} changes by an order of magnitude. (e.g. Table 1)[6]<br><br>Head-Gordon reports a similar result in Table 1.[35]
with estimates of 72% of the catalytic barrier correlated with the EF in some cases.[6,36–38]	As much as 72% of the catalytic acceleration of ketosteroid isomerase has been attributed to its internal EF,[6,36–38]<br><br>Field strength for KSI: ensemble-average electric field of −144 ± 6 MV/cm.[36]<br><br>"This corresponds to an ~10^5-fold rate enhancement and accounts for 70% of KSI's catalytic speedup relative to an uncatalyzed reference reaction in solution."[36]<br><br>"Moreover, the electrostatic stabilisation energy is quite similar to the enthalpic component of KSI's barrier reduction (9 kcal mol^{-1}), suggesting that the thermodynamic manifestation of the electric field effect is enthalpic."[36]<br><br>"Using this value, and the experimental value of for wild-type KSI, the model in Figure 2a estimates a contribution to from electric field catalysis of 7.6 kcal mol−1, which is 72% of the total rate acceleration (k cat/k uncat = 4×10^7, 10.5 kcal mol−1)."[37]<br><br>We note that Herschlag has suggested that 70% is an overestimation, neglecting other effects (e.g. fig 2).[38]
In synthetic systems, unconstrained reactants will reorganise in EFs, forfeiting control of orientation,[10]	Shaik has noted that: "It is often said that a major problem for implementing the usage of OEEFs is the requirement to orient the reactants along the OEEF. However, this problem does not really exist since the OEEF itself will orient the molecular complex in space…"<br><br>However, it is unclear that the claim orientation is unrequired is valid in cases where differential stabilisation of reactant and transition states is required.
although general electrostatic effects[20,39–41]	Wheeler has discussed electrostatic interactions in catalysis. Coote has used pH dependent ions. Yang and Alexandrova have used tethered metal ions.
are still widely employed in catalysis.[42–50]	Ion pairs have been used to direct (template) catalysis, but the discussions rarely discuss electric field acceleration.<br>Zeolites are believed to benefit from strong EFs for catalysis.<br>Electro/photo catalysts are also important for harnessing EFs in the solid state.
Therefore, approaches to immobilise substrates to align them in EFs tuned by secondary sources have been investigated, with the goal of demonstrating EF-dependent catalysis.[4,16–30]	A significant number of reviews/perspectives,[19,20] computational studies,[16,18,21,24,26] and computational methods[17,27] concerning EFs in catalysis and design[25] have been recently reported.
Coote and others have immobilised reagents between electrodes that generate an oriented field to control catalysis in single molecule experiments (Figure 1d).[51–56]	Coote and coworkers have shown a ~5-fold rate enhancement of a Diels-Alder reaction between gold electrodes.<br><br>"Tapping data show that product formation increases by 4.4-fold, from 4.2% (252 product molecules out of 6,000 attempts) to 18.6% (1,116 product molecules out of 6,000 attempts) when the surface bias is changed from −0.05 V to −0.75 V"<br><br>Along with this seminal demonstration of a Diels-Alder reaction between electrodes, other single molecule examples are known, as well as varying creative use of electrodes.[54]<br>Rajamalli, Kaliginedi and co-workers have shown that a single "break junction" can promote a single molecular tautomerisation reaction in an applied field.[56]

Parallel plate capacitor systems employ frozen liquids to prevent solution-phase molecular reorganisation,[57–59]	This technique allows application of Stark spectroscopy to obtain significant data, including using frozen solvents:<br><br>See Fig 3A:[57] "In vibrational Stark spectroscopy, external electric fields are applied to a frozen isotropic sample, and the perturbation caused to the infrared spectrum is recorded. For many molecules, the response is linear and the experiment yields the linear Stark tuning rate."<br><br>A recent paper from the Boxer group on the vibrational Stark effect and influence of solvent. Frozen experiments are used, e.g. in Figure S7.[58]<br><br>A perspective article focussing on behaviour close to an electrode.[59]
and interfaces can orient fields and molecules.[60,61]	Electric fields can be harnessed at interfaces (orientation control and charge differences are possible).
Despite the effective use of electrostatics in cavity catalysis,[62–75]	A selection of admirable works with valuable supramolecular catalysts, and a useful monograph from Spicer and Lusby.[64]<br><br>Important supramolecular advances include charged metal-organic cages[65–71,75]<br><br>The capsule work of Gibb[72,73] and Tiefenbacher[63,74] is particularly relevant within all-organic systems.<br><br>For most charged cages, it is unclear how a dipolar field would be applied across a reaction, since the orientation of the reaction in the cage is variable, or difficult to predict or verify, and in any case the electrostatic field has no particular orientation (e.g. usually it is approximately monopolar).
alignment of local EFs over constrained reactants to quantify and enhance EF-catalysis in synthetic systems remains undeveloped.[76]	One approach was described by Shaik and co-workers in hypothetical computational work, to "add on" pendant carboxylate groups in a large porphyrin cage. The addition of these charged groups seems to affect the rate of chemistry at the porphyrin iron centers, although it remains unclear how the proposed complex low-symmetry structures in Fig S14 might be rationally designed, tuned, or synthetically accessed.
Here, we unite advances in EF theory[3,4,16–28]	A significant number of reviews/perspectives,[19,20] computational studies,[16,18,21,24,26] and computational methods[17,27] concerning EFs in catalysis and design[25] have been recently reported.
clarifying the results from enzymes,[6,15,36–38] and revealing design rules for synthetic EF catalysts.	See above – there are very few experimental measurements using enzymes, and no real unambiguous data from synthetic catalysis, and so the results are not generalised usefully yet. However, theoretical
To validate our conception, we explore analogues of a recently reported organic cage catalyst (Figure 2a).[31,32,77–79]	Andrews has recently reported a hexapyridine cage,[32] and shown it performs acyl transfer catalysis.[31] The cages can be readily modified, and have been termed robust organic cages (since they are covalently locked entities) to distinguish them from coordination cages and dynamically assembled imine cages, which are transiently present in solution.<br>Robust organic cages are reviewed here.[77]
**RESULTS AND DISCUSSION**	
Local EF catalysis in artificial molecular systems has been championed by Coote and Shaik in theoretical work.[16,29,30,39,76]	Application of local electric fields to solution phase catalysis remains disproportionately underexplored, even theoretically.<br><br>Coote has reported several approaches to harnessing charges in catalysis in small molecules (e.g. a pH switchable ion), including an example of experimental measurements of radical stability near different charges.[39]<br><br>One approach was described by Shaik and co-workers in hypothetical computational work, to "add on" pendant carboxylate groups in a (unsynthesised) porphyrin cage to create oriented

	fields. The addition of these charged groups seems to affect the rate of chemistry at the porphyrin iron centres. While informative, it remains unclear how the proposed complex low-symmetry structures (e.g. in Fig S14) might be rationally designed, tuned, or synthetically accessed, and the design does not discuss tuning general fields for application in solution.[76]
Neutral programmable organic cage catalyst **1**[31,32,77–79]	See above
Unoriented electrostatic catalysis in cages is well-explored,[62–75]	See above
catalyses organocatalytic acyl transfer using two endohedral[80] carboxylic acid groups	Endohedral (internal/internally facing functionality) cages have been recently reviewed by Otte, demonstrating the very few examples of endohedral cage catalysts reported.
This two-point interaction constrains the reaction axis (Figure 1b) with respect to the cage triptycenyl axis.[32]	Substrate binding was found to be about 1 kcal/mol, and the reaction requires a direct proton wire along the axis between the acid groups.
The acyl transfer step involves concerted proton transfer and so is expected to be electrostatically sensitive.[63,81,82]	See discussions on “proton wires”
Synthetic access to versions of cage **1** with different **A** and **B** triptycenes has already been achieved.[31]	See Figure 5 in [31]
Bridgehead substituted triptycenes (Figure 2b)[83–85] are well-described due to their importance in materials science.[86,87]	The building blocks and method of synthesis of the cage analogues in this paper are strongly precedented by recent work in triptycene development.
Large molecular cages such as **1** can use tunable side-chains (e.g. ^{t}Bu) for solubility or to sterically protect charged groups, or use large non-coordinating counterions, which makes accessibility and application of suitable variants in chlorinated solvents plausible.[67,75]	Lusby has used chlorinated solvents (e.g. dichloromethane) to dissolve charged (metal-organic) cages, for catalysis. Large non-coordinating counterions (e.g. BArF) can be used to enhance charge and electrostatic effects and avoid cavity quenching.
bridgehead-substituted bicyclo[2.2.2]octane-1-carboxylic acids, which are known to modulate the acid pK_a *through-space* (i.e. field effects)[88,89]	Cockcroft has recently demonstrated the role of “through-space” (field) effects using molecular balances;[88] field effects are well described, but not always recognised.[89]
rather than through 6-8 sigma bonds.[90,91]	Elegant experiments demonstrate the pK_a changes in bicyclo[2.2.2]octane-1-carboxylic acids are consistent with distance, and not the number of intervening bonds, between the acid and the field-modifying group.
In any case, chloroform (ϵ_r = 4.8) has a low dielectric constant, comparable to many protein models (ϵ_r = 4.0),[92]	“The most commonly used dielectric constant value is ε = 4, which is believed to account for electronic polarisation and small backbone fluctuations.”
Cage **1** strongly aligns the **A**-**B** charged groups with the “reaction axis”,[10]	See Shaik’s discussion: 1. The OEEF Concept: Its Story of Conception
since the **A-B** (z-) axis is conformationally constrained due to pyridine-amide interactions.[31,32,77]	See in particular discussion in section 2.2, here[32]
Molecular dynamics simulations of cage **1** (298 K, chloroform)[31]	Previously investigated here (see SI):[31]
The rate-enhancement over the solution-phase reaction ($k_{\text{cage 1}}/k_{\text{solvent}}$) exceeds 10^4.[31]	See table 1 for the initial 10^4.[31]<br>The additional two orders of magnitude is calculated by varying k in the Eyring equation.
We will briefly explain the pertinent theory of EF catalysis.[10,37]	There are many excellent accounts referenced through-out this manuscript.
Following Stuyver,[93] a constant external field, F_{app}, can be produced along the z-axis using a parallel plate capacitor.	The TITAN code is a convenient way to simulate charged plates in DFT calculations.
These fields could be reconstructed from partial charges,[94] but such an approach is not ideal as partial charges are not unambiguously defined.	

In previous work,[93] *external* fields, F_{app}, are usually represented with a virtually *infinite* plate capacitor model:	
and ε is the electric permittivity $\varepsilon_0\varepsilon_r$, and we set ε_r = 4.0.[92]	As above – models both enzymes and chloroform, the solvent used in experiments with neutral cage **1**.
This value of r yields computed field strengths, F_{sub}, of ~0.2 V/Å for the most active cages **2-5** (see Tables S2-3), only marginally lower than known enzyme fields (e.g. cyclophilin A previously estimated at 0.5 V/Å).[37,95]	"Cyclophilin A… … Using a typical value (3.5 D) for a peptide carbonyl dipole and a calculated estimate for the electric field magnitude… of 50 MV/cm."[37]<br><br>"Assuming that the average electrostatic field in the active site of cyclophilin A is 40 MV/cm."[95]<br><br>"we calculated the electrostatic field acting on the carbonyl group in three other structures representing the three major families of PPIases: immunophilins (including cyclophilins), FK506-binding proteins (FKBPs), and parvulins (3). Our results were consistent with those found for cyclophilin A: −19 MV/cm for cyclophilin B (PDB ID code 1VAI), −33 MV/cm for an FKBP (PDB ID code 4ITZ), and −10 MV/cm for Pin1 (PDB ID code 1PIN), a parvulin." [95]
Experimentally, the catalytic motifs (e.g. carbonyl groups) or bound substrate can act as a reporter for the field strength using concepts from Stark spectroscopy (i.e. measuring field-dependent IR shifts).[96]	Boxer describes how EFs can be measured using probes in local EFs.
In this case, prediction of the favorable field direction for catalysis aligns with intuition associated with promoting HOMO (Nu, O_4) → LUMO (E, C_5) interactions.[10]	In these complex species, the HOMO and LUMO are not necessarily on the Nu/E; we point to what organic chemists might intuitively guess in a simpler system. It is plausible that the reactant "nucleophile" is destabilised at the negative end of the EF, and stabilised by moving towards the positively charged end of the EF.<br><br>See Shaik (8. Applications of the Reaction Rule) for a discussion of intuitive EF alignment heuristics.[10]
**Methods**<br><br>All geometry optimisations were performed in the gas phase using Gaussian 16.[97] Zero-point energy (ZPE) corrections were obtained using GoodVibes,[98] and all reported thermochemical values include these corrections. Based on previous kinetic studies,[31] a unimolecular rate-determining step was assumed (reactant complex → transition structure), so no corrections for change in standard state were necessary. All structures used in the analysis were confirmed to be minima or first-order saddle points through harmonic frequency calculations. For DFT calculations, as previously reported for this system,[31] the PBE0 hybrid functional[99] was used in conjunction with Ahlrichs' def2-SVP basis set[100] and a damped D3 dispersion correction (D3BJ).[101,102] Solvent effects ($CHCl_3$) were modelled using the SMD implicit solvation model,[103] and included as a single-point correction to ΔH; for best- and worst-performing cages, similar results were obtained (Fig S10) by performing geometry optimisations and frequency calculations with an implicit solvent (C-PCM[104,105]). Explicit electric field calculations were performed by applying static fields, F_{app}, along the reaction axis. In these calculations, two bridgehead atoms of the triptycene framework were frozen to prevent rigid-body reorientation in the field. High-level single-point energies were calculated using DLPNO-CCSD(T)[106] as implemented in ORCA 5.0.3,[107,108] employing the cc-pVTZ basis set.[109] Solvent effects in these correlated calculations were included via the	Standard quantum chemistry references.<br><br>GoodVibes is a multifaceted python package to correct the poor description of low frequency vibrations by the rigid-rotor harmonic oscillator treatment.<br><br>The DFT methods used show good agreement with previous experimental data in previous work[31] and are validated for the changes in this system by reference to high level coupled cluster calculations.

conductor-like polarisable continuum model (C-PCM),[104,105] with chloroform as the solvent. Electrostatic potential (ESP) maps were generated using the orca_vpot utility in ORCA 5.0.3,[107,108] based on electron densities computed with the PBE0 functional.[99]	

REFERENCES

1. Warshel, A. Energetics of Enzyme Catalysis. *Proc. Natl. Acad. Sci. U. S. A.* **1978**, *75* (11), 5250–5254. https://doi.org/10.1073/PNAS.75.11.5250.
2. Warshel, A.; Sharma, P. K.; Kato, M.; Xiang, Y.; Liu, H.; Olsson, M. H. M. Electrostatic Basis for Enzyme Catalysis. *Chem. Rev.* **2006**, 3210–3235. https://doi.org/10.1021/cr0503106.
3. Chaturvedi, S. S.; Bím, D.; Christov, C. Z.; Alexandrova, A. N. From Random to Rational: Improving Enzyme Design through Electric Fields, Second Coordination Sphere Interactions, and Conformational Dynamics. *Chem. Sci.* **2023**, *14* (40), 10997–11011. https://doi.org/10.1039/D3SC02982D.
4. Siddiqui, S. A.; Stuyver, T.; Shaik, S.; Dubey, K. D. Designed Local Electric Fields─Promising Tools for Enzyme Engineering. *JACS Au* **2023**, *3* (12), 3259–3269. https://doi.org/10.1021/JACSAU.3C00536.
5. Ekesan, Ş.; McCarthy, E.; Case, D. A.; York, D. M. RNA Electrostatics: How Ribozymes Engineer Active Sites to Enable Catalysis. *J. Phys. Chem. B* **2022**, *126* (32), 5982–5990. https://doi.org/10.1021/ACS.JPCB.2C03727.
6. Wu, Y.; Boxer, S. G. A Critical Test of the Electrostatic Contribution to Catalysis with Noncanonical Amino Acids in Ketosteroid Isomerase. *J. Am. Chem. Soc.* **2016**, *138* (36), 11890–11895. https://doi.org/10.1021/JACS.6B06843.
7. Zheng, C.; Mao, Y.; Kozuch, J.; Atsango, A. O.; Ji, Z.; Markland, T. E.; Boxer, S. G. A Two-Directional Vibrational Probe Reveals Different Electric Field Orientations in Solution and an Enzyme Active Site. *Nat. Chem.* **2022**, *14* (8), 891–897. https://doi.org/10.1038/s41557-022-00937-w.
8. Léonard, N. G.; Dhaoui, R.; Chantarojsiri, T.; Yang, J. Y. Electric Fields in Catalysis: From Enzymes to Molecular Catalysts. *ACS Catal.* **2021**, *11* (17), 10923–10932. https://doi.org/10.1021/ACSCATAL.1C02084.
9. Che, F.; Gray, J. T.; Ha, S.; Kruse, N.; Scott, S. L.; McEwen, J. S. Elucidating the Roles of Electric Fields in Catalysis: A Perspective. *ACS Catal.* **2018**, *8* (6), 5153–5174. https://doi.org/10.1021/ACSCATAL.7B02899.
10. Shaik, S. My Vision of Electric-Field-Aided Chemistry in 2050. *ACS Phys. Chem. Au* **2024**, *4* (3), 191–201. https://doi.org/10.1021/ACSPHYSCHEMAU.3C00064.
11. Meir, R.; Chen, H.; Lai, W.; Shaik, S. Oriented Electric Fields Accelerate Diels–Alder Reactions and Control the Endo/Exo Selectivity. *ChemPhysChem* **2010**, *11* (1), 301–310. https://doi.org/10.1002/CPHC.200900848.
12. Shaik, S.; Mandal, D.; Ramanan, R. Oriented Electric Fields as Future Smart Reagents in Chemistry. *Nat. Chem.* **2016**, *8* (12), 1091–1098. https://doi.org/10.1038/nchem.2651.
13. de Visser, S. P.; Mukherjee, G.; Ali, H. S.; Sastri, C. V. Local Charge Distributions, Electric Dipole Moments, and Local Electric Fields Influence Reactivity Patterns and Guide Regioselectivities in α-Ketoglutarate-Dependent Non-Heme Iron Dioxygenases. *Acc. Chem. Res.* **2021**, *55* (1), 65–74. https://doi.org/10.1021/ACS.ACCOUNTS.1C00538.
14. Hanoian, P.; Liu, C. T.; Hammes-Schiffer, S.; Benkovic, S. Perspectives on Electrostatics and Conformational Motions in Enzyme Catalysis. *Acc. Chem. Res.* **2015**, *48* (2), 482–489. https://doi.org/10.1021/AR500390E.
15. Schwans, J. P.; Kraut, D. A.; Herschlag, D. Determining the Catalytic Role of Remote Substrate Binding Interactions in Ketosteroid Isomerase. *Proc. Natl. Acad. Sci. U. S. A.* **2009**, *106* (34), 14271–14275. https://doi.org/10.1073/PNAS.0901032106.
16. Blyth, M. T.; Noble, B. B.; Russell, I. C.; Coote, M. L. Oriented Internal Electrostatic Fields Cooperatively Promote Ground- And Excited-State Reactivity: A Case Study in Photochemical CO2 Capture. *J. Am. Chem. Soc.* **2020**, *142* (1), 606–613. https://doi.org/10.1021/JACS.9B12186.
17. Hanaway, D. J.; Kennedy, C. R. Automated Variable Electric-Field DFT Application for Evaluation of Optimally Oriented Electric Fields on Chemical Reactivity. *J. Org. Chem.* **2023**, *88* (1), 106–115. https://doi.org/10.1021/ACS.JOC.2C01893.
18. Yu, S.; Vermeeren, P.; Hamlin, T. A.; Bickelhaupt, F. M. How Oriented External Electric Fields Modulate Reactivity. *Chem. - Eur. J.* **2021**, *27* (18), 5683–5693. https://doi.org/10.1002/CHEM.202004906.
19. Li, W. L.; Head-Gordon, T. Catalytic Principles from Natural Enzymes and Translational Design Strategies for Synthetic Catalysts. *ACS Cent. Sci.* **2021**, *7* (1), 72–80. https://doi.org/10.1021/ACSCENTSCI.0C01556.
20. Maji, R.; Mallojjala, S. C.; Wheeler, S. E. Electrostatic Interactions in Asymmetric Organocatalysis. *Acc. Chem. Res.* **2023**, *56* (14), 1990–2000. https://doi.org/10.1021/acs.accounts.3c00198.
21. Wheeler, S. E.; Houk, K. N. Through-Space Effects of Substituents Dominate Molecular Electrostatic Potentials of Substituted Arenes. *J. Chem. Theory Comput.* **2009**, *5* (9), 2301–2312. https://doi.org/10.1021/CT900344G.
22. Maji, R.; Wheeler, S. E. Importance of Electrostatic Effects in the Stereoselectivity of NHC-Catalyzed Kinetic Resolutions. *J. Am. Chem. Soc.* **2017**, *139* (36),

12441–12449. https://doi.org/10.1021/JACS.7B01796.
23. Dhayalan, V.; Gadekar, S. C.; Alassad, Z.; Milo, A. Unravelling Mechanistic Features of Organocatalysis with in Situ Modifications at the Secondary Sphere. *Nat. Chem.* **2019**, *11* (6), 543–551. https://doi.org/10.1038/s41557-019-0258-1.
24. Neel, A. J.; Hilton, M. J.; Sigman, M. S.; Toste, F. D. Exploiting Non-Covalent π Interactions for Catalyst Design. *Nature* **2017**, *543* (7647), 637–646. https://doi.org/10.1038/nature21701.
25. Knowles, R. R.; Jacobsen, E. N. Attractive Noncovalent Interactions in Asymmetric Catalysis: Links between Enzymes and Small Molecule Catalysts. *Proc. Natl. Acad. Sci. U. S. A.* **2010**, *107* (48), 20678–20685. https://doi.org/10.1073/PNAS.1006402107.
26. Welborn, V. V.; Ruiz Pestana, L.; Head-Gordon, T. Computational Optimization of Electric Fields for Better Catalysis Design. *Nat. Catal.* **2018**, *1* (9), 649–655. https://doi.org/10.1038/s41929-018-0109-2.
27. Besalú-Sala, P.; Solà, M.; Luis, J. M.; Torrent-Sucarrat, M. Fast and Simple Evaluation of the Catalysis and Selectivity Induced by External Electric Fields. *ACS Catal.* **2021**, *11* (23), 14467–14479. https://doi.org/10.1021/ACSCATAL.1C04247.
28. Shaik, S.; Ramanan, R.; Danovich, D.; Mandal, D. Structure and Reactivity/Selectivity Control by Oriented-External Electric Fields. *Chem. Soc. Rev.* **2018**, *47* (14), 5125–5145. https://doi.org/10.1039/C8CS00354H.
29. Hill, N. S.; Coote, M. L. Internal Oriented Electric Fields as a Strategy for Selectively Modifying Photochemical Reactivity. *J. Am. Chem. Soc.* **2018**, *140* (50), 17800–17804. https://doi.org/10.1021/JACS.8B12009.
30. Blyth, M. T.; Coote, M. L. A PH-Switchable Electrostatic Catalyst for the Diels-Alder Reaction: Progress toward Synthetically Viable Electrostatic Catalysis. *J. Org. Chem.* **2019**, *84* (3), 1517–1522. https://doi.org/10.1021/ACS.JOC.8B02940.
31. Andrews, K. G.; Piskorz, T. K.; Horton, P. N.; Coles, S. J. Enzyme-like Acyl Transfer Catalysis in a Bifunctional Organic Cage. *J. Am. Chem. Soc.* **2024**, *146* (26), 17887–17897. https://doi.org/10.1021/jacs.4c03560.
32. Andrews, K. G.; Horton, P. N.; Coles, S. J. Programmable Synthesis of Organic Cages with Reduced Symmetry. *Chem. Sci.* **2024**, *15* (17), 6536–6543. https://doi.org/10.1039/D4SC00889H.
33. Sun, D. P.; Liao, D. I.; Remington, S. J. Electrostatic Fields in the Active Sites of Lysozymes. *Proc. Natl. Acad. Sci. U. S. A.* **1989**, *86* (14), 5361–5365. https://doi.org/10.1073/pnas.86.14.5361.
34. Tanaka, I.; Nishinomiya, R.; Goto, R.; Shimazakic, S.; Chataked, T. Recent Structural Insights into the Mechanism of Lysozyme Hydrolysis. *IUCrJ* **2021**, *77* (3), 288–292. https://doi.org/10.1107/S2059798321000346.
35. Vaissier, V.; Sharma, S. C.; Schaettle, K.; Zhang, T.; Head-Gordon, T. Computational Optimization of Electric Fields for Improving Catalysis of a Designed Kemp Eliminase. *ACS Catal.* **2018**, *8* (1), 219–227. https://doi.org/10.1021/ACSCATAL.7B03151.
36. Fried, S. D.; Bagchi, S.; Boxer, S. G. Extreme Electric Fields Power Catalysis in the Active Site of Ketosteroid Isomerase. *Science* **2014**, *346* (6216), 1510–1514. https://doi.org/10.1126/SCIENCE.1259802.
37. Fried, S. D.; Boxer, S. G. Electric Fields and Enzyme Catalysis. *Annu. Rev. Biochem.* **2017**, *86*, 387–415. https://doi.org/10.1146/annurev-biochem-061516-044432.
38. Natarajan, A.; Yabukarski, F.; Lamba, V.; Schwans, J. P.; Sunden, F.; Herschlag, D. Comment on "Extreme Electric Fields Power Catalysis in the Active Site of Ketosteroid Isomerase." *Science* **2015**, *349* (6251), 936a. https://doi.org/10.1126/SCIENCE.AAB1584.
39. Klinska, M.; Smith, L. M.; Gryn'ova, G.; Banwell, M. G.; Coote, M. L. Experimental Demonstration of PH-Dependent Electrostatic Catalysis of Radical Reactions. *Chem. Sci.* **2015**, *6* (10), 5623–5627. https://doi.org/10.1039/C5SC01307K.
40. Kang, K.; Fuller, J.; Reath, A. H.; Ziller, J. W.; Alexandrova, A. N.; Yang, J. Y. Installation of Internal Electric Fields by Non-Redox Active Cations in Transition Metal Complexes. *Chem. Sci.* **2019**, *10* (43), 10135–10142. https://doi.org/10.1039/C9SC02870F.
41. Martin, D. J.; Mayer, J. M. Oriented Electrostatic Effects on O2 and CO2 Reduction by a Polycationic Iron Porphyrin. *J. Am. Chem. Soc.* **2021**, *143* (30), 11423–11434. https://doi.org/10.1021/JACS.1C03132.
42. Breslow, R.; Heyer, D. Directed Steroid Chlorination Catalyzed by an Ion-Paired Template. *Tetrahedron Lett.* **1983**, *24* (46), 5039–5042. https://doi.org/10.1016/S0040-4039(00)94035-8.
43. Chattopadhyay, B.; Dannatt, J. E.; Andujar-De Sanctis, I. L.; Gore, K. A.; Maleczka, R. E.; Singleton, D. A.; Smith, M. R. Ir-Catalyzed Ortho-Borylation of Phenols Directed by Substrate-Ligand Electrostatic Interactions: A Combined Experimental/in Silico Strategy for Optimizing Weak Interactions. *J. Am. Chem. Soc.* **2017**, *139* (23), 7864–7871. https://doi.org/10.1021/JACS.7B02232.
44. Ye, J.; Kalvet, I.; Schoenebeck, F.; Rovis, T. Direct α-Alkylation of Primary Aliphatic Amines Enabled by CO2 and Electrostatics. *Nat. Chem.* **2018**, *10* (10), 1037–1041. https://doi.org/10.1038/s41557-018-0085-9.
45. Genov, G. R.; Douthwaite, J. L.; Lahdenperä, A. S. K.; Gibson, D. C.; Phipps, R. J. Enantioselective Remote C-H Activation Directed by a Chiral Cation. *Science* **2020**, *367* (6483), 1246–1251. https://doi.org/10.1126/science.aba1120.
46. Toste, F. D.; Sigman, M. S.; Miller, S. J. Pursuit of Noncovalent Interactions for Strategic Site-Selective Catalysis. *Acc. Chem. Res.* **2017**, *50* (3), 609–615. https://doi.org/10.1021/ACS.ACCOUNTS.6B00613.
47. Brak, K.; Jacobsen, E. N.; Jacobsen, E. N.; Brak, K. Asymmetric Ion-Pairing Catalysis. *Angew. Chem., Int.*

Ed. **2013**, *52* (2), 534–561. https://doi.org/10.1002/ANIE.201205449.
48. Rhodes, C. J. Electric Fields in Zeolites: Fundamental Features and Environmental Implications. *Chem. Pap.* **2016**, *70* (1), 4–21. https://doi.org/10.1515/CHEMPAP-2015-0160.
49. Chen, L.; Ren, J. T.; Yuan, Z. Y. Enabling Internal Electric Fields to Enhance Energy and Environmental Catalysis. *Adv. Energy Mater.* **2023**, *13* (11), 2203720. https://doi.org/10.1002/AENM.202203720.
50. Liu, N.; Zhang, R.; Li, Y.; Chen, B. Local Electric Field Effect of TMI (Fe, Co, Cu)-BEA on N2O Direct Dissociation. *J. Phys. Chem. C* **2014**, *118* (20), 10944–10956. https://doi.org/10.1021/JP5023949.
51. Aragonès, A. C.; Haworth, N. L.; Darwish, N.; Ciampi, S.; Bloomfield, N. J.; Wallace, G. G.; Diez-Perez, I.; Coote, M. L. Electrostatic Catalysis of a Diels–Alder Reaction. *Nature* **2016**, *531* (7592), 88–91. https://doi.org/10.1038/nature16989.
52. Huang, X.; Tang, C.; Li, J.; Chen, L. C.; Zheng, J.; Zhang, P.; Le, J.; Li, R.; Li, X.; Liu, J.; Yang, Y.; Shi, J.; Chen, Z.; Bai, M.; Zhang, H. L.; Xia, H.; Cheng, J.; Tian, Z. Q.; Hong, W. Electric Field–Induced Selective Catalysis of Single-Molecule Reaction. *Sci. Adv.* **2019**, *5* (6), e3072–e3093. https://doi.org/10.1126/SCIADV.AAW3072.
53. Lv, J.; Sun, R.; Yang, Q.; Gan, P.; Yu, S.; Tan, Z. Research on Electric Field—Induced Catalysis Using Single—Molecule Electrical Measurement. *Molecules* **2023**, *28* (13), 4968. https://doi.org/10.3390/MOLECULES28134968.
54. López, M. Á. G.; Ali, R.; Tan, M. L.; Sakai, N.; Wirth, T.; Matile, S. Electric Field–Assisted Anion-π Catalysis on Carbon Nanotubes in Electrochemical Microfluidic Devices. *Sci Adv* **2023**, *9*, eadj5502. https://doi.org/10.1126/SCIADV.ADJ5502.
55. Borca, B.; Michnowicz, T.; Pétuya, R.; Pristl, M.; Schendel, V.; Pentegov, I.; Kraft, U.; Klauk, H.; Wahl, P.; Gutzler, R.; Arnau, A.; Schlickum, U.; Kern, K. Electric-Field-Driven Direct Desulfurization. *ACS Nano* **2017**, *11* (5), 4703–4709. https://doi.org/10.1021/ACSNANO.7B00612.
56. Sreelakshmi, P. A.; Mahashaya, R.; Leitherer, S.; Rashid, U.; Hamill, J. M.; Nair, M.; Rajamalli, P.; Kaliginedi, V. Electric Field-Induced Sequential Prototropic Tautomerism in Enzyme-like Nanopocket Created by Single Molecular Break Junction. *J. Am. Chem. Soc.* **2024**, *146* (51), 35242–35251. https://doi.org/10.1021/JACS.4C12423.
57. Fried, S. D.; Boxer, S. G. Measuring Electric Fields and Noncovalent Interactions Using the Vibrational Stark Effect. *Acc. Chem. Res.* **2015**, *48* (4), 998–1006. https://doi.org/10.1021/AR500464J.
58. Zheng, C.; Mao, Y.; Markland, T. E.; Boxer, S. G. Beyond the Vibrational Stark Effect: Unraveling the Large Redshifts of Alkyne C-H Bond in Solvation Environments. *J. Am. Chem. Soc.* **2025**, *147* (7), 6227–6235. https://doi.org/10.1021/JACS.4C18102.
59. Bhattacharyya, D.; Videla, P. E.; Cattaneo, M.; Batista, V. S.; Lian, T.; Kubiak, C. P. Vibrational Stark Shift Spectroscopy of Catalysts under the Influence of Electric Fields at Electrode–Solution Interfaces. *Chem. Sci.* **2021**, *12* (30), 10131–10149. https://doi.org/10.1039/D1SC01876K.
60. Gorin, C. F.; Beh, E. S.; Bui, Q. M.; Dick, G. R.; Kanan, M. W. Interfacial Electric Field Effects on a Carbene Reaction Catalyzed by Rh Porphyrins. *J. Am. Chem. Soc.* **2013**, *135* (30), 11257–11265. https://doi.org/10.1021/JA404394Z.
61. Zhang, L.; Vogel, Y. B.; Noble, B. B.; Gonçales, V. R.; Darwish, N.; Brun, A. Le; Gooding, J. J.; Wallace, G. G.; Coote, M. L.; Ciampi, S. TEMPO Monolayers on Si(100) Electrodes: Electrostatic Effects by the Electrolyte and Semiconductor Space-Charge on the Electroactivity of a Persistent Radical. *J. Am. Chem. Soc.* **2016**, *138* (30), 9611–9619. https://doi.org/10.1021/JACS.6B04788.
62. Hastings, C. J.; Pluth, M. D.; Bergman, R. G.; Raymond, K. N. Enzymelike Catalysis of the Nazarov Cyclization by Supramolecular Encapsulation. *J. Am. Chem. Soc.* **2010**, *132* (20), 6938–6940. https://doi.org/10.1021/ja102633e.
63. Li, T. R.; Huck, F.; Piccini, G. M.; Tiefenbacher, K. Mimicry of the Proton Wire Mechanism of Enzymes inside a Supramolecular Capsule Enables β-Selective O-Glycosylations. *Nat. Chem.* **2022**, *14* (9), 985–994. https://doi.org/10.1038/s41557-022-00981-6.
64. Spicer, R. L.; Lusby, P. J. Chapter 2: Catalytic Strategies within the Confined Spaces of Coordination Cages. In *Monographs in Supramolecular Chemistry*; Royal Society of Chemistry, 2021; Vol. **2021-Janua**, pp 29–69. https://doi.org/10.1039/9781788019705-00029.
65. Martí-Centelles, V.; Lawrence, A. L.; Lusby, P. J. High Activity and Efficient Turnover by a Simple, Self-Assembled "Artificial Diels-Alderase." *J. Am. Chem. Soc.* **2018**, *140* (8), 2862–2868. https://doi.org/10.1021/JACS.7B12146.
66. Piskorz, T. K.; Martí-Centelles, V.; Young, T. A.; Lusby, P. J.; Duarte, F. Computational Modeling of Supramolecular Metallo-Organic Cages-Challenges and Opportunities. *ACS Catal.* **2022**, *12* (10), 5806–5826. https://doi.org/10.1021/acscatal.2c00837.
67. Zhou, H.; Piskorz, T. K.; Liu, K.; Lu, Y.; Duarte, F.; Lusby, P. J. Dissecting the Effects of Cage Structure in the Catalytic Activation of Imide Chlorenium-Ion Donors. *J. Am. Chem. Soc.* **2025**, *147* (13), 11456–11464 https://doi.org/10.1021/JACS.5C01249.
68. Pluth, M. D.; Bergman, R. G.; Raymond, K. H. Acid Catalysis in Basic Solution: A Supramolecular Host Promotes Orthoformate Hydrolysis. *Science* **2007**, *316* (5821), 85–88. https://doi.org/10.1126/SCIENCE.1138748.
69. Welborn, V. V.; Li, W. L.; Head-Gordon, T. Interplay of Water and a Supramolecular Capsule for Catalysis of Reductive Elimination Reaction from Gold. *Nat. Commun.* **2020**, *11*, 415. https://doi.org/10.1038/s41467-019-14251-6.
70. Welborn, V. V.; Head-Gordon, T. Electrostatics Generated by a Supramolecular Capsule Stabilizes the Transition State for Carbon-Carbon Reductive

Elimination from Gold(III) Complex. *J. Phys. Chem. Lett.* **2018**, *9* (14), 3814–3818. https://doi.org/10.1021/ACS.JPCLETT.8B01710.
71. Frushicheva, M. P.; Mukherjee, S.; Warshel, A. Electrostatic Origin of the Catalytic Effect of a Supramolecular Host Catalyst. *J. Phys. Chem. B* **2012**, *116* (45), 13353–13360. https://doi.org/10.1021/JP3084327.
72. Wang, K.; Cai, X.; Yao, W.; Tang, D.; Kataria, R.; Ashbaugh, H. S.; Byers, L. D.; Gibb, B. C. Electrostatic Control of Macrocyclization Reactions within Nanospaces. *J. Am. Chem. Soc.* **2019**, *141* (16), 6740–6747. https://doi.org/10.1021/JACS.9B02287.
73. Yao, W.; Wang, K.; Ismaiel, Y. A.; Wang, R.; Cai, X.; Teeler, M.; Gibb, B. C. Electrostatic Potential Field Effects on Amine Macrocyclizations within Yoctoliter Spaces: Supramolecular Electron Withdrawing/Donating Groups. *J. Phys. Chem. B* **2021**, *125* (32), 9333–9340. https://doi.org/10.1021/ACS.JPCB.1C05238.
74. Li, T. R.; Das, C.; Piccini, G. M.; Tiefenbacher, K. Tetrafluororesorcin[4]Arene Hexameric Capsule Enables the Expansion of the Reactivity Space in Supramolecular Catalysis. *J. Am. Chem. Soc.* **2025**, *147*, 11108–11116. https://doi.org/10.1021/JACS.4C17029.
75. Wang, J.; Young, T. A.; Duarte, F.; Lusby, P. J. Synergistic Noncovalent Catalysis Facilitates Base-Free Michael Addition. *J. Am. Chem. Soc.* **2020**, *142* (41), 17743–17750. https://doi.org/10.1021/jacs.0c08639.
76. Siddiqui, S. A.; Shaik, S.; Kalita, S.; Dubey, K. D. A Porphyrin-Based Molecular Cage Guided by Designed Local-Electric Field Is Highly Selective and Efficient. *Chem. Sci.* **2023**, *14* (37), 10329–10339. https://doi.org/10.1039/D3SC01720F.
77. Andrews, K. G. Beyond Symmetric Self-Assembly and Effective Molarity: Unlocking Functional Enzyme Mimics with Robust Organic Cages. *Beilstein J. Org. Chem.* **2025**, *21* (1), 421–443. https://doi.org/10.3762/BJOC.21.30.
78. Andrews, K. G.; Christensen, K. E. Access to Amide-linked Organic Cages by in Situ Trapping of Metastable Imine Assemblies: Solution Phase Bisamine Recognition. *Chem. - Eur. J.* **2023**, e202300063. https://doi.org/10.1002/chem.202300063.
79. Shields, C. E.; Fellowes, T.; Slater, A. G.; Cooper, A.; Andrews, K. G.; Szczypiński, F. T. Exploration of the Polymorphic Solid-State Landscape of an Amide-Linked Organic Cage Using Computation and Automation. *Chem. Commun.* **2024**, *60* (47), 6023–6026. https://doi.org/10.1039/D4CC01407C.
80. Otte, M. Reactions in Endohedral Functionalized Cages. *Eur. J. Org. Chem.* **2023**, *26* (18), e202300012. https://doi.org/10.1002/ejoc.202300012.
81. Cui, Q.; Karplus, M. Is a "Proton Wire" Concerted or Stepwise? A Model Study of Proton Transfer in Carbonic Anhydrase. *J. Phys. Chem. B* **2003**, *107* (4), 1071–1078. https://doi.org/10.1021/JP021931V.
82. Burykin, A.; Warshel, A. What Really Prevents Proton Transport through Aquaporin? Charge Self-Energy versus Proton Wire Proposals. *Biophys. J.* **2003**, *85* (6), 3696-3706. https://doi.org/10.1016/S0006-3495(03)74786-9.
83. Barros, S. A.; Yoon, I.; Suh, S. E.; Chenoweth, D. M. Bridgehead-Substituted Triptycenes for Discovery of Nucleic Acid Junction Binders. *Org. Lett.* **2016**, *18* (10), 2423–2426. https://doi.org/10.1021/acs.orglett.6b00945.
84. Wittig, G.; Steinhoff, G. Azatriptycen. *Justus Liebigs Ann. Chem.* **1964**, *676* (1), 21–31. https://doi.org/10.1002/JLAC.19646760104.
85. Chardon, A.; Osi, A.; Mahaut, D.; Doan, T. H.; Tumanov, N.; Wouters, J.; Fusaro, L.; Champagne, B.; Berionni, G. Controlled Generation of 9-Boratriptycene by Lewis Adduct Dissociation: Accessing a Non-Planar Triarylborane. *Angew. Chem. Int. Ed.* **2020**, *59* (30), 12402–12406. https://doi.org/10.1002/ANIE.202003119.
86. Ishiwari, F.; Shoji, Y.; Martin, C. J.; Fukushima, T. Recent Advances in Structurally Elaborate Triptycenes, Triptycene-Containing Polymers and Assemblies: Structures, Functions and Applications. *Polymer Journal 2024* **2024**, 1–28. https://doi.org/10.1038/s41428-024-00920-x.
87. Khan, Md. N.; Wirth, T. Chiral Triptycenes: Concepts, Progress and Prospects. *Chemistry – A European Journal* **2021**, chem.202005317. https://doi.org/10.1002/chem.202005317.
88. Burns, R. J.; Mati, I. K.; Muchowska, K. B.; Adam, C.; Cockroft, S. L. Quantifying Through-Space Substituent Effects. *Angew. Chem., Int. Ed.* **2020**, *59* (38), 16717–16724. https://doi.org/10.1002/ANIE.202006943.
89. Gardner Swain, C.; Lupton, E. C. Field and Resonance Components of Substituent Effects. *J. Am. Chem. Soc.* **1968**, *90* (16), 4328–4337. https://doi.org/10.1021/JA01018A024.
90. Wilcox, C. F.; Leung, C. Transmission of Substituent Effects. Dominance of Field Effects. *J. Am. Chem. Soc.* **1968**, *90* (2), 336–341. https://doi.org/10.1021/ja01004a023.
91. Cole, T. W.; Mayers, C. J.; Stock, L. M. Chemistry of the bicyclo[2.2.2]octanes. XV. Dissociation constants of 4-substituted cubane-1-carboxylic acids. Evidence for the field model for the polar effect. *J. Am. Chem. Soc.* **1974**, *96* (14), 4555–4557. https://doi.org/10.1021/JA00821A032.
92. Li, L.; Li, C.; Zhang, Z.; Alexov, E. On the Dielectric "Constant" of Proteins: Smooth Dielectric Function for Macromolecular Modeling and Its Implementation in DelPhi. *J. Chem. Theory Comput.* **2013**, *9* (4), 2126–2136. https://doi.org/10.1021/CT400065J.
93. Stuyver, T.; Huang, J.; Mallick, D.; Danovich, D.; Shaik, S. TITAN: A Code for Modeling and Generating Electric Fields—Features and Applications to Enzymatic Reactivity. *J. Comput. Chem.* **2020**, *41* (1), 74–82. https://doi.org/10.1002/JCC.26072.
94. Polêto, M. D.; Lemkul, J. A. TUPÃ: Electric Field Analyses for Molecular Simulations. *J. Comput. Chem.*

2022, *43* (16), 1113–1119. https://doi.org/10.1002/jcc.26873.
95. Camilloni, C.; Sahakyan, A. B.; Holliday, M. J.; Isern, N. G.; Zhang, F.; Eisenmesser, E. Z.; Vendruscolo, M. Cyclophilin a Catalyzes Proline Isomerization by an Electrostatic Handle Mechanism. *Proc. Natl. Acad. Sci. U. S. A.* **2014**, *111* (28), 10203–10208. https://doi.org/10.1073/PNAS.1404220111.
96. Schneider, S. H.; Boxer, S. G. Vibrational Stark Effects of Carbonyl Probes Applied to Reinterpret IR and Raman Data for Enzyme Inhibitors in Terms of Electric Fields at the Active Site. *J. Phys. Chem. B* **2016**, *120* (36), 9672–9684. https://doi.org/10.1021/ACS.JPCB.6B08133.
97. Frisch, M. J.; Trucks, G. W.; Schlegel, H. B.; Scuseria, G. E.; Robb, M. A.; Cheeseman, J. R.; Scalmani, G.; Barone, V.; Petersson, G. A.; Nakatsuji, H.; Li, X.; Caricato, M.; Marenich, A. V.; Bloino, J.; Janesko, B. G.; Gomperts, R.; Mennucci, B.; Hratchian, H. P.; Ortiz, J. V.; Izmaylov, A. F.; Sonnenberg, J. L.; Williams-Young, D.; Ding, F.; Lipparini, F.; Egidi, F.; Goings, J.; Peng, B.; Petrone, A.; Henderson, T.; Ranasinghe, D.; Zakrzewski, V. G.; Gao, J.; Rega, N.; Zheng, G.; Liang, W.; Hada, M.; Ehara, M.; Toyota, K.; Fukuda, R.; Hasegawa, J.; Ishida, M.; Nakajima, T.; Honda, Y.; Kitao, O.; Nakai, H.; Vreven, T.; Throssell, K.; Montgomery, J. A., Jr.; Peralta, J. E.; Ogliaro, F.; Bearpark, M. J.; Heyd, J. J.; Brothers, E. N.; Kudin, K. N.; Staroverov, V. N.; Keith, T. A.; Kobayashi, R.; Normand, J.; Raghavachari, K.; Rendell, A. P.; Burant, J. C.; Iyengar, S. S.; Tomasi, J.; Cossi, M.; Millam, J. M.; Klene, M.; Adamo, C.; Cammi, R.; Ochterski, J. W.; Martin, R. L.; Morokuma, K.; Farkas, O.; Foresman, J. B.; Fox, D. J. Gaussian 16, Revision C.01, Gaussian, Inc.: Wallingford CT, 2016.There is no corresponding record for this reference.
98. Luchini, G.; Alegre-Requena, J. V; Funes-Ardoiz, I.; Paton, R. S. GoodVibes: Automated Thermochemistry for Heterogeneous Computational Chemistry Data. *F1000Res* **2020**, *9*, 291. https://doi.org/10.12688/f1000research.22758.1.
99. Adamo, C.; Barone, V. Toward Reliable Density Functional Methods without Adjustable Parameters: The PBE0 Model. Journal of Chemical Physics **1999**, *110* (13), 6158–6170. https://doi.org/10.1063/1.478522.
100. Weigend, F.; Ahlrichs, R. Balanced Basis Sets of Split Valence, Triple Zeta Valence and Quadruple Zeta Valence Quality for H to Rn: Design and Assessment of Accuracy. *Physical Chemistry Chemical Physics* **2005**, *7* (18), 3297-3305. https://doi.org/10.1039/b508541a.
101. Grimme, S.; Antony, J.; Ehrlich, S.; Krieg, H. A Consistent and Accurate Ab Initio Parametrization of Density Functional Dispersion Correction (DFT-D) for the 94 Elements H-Pu. *Journal of Chemical Physics* **2010**, *132* (15), 154104. https://doi.org/10.1063/1.3382344.
102. Grimme, S.; Ehrlich, S.; Goerigk, L. Effect of the Damping Function in Dispersion Corrected Density Functional Theory. *J Comput Chem* **2011**, *32* (7), 1456-1465. https://doi.org/10.1002/jcc.21759.
103. Marenich, A. V.; Cramer, C. J.; Truhlar, D. G. Universal Solvation Model Based on Solute Electron Density and on a Continuum Model of the Solvent Defined by the Bulk Dielectric Constant and Atomic Surface Tensions. *Journal of Physical Chemistry B* **2009**, 113 (18), 6378–6396. https://doi.org/10.1021/JP810292N.
104. Cossi, M.; Rega, N.; Scalmani, G.; Barone, V. Energies, Structures, and Electronic Properties of Molecules in Solution with the C-PCM Solvation Model. *J. Comput. Chem.* **2003**, *24* (6), 669–681. https://doi.org/10.1002/JCC.10189.
105. Barone, V.; Cossi, M. Quantum Calculation of Molecular Energies and Energy Gradients in Solution by a Conductor Solvent Model. *Journal of Physical Chemistry A* **1998**, *102* (11), 1995–2001. https://doi.org/10.1021/jp9716997.
106. Riplinger, C.; Neese, F. An Efficient and near Linear Scaling Pair Natural Orbital Based Local Coupled Cluster Method. *Journal of Chemical Physics* **2013**, *138* (3), 34106. https://doi.org/10.1063/1.4773581/192513.
107. Neese, F.; Wennmohs, F.; Becker, U.; Riplinger, C. The ORCA Quantum Chemistry Program Package. *Journal of Chemical Physics* **2020**, *152* (22), 224108. https://doi.org/10.1063/5.0004608.
108. Neese, F. Software Update: The ORCA Program System—Version 5.0. *WIREs Comput Mol Sci. 2022, 12:e1606.* https://doi.org/10.1002/wcms.1606.
109. Dunning, T. H. Gaussian Basis Sets for Use in Correlated Molecular Calculations. I. The Atoms Boron through Neon and Hydrogen. *J Chem Phys* **1989**, *90* (2), 1007–1023. https://doi.org/10.1063/1.456153.
